# Effects of Genital Ulcer Disease and Herpes Simplex Virus Type 2 on the Efficacy of Male Circumcision for HIV Prevention: Analyses from the Rakai Trials

**DOI:** 10.1371/journal.pmed.1000187

**Published:** 2009-11-24

**Authors:** Ronald H. Gray, David Serwadda, Aaron A. R. Tobian, Michael Z. Chen, Frederick Makumbi, Tara Suntoke, Godfrey Kigozi, Fred Nalugoda, Boaz Iga, Thomas C. Quinn, Lawrence H. Moulton, Oliver Laeyendecker, Steven J. Reynolds, Xiangrong Kong, Maria J. Wawer

**Affiliations:** 1 Bloomberg School of Public Health, Johns Hopkins University, Baltimore, Maryland, United States of America; 2 School of Public Health, Makerere University, Kampala, Uganda; 3 Rakai Health Sciences Program, Entebbe, Uganda; 4 Department of Pathology, Johns Hopkins University School of Medicine, Baltimore, Maryland, United States of America; 5 Department of Medicine, Johns Hopkins University School of Medicine, Baltimore, Maryland, United States of America; 6 Division of Intramural Research, National Institute of Allergy and Infectious Diseases, National Institutes of Health, Bethesda, Maryland, United States of America; University of California Davis, United States of America

## Abstract

**Background:**

Randomized trials show that male circumcision (MC) reduces the incidence of HIV and herpes simplex virus type 2 (HSV-2) infections, and symptomatic genital ulcer disease (GUD). We assessed the role of GUD and HSV-2 in the protection against HIV afforded by MC.

**Methods and Findings:**

HIV-uninfected men were randomized to immediate (*n* = 2,756) or delayed MC (*n* = 2,775) in two randomized trials in Rakai, Uganda. GUD symptoms, HSV-2 status, and HIV acquisition were determined at enrollment and at 6, 12, and 24 mo of follow up. Ulcer etiology was assessed by PCR. We estimated the prevalence and prevalence risk ratios (PRRs) of GUD in circumcised versus uncircumcised men and assessed the effects of HSV-2 serostatus as a risk-modifying factor for GUD. We estimated the proportion of the effect of MC on HIV acquisition that was mediated by symptomatic GUD, and by HSV-2 infection. Circumcision significantly reduced symptomatic GUD in HSV-2-seronegative men (PRR = 0.51, 95% [confidence interval] CI 0.43–0.74), HSV-2-seropositive men (PRR = 0.66, 95% CI 0.51–0.69), and in HSV-2 seroconverters (PRR = 0.48, 95% CI 0.30–0.79). The proportion of acute ulcers due to HSV-2 detected by PCR was 48.0% in circumcised men and 39.3% in uncircumcised men (χ^2^ *p* = 0.62). Circumcision reduced the risk of HIV acquisition in HSV-2 seronegative men (incidence rate ratio [IRR] = 0.34, 95% CI 0.15–0.81), and potentially in HSV-2 seroconverters (IRR = 0.56, 95% CI 0.19–1.57; not significant), but not in men with prevalent HSV-2 at enrollment (IRR = 0.89, 95% CI 0.49–1.60). The proportion of reduced HIV acquisition in circumcised men mediated by reductions in symptomatic GUD was 11.2% (95% CI 5.0–38.0), and the proportion mediated by reduced HSV-2 incidence was 8.6% (95% CI −1.2 to 77.1).

**Conclusions:**

Circumcision reduced GUD irrespective of HSV-2 status, but this reduction played only a modest role in the protective effect of circumcision on HIV acquisition.

**NIH Trial registration:**

ClinicalTrials.gov NCT00425984

**Gates Foundation Trial registration:**

ClinicalTrials.gov NCT00124878

*Please see later in the article for the Editors' Summary*

## Abstract 2

Ron Gray and colleagues analyze data from two circumcision trials in Uganda to assess how HSV-2 status and genital ulcer disease affect the procedure's ability to reduce HIV infection.

## Editors' Summary

**Background:**

Acquired immunodeficiency syndrome (AIDS) has killed more than 25 million people since 1981, and more than 30 million people (22 million in sub-Saharan Africa alone) are now infected with the human immunodeficiency virus (HIV), which causes AIDS. There is no cure for HIV/AIDS. Consequently, prevention of HIV transmission is extremely important. Because HIV is most often spread through unprotected sex with an infected partner, individuals can reduce their risk of becoming infected with HIV by abstaining from sex, by having one or a few partners, and by always using a male or female condom. In addition, three large trials in sub-Saharan Africa (including one in Rakai, Uganda) recently reported that male circumcision—the removal of the foreskin, a loose fold of skin that covers the head of the penis—can halve HIV transmission rates in men. Thus, as part of its HIV prevention strategy, the World Health Organization now recommends that male circumcision programs be scaled up in countries where there is a generalized HIV epidemic and where few men are circumcised.

**Why Was This Study Done?:**

It is still not clear why male circumcision reduces HIV acquisition in men. Certainly, the foreskin contains many cells that HIV can infect and the foreskin's delicate lining is thought to be particularly vulnerable to HIV infection partly because intercourse can cause small tears in it through which HIV can enter the body. But male circumcision also reduces genital ulcer disease—sores on the penis and other genital organs caused by infection with several sexually transmitted organisms including the herpes simplex virus type 2 (HSV-2). Genital ulcer disease, particularly when caused by HSV-2, is thought to increase a person's risk of acquiring HIV, so could male circumcision reduce HIV transmission rates because of its beneficial effects on genital ulcer disease rather than through its removal of foreskin tissue with its rich source of HIV target cells? In this study, the researchers investigate this question by re-analyzing data collected in two Ugandan trials of male circumcision for HIV prevention.

**What Did the Researchers Do and Find?:**

In the Ugandan trials, the researchers randomly assigned about 5,500 HIV-uninfected men to immediate circumcision or to circumcision 24 months later. At enrollment, they asked the men whether they had any symptoms of genital ulcer disease (for example, a painful penile sore or genital itching), examined the men's genital areas, and took blood samples to test for HSV-2 infection. The researchers repeated these examinations and tests at 6 months, 12 months, and 24 months and tested the study participants for HIV infection. The researchers' statistical analysis of these data shows that circumcision approximately halved symptomatic genital ulcer disease in the study participants irrespective of their HSV-2 infection status. Circumcision reduced the risk of HIV acquisition in men without HSV-2 infection by two-thirds but did not affect HIV acquisition among men infected with HSV-2 at enrollment. Among the men who became infected with HSV-2 during the study, circumcision reduced the risk of HIV acquisition but this reduction in risk was not statistically significant. That is, it could have happened by chance. Finally, the researchers calculated that 11.2% of the observed reduction in HIV acquisition associated with circumcision was mediated by reductions in symptomatic genital ulcer disease and 8.6% was mediated by reductions in HSV-2 infections.

**What Do These Findings Mean?:**

The findings of this study are limited by the small number of people in some of the subgroups analyzed and by genital ulcer disease being self-reported. Furthermore, the validity of some of the findings may be compromised because the analysis described here was not specified in the original trial protocol. Nevertheless, these findings suggest that the reduction of genital ulceration following circumcision plays only a minor part in the ability of male circumcision to reduce HIV acquisition in men. They also suggest that circumcision reduces genital ulcer disease primarily by reducing the rate of nonherpetic ulceration, including sores caused by mild trauma during intercourse. Thus, the researchers conclude, most of the reduction in HIV acquisition provided by male circumcision may be attributable to the removal of vulnerable foreskin tissue containing HIV target cells.

**Additional Information:**

Please access these Web sites via the online version of this summary at http://dx.doi.org/10.1371/journal.pmed.1000187.

- Information is available from the US National Institute of Allergy and Infectious Diseases on HIV infection and AIDS
- HIV InSite has comprehensive information on all aspects of HIV/AIDS
- Information is available from Avert, an international AIDS charity on many aspects of HIV/AIDS, including information on HIV and AIDS in Uganda, and on circumcision and HIV (in English and Spanish)
- More information about male circumcision is available from the Clearinghouse on Male Circumcision
- Information on the Rakai HIV prevention trial is available
- The MedlinePlus Encyclopedia has a page on male genital sores (in English and Spanish)
- The US National Institute of Allergy and Infectious Diseases provides information about genital herpes
- The US Centers for Disease Control and Prevention also provides information on genital herpes (in English and Spanish)

## Introduction

Three randomized trials in South Africa [1], Kenya [2], and Uganda [3] found that male circumcision (MC) reduces the risk of HIV acquisition in men, a finding supported by prior observational studies [4]. The foreskin is rich in HIV target cells and the inner preputial mucosa is thought to be lightly keratinized and vulnerable to HIV infection [5]–[8]. Thus, it is hypothesized that circumcision may reduce HIV infection by removal of this vulnerable tissue. The Ugandan trial also found that circumcision decreased the rate of self-reported genital ulcer disease (GUD) [3] and the incidence of herpes simplex virus type 2 (HSV-2) [9]; these findings had previously been suggested by observational studies [10]. The South African trial reported that circumcision was equally protective against HIV acquisition in HSV-2 seropositive and seronegative men [11]. GUD, particularly due to HSV-2, is thought to be a cofactor for HIV acquisition [12]–[14]. Thus it is possible that circumcision prevents HIV in part by reducing genital ulceration and HSV-2.

We conducted a secondary data analysis from the randomized trials of MC for HIV prevention in Rakai, Uganda, to assess the degree to which circumcision-related reductions in symptomatic genital ulcers and HSV-2 potentially mediated the effect of MC on HIV prevention.

## Methods

We conducted two concurrent randomized trials that enrolled consenting uncircumcised men aged 15–49 y and randomized them to receive immediate circumcision (intervention arm), or circumcision delayed for 24 mo (control arm) (Text S3). Details have been published previously [3],[9]. Participants provided written informed consent for screening, enrollment, and follow up, and men receiving circumcision also provided written consent for surgery. Participants were followed at 6, 12, and 24 mo and interviewed with regard to sexual risk behaviors and self-reported genital ulceration during the preceding follow-up interval, as well as recent GUD symptoms within 1 wk of the study visit. In addition, a genital exam was performed and swabs taken from any observed penile lesion. One trial supported by the National Institutes of Health (NIH) enrolled HIV-negative men who, as condition for enrollment eligibility, accepted voluntary counseling and testing (VCT) and agreed to learn their HIV results. A second trial, supported by the Bill & Melinda Gates Foundation, enrolled HIV-negative men who accepted pre-test counseling but declined to learn their HIV results, and thus were ineligible for enrollment in the NIH trial. Under Ugandan policy persons may provide blood for testing but decline to learn their test result, and such individuals were not denied access to trial participation. All participants were offered intensive HIV prevention education, access to free HIV VCT, and condoms, provided free of charge, and were strongly encouraged at each study visit to practice safe sex behaviors and to avail themselves of VCT and condoms. The trials are registered with ClinicalTrials.gov numbers NCT00425984 for NIH trial and NCT00124878 for the Gates Foundation trial. The protocol (see Text S1 and Text S2) was reviewed and approved by the Uganda National Council for Science and Technology, and by three Institutional Review Boards (IRBs): the Science and Ethics Committee of the Uganda Virus Research Institute, Entebbe, Uganda; the Committee for Human Research at Johns Hopkins University, Bloomberg School of Public Health; and the Western Institutional Review Board, Olympia, Washington. The data were analyzed without personal identifiers.

**Figure 1:**
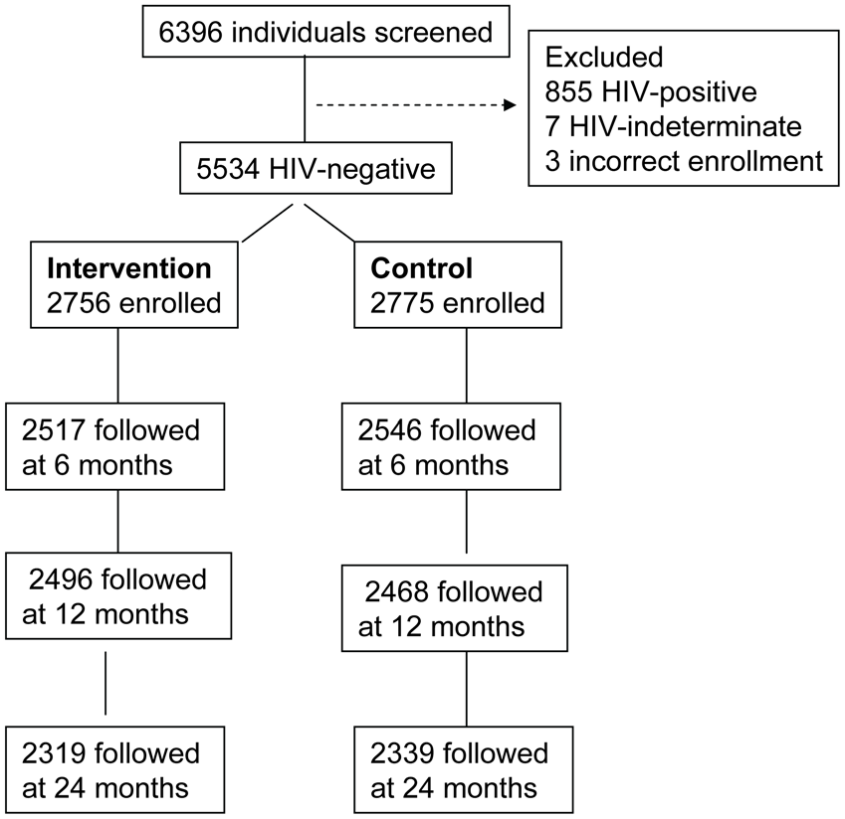
Trial profile.

The objective of this analysis was to assess the degree to which GUD and HSV-2 serostatus contributed to the reduced HIV incidence observed in circumcised men. This analysis used data from all initially HIV-uninfected men enrolled in either trial who contributed follow-up time for determination of incident HIV infection, and for whom we had HSV-2 serology at enrollment and follow up. The trial profile is given in Figure 1. 6,396 men were screened of whom 5,534 HIV-negative men were enrolled. There were a total of 2,756 HIV-negative men enrolled in the intervention arm of either trial and 2,775 men enrolled in the control arm of either trial available for this analysis. Of these enrolled participants, 2,319 intervention arm (84.1%) and 2,339 control arm (84.3%) were followed over 2 y.

The enrollment blood samples for these men were tested for HSV-2 antibodies using the HSV-2 ELISA (Kalon Biological Ltd.). On the basis of prior evaluation of test performance in Ugandan sera using HSV-2 University of Washington Western blot as the gold standard, HSV-2 positive status was defined as a Kalon ELISA index value of ≥1.5 [15]. Men with a Kalon index value ≤0.9 were classified as HSV-2 negative. Trial enrollment samples with Kalon index values between 0.90–1.49 were classified as indeterminate HSV-2 status. Only HSV-2 negative individuals were evaluated for HSV-2 seroconversion (index value ≥1.5). All seroconversions were confirmed by Euroimmun Western blot (Euroimmun). HSV-2 serostatus was classified into four mutually exclusive groups; HSV-2 positive at enrollment, HSV-2 indeterminate at enrollment, HSV-2 seronegative at enrollment and throughout follow up, and HSV-2 seroconversions. HIV status was determined by two separate ELISAs and confirmed by HIV-1 Western blot, as previously described [3]. Serologic syphilis was assessed at each study visit on the basis of a positive rapid plasma reagin (RPR) (Becton Dickinson) or toluidine red unheated serum test (TRUST) (New Horizons Diagnostics Corporation) and confirmed by a positive *Treponema pallidum* particle agglutination assay (TPPA) (Serodia-TP PA kit, Fujirebio Inc.).

For men who had a genital lesion observed at the time of a study visit, a swab was obtained for detection of *Haemophilus ducreyi*, *T. pallidum*, HSV-1, and HSV-2 using real-time multiplex PCR [16]–[18]. Two multiplex real-time PCR reactions using fluorescent probes for two pathogens in a single reaction (*H. ducreyi* and *T. pallidum* or HSV-1 and HSV-2), were performed on extracted DNA. Samples were run in duplicate, and discordant results were repeated in a single-pathogen real-time PCR reaction.

### Statistical Analysis

Enrollment sociodemographic and behavioral characteristics of intervention and control arm participants, and the characteristics of HSV-2 seropositive and seronegative men were compared. Differences between groups were assessed using Chi-square tests. At each study visit, we assessed the frequency of self-reported GUD during the preceding follow-up interval to estimate the period prevalence of GUD per 100 follow-up visits. Prevalence risk ratios (PRRs) and 95% confidence intervals (95% CI) estimated by modified Poisson regression [16] were used to assess differences in GUD frequency between study arms and strata of HSV-2 status. Because men could report multiple episodes of symptomatic ulceration in different follow-up intervals, robust variance estimates based on generalized estimating equations were used to account for within-individual correlation. We also assessed the point prevalence of GUD observed at the time of a study visit and the proportion of ulcer swabs with sexually transmitted infection (STI) pathogens detected by real-time PCR.

We determined HIV acquisition during each follow-up interval and cumulated interval-specific incident HIV events and person time over the period of observation. There were a total of 105 incident HIV infections, 36 in the intervention arm and 69 in the control arm participants. HIV incidence rates were estimated per 100 person years (py) assuming that infection occurred midway between the last HIV-negative and first HIV-positive serologic test. Differences in HIV incidence between study arms, stratified by HSV-2 status and GUD symptoms were assessed by incidence rate ratios (IRRs) and 95% CI estimated by Poisson multiple regression after adjustment for covariates found to be significantly associated with HIV acquisition in univariate analyses at *p*<0.15. The covariates used in adjusted models included age, marital status, number of sex partners, and condom use. Exact 95% CI were estimated if any cell contained less than five observations.

We hypothesized that GUD and incident HSV-2 could be intermediate factors between circumcision and HIV acquisition, because both are reduced by circumcision and are related to incident HIV. Therefore, we conducted a mediation analysis using two logistic regression models, one estimating the net effect of circumcision on HIV incidence without adjustment for the intermediate variables, and the second model estimating the direct effects of circumcision on HIV acquisition after adjustment for potential mediating variables (GUD or incident HSV-2) [19]. We then estimated the proportion of the circumcision effect on HIV acquisition explained by the effects of circumcision on intermediate variables using the expression 
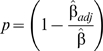
, where 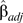 is the coefficient estimate of the circumcision effect from the model including the intermediate variable, and 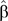 is the coefficient from the model without the intermediate variable. The confidence interval for *p* was obtained using bias corrected and accelerated (BCa) bootstrap confidence intervals. Specifically, since the incidence of HIV was rare, we used a stratified bootstrap method by bootstrapping in the HIV incidence samples and nonincidence samples, respectively. All the analyses were performed in R 2.8.1 environment. The whole trial population was assessed for the mediating effect of GUD, since circumcision was found to reduce GUD, irrespective of HSV-2 serostatus. To assess the mediating effect on incident HSV-2 infection, the population was restricted to men who were HSV-2 negative at enrollment and thus at risk of HSV-2 seroconversion during follow up.

We also estimated the attributable risk (AR) of incident HIV associated with GUD or HSV-2 using the formula 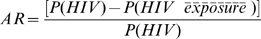 [20], where exposure is GUD or HSV-2 seroconversion, respectively. The standard errors of the AR were estimated from log (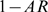) for 95% CI calculations. ARs of HIV acquisition associated with GUD or HSV-2 were estimated separately by study arm. To test the null hypothesis that the AR associated with GUD or HSV-2 is the same between study arms, the *p*-value for the difference in AR between arms was calculated by comparing the approximated normal distributions of log (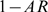) for the two arms.

## Results

Table 1 shows the distribution of participant characteristics at enrollment. HSV-2 prevalence was 28.3% (782/2,576) in the intervention arm and 27.4% (759/2,775) in the control arm. HSV-2 indeterminate serostatus (Kalon Elisa index values 1.0 to 1.49) was similar in intervention (10.1%, 278/2,756) and control arms (10.8%, 299/2,775), and 61.5% of intervention arm men (1696/2,756) versus 61.7% of control arm men (1,717/2,775) were HSV-seronegative at enrollment. The two study arms were also comparable with respect to sociodemographic and behavioral characteristics (Table 1). However, there were marked differences in the characteristics of men who were HSV-2 seropositive and seronegative at enrollment. Compared to HSV-2 negative men, HSV-2-positive men were significantly older (40+ y, 13.9% versus 2.6%, χ^2^ *p*<0.0001), more likely to be currently or previously married (77.2% versus 42.5%, χ^2^ *p*<0.0001), to have a higher number of reported sex partners in the past year (2+ 43.8% versus 31.8%, χ^2^ *p*<0.0001), and were less likely to consistently use condoms (7.9% versus 16.9%, χ^2^ *p*<0.0001).

**Table 1:** Characteristics of the study populations at enrollment.

	Enrollment HSV-2 Positive				Enrollment HSV-2 Negative				All Participants			
	Intervention		Control		Intervention		Control		Intervention		Control	
Characteristics and Behaviors	*n*	Percent	*n*	Percent	*n*	Percent	*n*	Percent	*n*	Percent	*n*	Percent
**All**	782	100.0	759	100.0	1,696	100.0	1,717	100.0	2,756	100.0	2,775	100.0
**Age (y)**												
**15–29**	412	52.7	389	51.2	1,379	81.3	1,430	83.2	1,988	72.1	2,037	73.4
**30–39**	267	34.1	261	34.3	269	15.9	247	14.4	598	21.7	575	20.7
**40+**	103	13.2	110	14.5	48	2.8	41	2.4	170	6.2	163	5.9
**Marital status**												
**Currently married**	534	68.3	519	68.4	658	38.8	654	38.1	1,340	48.6	1,332	48.0
**Previously married**	65	8.3	71	9.4	82	4.8	56	3.3	165	6.0	141	5.1
**Never married**	183	23.4	169	22.3	956	56.4	1,007	58.7	1,251	45.4	1,302	46.9
**Sex partners in past year**												
**None**	74	9.5	63	8.3	363	21.4	402	23.4	493	17.9	518	18.7
**One**	368	47.1	362	47.7	786	46.3	776	45.2	1,288	46.7	1,279	46.1
**Two**	219	28.0	237	31.2	348	20.5	367	21.4	621	22.5	670	24.1
**Three or more**	121	15.5	97	12.8	199	11.7	172	10.0	354	12.8	308	11.1
**Condom use**												
**None**	469	60.0	436	57.4	942	55.5	957	55.7	1,589	57.7	1,564	57.7
**Inconsistent**	254	32.5	261	34.4	466	27.5	474	27.6	791	28.7	826	29.8
**Consistent**	59	7.5	62	8.2	288	17.0	286	16.7	376	13.6	385	13.9

As shown in Table 2, the period prevalence of self-reported GUD per 100 study visits was significantly lower in the intervention than the control arm men (PRR 0.59, 95% CI 0.50–0.69, Wald test, *p*<0.0001). In a multivariate model, adjusted for age, number of sexual partners, and condom use, the PRR of GUD associated with circumcision was 0.54 (95% CI 0.44–0.66). Other covariates significantly associated with lower symptomatic GUD were having no sex partners during the follow-up interval (PRR = 0.26, 95% CI 0.16–0.42) and consistent condom use (PRR = 0.63, 95% CI 0.43–0.92). The prevalence of self-reported GUD was significantly lower in the intervention than the control arm irrespective of HSV-2 status at enrollment or HSV-2 acquisition during follow up, although the highest rates of symptomatic GUD were reported among men who seroconverted to HSV-2 during the trial.

**Table 2:** Self reported genital ulceration by enrollment HSV-2 status and study arm.

	Intervention		Control		
HSV-2 Status at Enrollment	GUD/*n* Visits	GUD Percent	GUD/*n* Visits	GUD Percent	PRR Intervention/Control (95% CI)
**All**	212/7,332	2.9	361/7,353	4.9	0.59 (0.50–0.69)
**HSV-2 Status**					
**HSV-2 positive**	91/2,111	4.3	134/2,056	6.5	0.66 (0.51–0.84)
**HSV-2 indeterminate**	29/753	3.9	48/785	6.1	0.63 (0.41–0.96)
**HSV-2 seroconverters**	18/336	5.4	50/452	11.1	0.48 (0.30–0.79)
**Persistent HSV-2 negative**	74/4,132	1.8	129/4,060	3.2	0.51 (0.43–0.74)

The point prevalence of clinically observed GUD reported at the time of a study visit was 0.8% in the intervention arm (53/6,554 visits) and 1.9% (121/6,544 visits) in the control arm (PRR = 0.44, 95% CI 0.32–0.61). Swabs for multiplex PCR were obtained from visible lesions in 25 symptomatic intervention arm men and 56 symptomatic control arm men. HSV-2 was detected in 48.0% (12/25) of GUD swabs assayed among symptomatic circumcised men, and 39.4% (22/56) of swabs assayed for symptomatic uncircumcised men (χ^2^ *p* = 0.62). No *T. pallidum* was detected by PCR in intervention arm men, whereas seven *T. pallidum* infections were found among control arm participants (9.6%, χ^2^ *p* = 0.09). No cases of ulcers due to *H. ducreyi* or HSV-1 were detected in either study arm. It is noteworthy that of those symptomatic participants tested by PCR, no STI pathogen was detected in 52.0% of 25 intervention arm men and 60.7% of 56 control arm men, suggesting that many observed lesions did not have an STI etiology.

There were 202 intervention arm men and 195 control arm men with positive syphilis serology during follow-up visits. Among these individuals, GUD symptoms were reported during the follow-up interval preceding serologic syphilis detection in 4.5% of visits by intervention arm men (9/202), and in 13.3% of visits in by control arm men (26/195, PRR = 0.33, 95% CI 0.16–0.69, χ^2^ *p* = 0.003). Among men who were *T. pallidum* seronegative throughout the study, the frequency of GUD symptoms was 3.2% (226/7,942 visits) in intervention arm men and 5.8% (413/7,095 visits) in control arm men (PRR = 0.55, 95% CI 0.47–0.65).

HIV incidence is shown in Table 3. HIV incidence was 0.78/100 py among circumcised men and 1.44/100 py among uncircumcised men (IRR = 0.54, 95% CI 0.35–0.78). HIV incidence was lower in circumcised than in uncircumcised men with self-reported symptomatic GUD (IRR = 0.55, 95% CI 0.20–1.50), although this difference was not statistically significant. However, circumcision was significantly protective against HIV acquisition in men without GUD symptoms (IRR = 0.57, 95% CI 0.37–0.89). We did not observe a reduction in the risk of HIV acquisition among men who were HSV-2 positive at enrollment (IRR = 0.89, 95% CI 0.49–1.60), irrespective of whether the prevalent HSV-2 positive men reported GUD symptoms (IRR = 0.92, 95% CI 0.15–4.90) or were asymptomatic (IRR = 0.94, 95% CI 0.49–1.81). The interaction between HSV-2 seropositive status and treatment arm was not statistically significant (likelihood ratio *p* = 0.07).

**Table 3:** HIV incidence by self-reported GUD and HSV-2 status and study arm.

	Intervention		Control		
HSV-2 Status at Enrollment	HIV Cases/py	HIV Incidence/100 py	HIV Cases/py	HIV Incidence/100 py	IRR Intervention/Control (95% CI)
**All**	36/4,825.5	0.78	69/4,846.0	1.44	0.54 (0.35–0.78)
**GUD symptoms**	5/173	2.89	16/303.5	5.27	0.55 (0.20–1.50)
**No GUD**	31/4,652.5	0.70	53/4,542.5	1.18	0.57 (0.37–0.89)
**HSV-2 positive**					
**All**	21/1,392	1.51	23/1,358.5	1.69	0.89 (0.49–1.60)
**GUD symptoms**	3/74	4.1	5/117.5	4.26	0.92 (0.15–4.90)
**No GUD**	18/1,318	1.37	18/1,241	1.45	0.94 (0.49–1.81)
**HSV-2 indeterminate**					
**All**	3/501	0.60	14/519	2.70	0.22 (0.04–0.80)
**GUD symptoms**	1/25	4.00	3/44	6.82	0.59 (0.01–7.31)
**No GUD**	2/476	0.83	11/475	2.32	0.18 (0.02–0.83)
**HSV-2 seroconverters**					
**All**	5/221	2.26	12/296.5	4.05	0.56 (0.19–1.57)
**GUD symptoms**	1/15.5	6.45	4/40.5	9.88	0.65 (0.01–6.60)
**No GUD**	4/205.5	1.95	8/256	3.13	0.62 (0.14–2.32)
**HSV-2 negative**					
**All**	7/2,711.5	0.26	20/2,672	0.77	0.34 (0.15–0.81)
**GUD symptoms**	0/58.5	0	4/101.5	3.94	0.0 (0.0–2.63)
**No GUD**	7/2,653	0.26	16/2,570.5	0.64	0.42 (0.17–1.03)

95% CI based on an exact test when cell size was <5 observations.

Because these findings were contrary to those reported in the South African trial, which observed circumcision efficacy against HIV acquisition irrespective of HSV-2 serostatus [11], we conducted a sensitivity analysis confined to the age group enrolled in the South African trial (men aged 18–24 y), and using the same Kalon index value of ≥1.1 for HSV-2 seropositivity as was used in the South African study. Using these criteria, HIV incidence was 1.02/100 py (7/688.8 py) in the intervention arm and 2.04 (14/686.5 py) in the control arm (IRR = 0.50, 95% CI 0.20–1.23), which is compatible with the protective effect of circumcision among the HSV-2 positive men in the South African trial (0.37, 95% CI 0.09–1.55) [11].

HIV incidence was highest among HSV-2 seroconverters, particularly if they reported GUD symptoms, and the risk of HIV acquisition was lower but not statistically significant in circumcised compared to uncircumcised men who acquired HSV-2 (IRR = 0.56, 95% CI 0.19–1.57). The protective effects of circumcision against incident HIV was most pronounced and statistically significant in persistent HSV-2 negative men (IRR = 0.34, 95% CI 0.15–0.81). The numbers of HSV-2 negative men with GUD were too small to estimate efficacy. The adjusted incidence rate ratios (adjIRR) of HIV acquisition based on Poisson multiple regression, were 0.58 (95% CI 0.39–0.87) for circumcision, and 3.11 (95% CI 1.90–5.10) for GUD. Using persistent HSV-2 negative men as the referent category, the adjIRRs of HIV acquisition were 3.15 (95% CI 1.86–5.31) for enrollment HSV-2 positives, 4.81 (95% CI 2.62–8.84) for HSV-2 seroconverters, and 3.02 (95% CI 1.63–5.59) for men with indeterminate HSV-2 status. Previously married men were also at higher risk of HIV (adjIRR 2.42, 95% CI 1.28–4.56).

Using mediation analysis, the estimated proportion of the HIV incident effect of circumcision explained by the reduction in symptomatic GUD among the circumcised men was 11.2% (1−[−0.5451/−0.6140], with 95% CI 5.0–38.0. There were 3,413 men who were HSV-2 negative at enrollment, and among this subpopulation, the adjusted proportion of HIV incident infections estimated to be mediated via HSV-2 seroconversion was 8.6% (95% CI −1.2 to 77.7). The confidence intervals are wide due to small number of HSV-2 seroconversions, and this mediated proportion estimate was not statistically significant.

Based on the data in Table 3, HIV incidence among circumcised men reporting GUD symptoms was 2.89/100 py compared with asymptomatic circumcised men (0.70/100 py), and the IRR of HIV acquisition associated with GUD was 4.12. The AR of HIV acquisition due to GUD in circumcised men was 10.7% (95% CI −1.8 to −21.6). Similarly, among uncircumcised men, HIV incidence was 5.27/100 py in those with GUD symptoms compared with 1.18/100 py in those without symptoms (IRR = 4.47). The AR of HIV acquisition attributable to GUD in uncircumcised men was 18.1% (95% CI 6.8–27.9). Therefore, ulceration contributed to a higher risk of HIV acquisition in both uncircumcised participants and circumcised participants, but confidence intervals were wide and the ARs of GUD were not significantly different between the two arms (χ^2^, *p* = 0.34).

Similarly, we estimated the AR of HIV acquisition attributable to HSV-2 seroconversion (compared to HSV-2 persistent seronegative). In the intervention arm, the IRR of HIV acquisition relative to persistent HSV-2 seronegative men was 8.69 (2.26/0.26), and the estimate of AR was 36.9% (95% CI −1.7 to −60.8%). In the control arm, the comparable IRR was 5.26 (4.05/0.77), and the AR was 30.6% (95% CI 9.3–46.8%). This suggests that HSV-2 seroconversion was associated with increased risk of HIV acquisition in both the intervention and control arms, but the ARs between the two arms were not significantly different (*p* = 0.63). Similar AR estimates for HSV-2 infections prevalent at time of enrollment were: intervention arm 17.1%, 95% CI 24.3–44.7, and control arm 29.8%, 95% CI 7.1–46.9, *p* = 0.63.

## Discussion

MC reduced symptomatic genital ulceration by 41%, and the protection against GUD was similar irrespective of HSV-2 status in this population (Table 2). We estimate that approximately 11.2% of the protection from HIV afforded by circumcision is mediated by the reduction of symptomatic GUD due to circumcision (Table 3). Thus, it is likely that the reduction in symptomatic GUD made only a modest contribution to the overall impact of circumcision on prevention of HIV infection.

The finding that circumcision reduced GUD in the men who remained HSV-2 seronegative throughout the trial to approximately the same degree that it reduced GUD in HSV-2 seropositive men (Table 2) suggests that circumcision primarily reduces the rate of nonherpetic ulceration. It is noteworthy that no STI pathogens were detected in approximately 60% of ulcer swabs tested by real-time PCR. Low rates of STI detection in ulcers have also been reported among STD clinic patients with symptomatic GUD [17], suggesting that a substantial proportion of ulcers are not due to sexually transmitted organisms. It is possible that such non-STI ulcers are due to trauma with secondary infections by other pathogens. Such traumatic lesions may be more common in uncircumcised men because the foreskin is retracted over the shaft of the penis during intercourse and minor trauma, particularly to the frenulum, is thought to be common [5]. Therefore, it is possible that circumcision protects men from GUD largely by preventing traumatic lesions.

We were surprised that circumcision did not significantly reduce HIV acquisition among men who had prevalent HSV-2 at enrollment, whereas the procedure was associated with lower HIV risk in other groups, including men with HSV-2 indeterminate status and (although not significantly) in seroconverters (Table 3). It is possible that our failure to observe a protective effect of circumcision in prevalent HSV-2 positive enrollees is due to chance since this was a post hoc subgroup analysis that was not specified in the protocol. Our finding is contrary to that reported from the South African circumcision trial that observed similar efficacy of circumcision for HIV prevention among HSV-2 seroprevalent and HSV-2 seronegative men [11],[21]. However, there were differences between the trials in the age of study participants (18–24 y in South Africa versus 15–49 y in Rakai), the definition of HSV-2 infection (Kalon index value of 1.1 in South Africa, compared to 1.5 in Rakai based on our prior studies [15]), and in HSV-2 prevalence (5.9% in the South African trial, 27.9% in Rakai). Sensitivity analyses of the Rakai data using the same age groups and HSV-2 diagnostic criteria as the South African trial indicated comparable and nonstatistically significant estimates of circumcision efficacy for HIV prevention (South Africa IRR = 0.37, 95% CI 0.09–1.55, Rakai IRR = 0.50, 95% CI 0.20–1.23). These young men were likely to have more recently acquired HSV-2, and thus potentially have active herpetic infection. Therefore, one potential explanation for the divergence of findings between the Ugandan and South African studies may be attenuated efficacy of MC for HIV prevention in older men with more chronic herpes infections, due to recruitment of CD4+ T cells and immature dendritic cells into areas of HSV-2 replication even months after resolution of lesions [22],[23]. This persistence of HIV receptor cells might explain why trials of HSV-2 suppressive therapy did not show efficacy for HIV prevention [24],[25], and reinforces the need for circumcised men to maintain safe sex practices (e.g., condom use or partner reduction) to avoid infection with HIV or STIs.

There are limitations to this study—a secondary analysis of two randomized trials that had not been prespecified in the trial protocols. The analysis was constrained by small numbers of observation in some subgroups, resulting in imprecise estimates. To increase sample size, we pooled data from HIV-negative men in two parallel trials, and although trial-specific findings were similar, sample size constraints precluded many trial-specific subgroup analyses (unpublished data). Self-reported symptomatic GUD is subject to misclassification because respondents may fail to recognize an ulcer or fail to recall it at time of interview. Also, although men were asked about GUD symptoms in the preceding interval, they were not asked about the duration of symptoms or multiple episodes of ulceration, so severe recurrent ulcers are likely to be underestimated. It is noteworthy that the efficacy of circumcision for prevention of clinically observed GUD at time of a study visit (PRR = 0.44, 95% CI 0.32–0.61), was greater than the efficacy estimated from the period prevalence of self-reported symptoms (PRR = 0.59, 95% CI 0.50–0.69), suggesting that the latter may have been affected by misclassification because of misreporting of symptoms.

In summary, we found that genital ulceration played only a modest role in protection from HIV due to MC, and that the reduction in self-reported GUD was observed in HSV-2 seronegative men, suggesting that the ulcers prevented by circumcision are likely to be nonherpetic in origin. Thus, the evidence suggests that most of the reduction in HIV risk afforded by circumcision is attributable to removal of vulnerable foreskin tissue containing HIV target cells.

## Supporting Information

Text S1 **NIH Trial protocol.** Protocol for the trial of circumcision in HIV-negative men who agreed to receive post-test VCT.
(0.59 MB DOC)

Text S2 **Gates Trial protocol.** Protocol for trial of MC in HIV-positive men and their partners, and HIV-negative men who declined post-test VCT.
(0.73 MB DOC)

Text S3 **CONSORT checklist.**
(0.19 MB DOC)

## Abbreviations

AR: attributable risk
CI: confidence interval
GUD: genital ulcer disease
HSV-2: herpes virus type 2
IRR: incidence rate ratio
MC: male circumcision
PRR: prevalence; risk ratio
py: person years
STI: sexually transmitted infection
VCT: voluntary counseling and testing

## References

[1] Auvert B, Taljaard D, Lagarde E, Sobngwi-Tambekou J, Sitta R (2005). Randomized, controlled intervention trial of male circumcision for reduction of HIV infection risk: the ANRS 1265 Trial.. PLoS Med.

[2] Bailey RC, Moses S, Parker CB, Agot K, Maclean I (2007). Male circumcision for HIV prevention in young men in Kisumu, Kenya: a randomised controlled trial.. Lancet.

[3] Gray RH, Kigozi G, Serwadda D, Makumbi F, Watya S (2007). Male circumcision for HIV prevention in men in Rakai, Uganda: a randomised trial.. Lancet.

[4] Weiss HA, Quigley MA, Hayes RJ (2000). Male circumcision and risk of HIV infection in sub-Saharan Africa: a systematic review and meta-analysis.. Aids.

[5] Szabo R, Short RV (2000). How does male circumcision protect against HIV infection?. BMJ.

[6] Patterson BK, Landay A, Siegel JN, Flener Z, Pessis D (2002). Susceptibility to human immunodeficiency virus-1 infection of human foreskin and cervical tissue grown in explant culture.. Am J Pathol.

[7] McCoombe SG, Short RV (2006). Potential HIV-1 target cells in the human penis.. Aids.

[8] Donoval BA, Landay AL, Moses S, Agot K, Ndinya-Achola JO (2006). HIV-1 target cells in foreskins of African men with varying histories of sexually transmitted infections.. Am J Clin Pathol.

[9] Tobian AA, Serwadda D, Quinn TC, Kigozi G, Gravitt PE (2009). Male circumcision for the prevention of HSV-2 and HPV infections and syphilis.. N Engl J Med.

[10] Weiss HA, Thomas SL, Munabi SK, Hayes RJ (2006). Male circumcision and risk of syphilis, chancroid, and genital herpes: a systematic review and meta-analysis.. Sex Transm Infect.

[11] Sobngwi-Tambekou J, Taljaard D, Lissouba P, Zarca K, Puren A (2009). Effect of HSV-2 serostatus on acquisition of HIV by young men: results of a longitudinal study in Orange Farm, South Africa.. J Infect Dis.

[12] Rottingen JA, Cameron DW, Garnett GP (2001). A systematic review of the epidemiologic interactions between classic sexually transmitted diseases and HIV: how much really is known?. Sex Transm Dis.

[13] Wald A, Link K (2002). Risk of human immunodeficiency virus infection in herpes simplex virus type 2-seropositive persons: a meta-analysis.. J Infect Dis.

[14] Corey L (2007). Synergistic copathogens–HIV-1 and HSV-2.. N Engl J Med.

[15] Gamiel JL, Tobian AAR, Laeyendecker OB, Reynolds SJ, Morrow RA (2008). Improved performance of enzyme-linked immunosorbent assays and the effect of human immunodeficiency virus coinfection on the serologic detection of herpes simplex virus type 2 in Rakai, Uganda.. Clin Vaccine Immunol.

[16] Barros AJ, Hirakata VN (2003). Alternatives for logistic regression in cross-sectional studies: an empirical comparison of models that directly estimate the prevalence ratio.. BMC Med Res Methodol.

[17] Risbud A, Chan-Tack K, Gadkari D, Gangakhedkar RR, Shepherd ME (1999). The etiology of genital ulcer disease by multiplex polymerase chain reaction and relationship to HIV infection among patients attending sexually transmitted disease clinics in Pune, India.. Sex Transm Dis.

[18] Orle KA, Gates CA, Martin DH, Body BA, Weiss JB (1996). Simultaneous PCR detection of Haemophilus ducreyi, Treponema pallidum, and herpes simplex virus types 1 and 2 from genital ulcers.. J Clin Microbiol.

[19] Freedman LS, Graubard BI, Schatzkin A (1992). Statistical validation of intermediate endpoints for chronic diseases.. Stat Med.

[20] Jewell NP (2004). Statistics for epidemiology.

[21] Sobngwi-Tambekou J, Taljaard D, Lissouba P, Zarca K, Puren A (2009). Effect of HSV-2 serostatus on acquisition of HIV by young men: results of a longitudinal study in Orange Farm, South Africa.. J Infect Dis.

[22] Gupta R, Warren T, Wald A (2009). Genital herpes.. Lancet.

[23] Zhu J, Hladik F, Woodward A, Klock A, Peng T (2009). Persistence of HIV-1 receptor-positive cells after HSV-2 reactivation is a potential mechanism for increased HIV-1 acquisition.. Nat Med.

[24] Celum C, Wald A, Hughes J, Sanchez J, Reid S (2008). Effect of aciclovir on HIV-1 acquisition in herpes simplex virus 2 seropositive women and men who have sex with men: a randomised, double-blind, placebo-controlled trial.. Lancet.

[25] Watson-Jones D, Weiss HA, Rusizoka M, Changalucha J, Baisley K (2008). Effect of herpes simplex suppression on incidence of HIV among women in Tanzania.. N Engl J Med.

