# Supplementary material for: Effects of Genital Ulcer Disease and Herpes Simplex Virus Type 2 on the Efficacy of Male Circumcision for HIV Prevention: Analyses from the Rakai Trials
Source: PLoS Med. 2009 Nov 24;6(11):e1000187. doi: 10.1371/journal.pmed.1000187 (PMC2771764; doi:10.1371/journal.pmed.1000187)
Supplement: Text S1 — NIH Trial protocol. Protocol for the trial of circumcision in HIV-negative men who agreed to receive post-test VCT. (0.59 MB DOC) [file pmed.1000187.s001.doc]

**Male Circumcision for HIV Prevention, Rakai-Uganda**

**U1 AI51171-01-A2**

**PROTOCOL**

Principal Investigator: Ronald H. Gray, Johns Hopkins University

Co-Investigators: Maria J. Wawer, Columbia University

Stephen Gange, Johns Hopkins University

David Serwadda, Makerere University

Nelson Sewankambo, Makerere university

Fred Wabwire-Mangen, Makerere University

Tom Lutalo, Uganda Virus Research institute

Stephen Watya, Makerere University

Noah Kiwanuka, Rakai Health Sciences Program

Godfrey Kigozi, Rakai Health Sciences Program

Fred Nalugoda, Rakai Health Sciences Program

Consultants Bernard Lo, UCSF

Roberta Heath, Johns Hopkins University

Study Site: Rakai District, Uganda

Period of Study 7/1/02 to 6/20/07

Contact Address

Ronald H. Gray MD MSc

Robertson professor of Reproductive Epidemiology

E 4547

Johns hopkins University

Bloomberg School of Public Health

615 N. Wolfe Street

Baltimore

MD 21205

Tel. 410-955-7818

Fax. 410-614-7386

E-mail rgray@jhsph.edu

**TABLE OF CONTENTS**

**1.0 INTRODUCTION**

1.1 Background 6

1.2 Rationale 7

1.3 Study Design 7

**2.0 STUDY OBJECTIVES**

2.2 Stage 2 Objectives 8

2.3 Time Line 9

**3.0 SELECTION AND ENROLLMENT OF SUBJECTS**

3.1 Population 9

3.2 Inclusion Criteria 9

3.3 Exclusion Criteria 9

3.4 Enrollment/Randomization Procedures 10

3.4.a Community Mobilization 10

3.4.b Screening and Enrollment, Informed Consent 10

3.4.c Procedure at Enrollment 11

3.4.e Randomization 11

**4.0 STUDY TREATMENT**

4.1 Surgery 12

4.2 Concomitant Medications 14

**5.0 CLINICAL AND LABORATORY EVALUATIONS**

5.1 Screening Evaluations 15

5.1.a Screening Interview and Exam 15

5.1.b Eligibility 15

5.1.c Enrollment and Randomization 15

5.1.d Sociodemographic and Behavioral Interview 16

5.2 Evaluations During Treatment

5.2.a Preoperative Assessment 16

5.2.b Surgery 16

5.3 Evaluations After Treatment

5.3.a Post-operative Evaluations 16

5.4a Scheduled Follow up- Both arms

5.4b Evaluations at Time of Premature Discontinuation 17

5.5 Laboratory Methods for Evaluation 17

5.6 Behavioral Research Methods for Evaluation 18

**6.0 DATA COLLECTION, SITE MONITORING AND ADVERSE**

**EXPERIENCE REPORTING**

6.1. Records to Be Kept 19

6.2 Data Management 19

6.3 Clinical Site Monitoring and Record Availability 20

6.4 Serious Adverse Event Reporting 20

6.4.a General Definitions 20

6.4.b Severity of Adverse Events 20

6.4.c Relationship of AEs to Intervention 21

6.4.d Study Definitions of Adverse Events 21

6.4.e Management of Adverse Events 25

6.4.f Reporting of Aes 25

6.4.g Tabulations of Adverse Events 25

6.4.h Source Documentation 26

**7.0 STATISTICAL CONSIDERATIONS**

7.2 Stage 2 Study Endpoints 26

7.2.a Primary Endpoint HIV Incidence 26

7.2.b Male STDs and STD Symptoms 27

7.2.c Safety of Circumcision 28

7.2.d Behavioral Disinhibition 28

7.3 Accrual and Feasibility 29

7.4 Randomization and Stratification 29

7.5 Analysis Plan 30

7.5.b Stage 2 Analysis Plan 30

7.6 Interim Data Safety and Monitoring 31

**8.0 HUMAN SUBJECTS**

8.1 Project Services of Relevance to Human Subjects Consideration 32

8.2 Institutional Review Boards 33

8.3 Training in Research Ethics 34

8.4 Proposed Involvement of Human Subjects 34

8.5 Recruitment and Consent 34

8.6 Ethnic Groups and Minors 35

8.7 Potential Risk to Participants 36

8.8 Procedures for Protecting Against Risk 36

8.9 Study Benefits to participants 38

8.9.d Considerations of Antiretroviral Therapy 38

8.10 Compensation for Participants 39

8.11 Benefit-Risk Ratio 40

**9.0 PUBLICATION OF RESEARCH FINDINGS** 40

Figures and Tables 41

**10. APPENDICES**

**CASE REPORT FORMS- 2^ND^ STAGE**

00.Registration form.

01.Screening Interview and Exam.

02.a. Pre-baseline Interview

02.b. Enrolment Interview

03.Pre- operative Interview and Exam

04.Surgical and postoperative record

05.First day POP follow up Interview and Exam

07.First week POP follow up Interview and Exam

08.Second week POP follow up Interview and Exam

09.a. Third week POP follow up Interview and Exam

09.b. Fourth week POP follow up Interview and Exam-Immediate

09.c. Fourth week follow up Interview and Exam -Delayed

09.d. Fifth week POP follow up Interview and Exam

09.e. Sixth week POP follow up Interview and Exam.

10.Syphilis result form

11a. Adverse Event report form

11b. Adverse Event clinical monitoring form

12a. Sixth month follow up questionnaire

12b.12 Monthly Interview and Exam

12c. 24 Monthly Interview and Exam

13.Protocol deviation form

14.Withdrawal form

15. Missed visit/ Refusal.

16.Unscheduled visit form

17.Visit Track card

18.Randomization sheet

19.a. Screening verification flow sheet.

19.b. Enrolment verification flow sheet

19.c. Surgical verification flow sheet.

19.d. FUP verification flow sheet

20.Emergency Equipment inventory checklist.

21.a. NIH rejection form

22.Eligibility Verification form

23.Sterilizer QA Record

24.Circumcision instrument set

25.Communication Flag

27.Daily productivity log sheet

28.Linkage form.

29.VCT Result communication form

30.VCT Counselor’s checklist

31. Pre-test counseling communication form

32.VCT Result letter.

33.Mobilization/Health education guide.

34.Sterilizer Record log

35.MOH-STD FORM 4 (Partner notification)

36.Appointment card

37.Error detection sheet

38.Re-interview/Tape recording sheet

39.Certification assessment form

40.Verbal autopsy.

41.Verify biological monitoring card

41.a. Verify biological monitoring control card

42.Invitation to enroll in RCCS

43.Comprehension test interview

44.Spouse referral form

45.RCCS-Circumcision referral form.

46. Postoperative Patient information sheet

48.RCCS FUP Interview

Lab Form # 001 Specimen collection form.

Lab Form # 002 HemoCue Collection and Result Form

Lab Form # 003 Rapid HIV testing worksheet.

Lab Form # 004(a) Blood receipt form

Lab Form # 004(b) Urine receipt form.

Lab Form # 004(c) PCR swabs log form

Lab Form # 004(d) HPV shipper log form.

Lab Form # 005(a) RPR card test.

Lab Form # 005(b) TPPA confirmatory test.

Lab Form # 006 Wound infection culture and Gram stain results form

Lab Form # 007 Foreskin log form

Lab Form # 015 Participant result form.

**CONSENT FORMS.**

1. Screening consent

I.a. Enrollment consent NIH

II.a. Post randomization Information sheet- Immediate

II.b. Post randomization Information sheet- Delayed

III Surgical consent Form

IV Control subjects consent for surgery

V RCCS Baseline Consent

VI RCCS Follow-Up Consent

**Other**

23. Theater Logs and Procedures

24. Literature Cited

25. Study Site and Experience of Investigators

26. Roster of Protocol Team Members

**1.0 INTRODUCTION**

- 1. **Background**

Three randomized trials in South Africa, Kenya and Uganda have shown efficacy of male circumcision for HIV prevention in varying age groups (18-24 and 15-49 years), and geographic settings (urban, peri-urban and rural populations)**.** The amendment to this protocol is to provide surgery to control participants enrolled in the Rakai trial

**Circumcision in HIV-Negative Men and Risks of HIV/STD Acquisition**

***Circumcision and HIV risks in HIV-negative men:***

Three randomized trials have shown that circumcision reduces male HIV incidence by 50-60%. Many, but not all cross-sectional epidemiologic studies, particularly from Africa, show lower HIV prevalence rates among circumcised compared with uncircumcised heterosexual men.^1-8, 23-27^ In addition, ecologic analyses suggest that the distribution of prevalent HIV is inversely correlated with the prevalence of male circumcision.^28-29b^ A meta-analysis which included 18 cross-sectional or case-control studies of prevalent HIV and 3 cohort studies (two from the Rakai Health Sciences Program), estimated an adjusted relative risk of HIV infection in circumcised relative to uncircumcised men of 0.42 (CI 0.34-0.52).^3^ The reduction in risk was even more pronounced in men with high HIV exposure (RR=0.29, CI 0.20-0.41).^3^ In Rakai, HIV risks were lower in circumcised men both for incident infection (RR=0.53, CI0.3-0.9)^1^ and prevalent HIV (OR=0.41, CI 0.3-0.5).^2^ The protective effects were most marked in highly exposed men in HIV-discordant couples: within this subgroup, there were no seroconversion in circumcised HIV-negative men, compared with an incidence of 16.7 per 100py in uncircumcised men (p = 0.0004).^1,30^ A study of high risk trucking company employees in Kenya also found reduced HIV acquisition in circumcised men (RR= 0.25, CI 0.1-0.53).^5,31^ In Kenyan men with GUD, HIV incidence was lower among those who were circumcised (RR=0.41, CI 0.23-0.93).^5a^ Circumcision is also associated with a lower risk of HIV in homosexual men.^32^

In Rakai, the protective effects of circumcision are most pronounced among men in whom the procedure was performed before puberty. Compared with uncircumcised men, pre-pubertal circumcision ( 12 years) was associated with significantly reduced risks of prevalent HIV (OR = 0.39, CI 0.29-0.53), as was circumcision performed between the ages 13-20 years (OR = 0.46, CI 0.28-0.77). However, risks of prevalent infection were not significantly reduced with circumcision after age 20 (OR= 0.78 (CI 0.42-1.43).^2^ Circumcision before puberty was also associated with a significantly lower rate ratio of incident HIV (RR = 0.49, CI 0.3-0.8), compared with uncircumcised men, whereas circumcisions after age 12 years was not significantly protective (RR = 0.70, CI 0.3-1.6).^1^ The apparent attenuation of protective effects with older age of circumcision probably reflects confounding by medical indication. In Rakai, 75% of post-pubertal circumcisions were performed for health indications (e.g., phimosis), and it is likely that such pathology is, in part, a consequence of prior STD infections, which are markers for high risk behaviors.^1^ However, it is also possible that scar formation following adult circumcision may result in more friable tissue and less keratinization than with pre-pubertal circumcision, and a friable scar might increase vulnerability to HIV infection among men circumcised in adulthood, particularly if intercourse is resumed before complete wound healing. It is also possible that preexisting infections, which are indications for adult circumcision, may retard healing. We do not know of other data on HIV risk in relation to age of circumcision or indications for the procedure, nor are we aware of information on scar formation or whether healing is delayed by preexisting infections.^9^ Thus, although adolescent or adult circumcision is likely to be protective against HIV, observational data cannot quantify the magnitude of this effect, due to probable confounding by indication and factors affecting postoperative tissue repair. Data are urgently needed to determine and optimize safety of post-pubertal circumcision, and to assess whether preceding pathology may affect HIV acquisition or wound healing.

***Circumcision and risks of STDs and STD symptoms in HIV-negative men***

Circumcision is associated with reduced male risks of viral and bacterial STDs,^24,25,33-38^ genital ulceration,^2,924,37,39^ urinary tract infections^41-43^ and balanitis.^45,46^ In Rakai, GUD is significantly less frequent in circumcised compared with uncircumcised men (RR=0.70, CI 0.54-0.93),^2^ and symptoms of penile inflammation or infection consistent with balanitis are also lower among circumcised men (RR= 0.51, CI0.33-0.77 , unpublished). Circumcision is also associated with reduced risk of penile cancer due to HPV.^44,47^ Thus, the benefits of circumcision may extend to a wide range of STDs and other genital tract infections. This is important because the circumcision effect on HIV may be mediated by protection from STDs.

**1.2 Rationale**

Observational data and three randomized trials show that male circumcision reduces the rate of HIV infection in men by approximately 50-60%. Thus circumcision is an important public health measure because, despite initial costs, it could provide long-term risk reduction in males and secondary protection against infection in their female partners, without incurring recurrent costs. However, the possible efficacy of circumcision for prevention of HIV in women is unclear.

**1.3 Study Design**

We have conducted an individually randomized, unblinded, two armed trial to assess the efficacy of circumcision in HIV-negative men for prevention of HIV and STD acquisition. The study was conducted in rural communities of Rakai District, Ugandan and enrolled 5,000 HIV-negative men who accepted voluntary counseling and testing (VCT). The trial was conducted in two stages which represent a continuum. In the first stage, 200 men were enrolled into a study of the acceptability, feasibility and safety of circumcision, with frequent postoperative follow up to determine rates of wound healing and complications. After assessment of the first 200 men enrolled in stage 1, and in consultation with NIAID Program and the DSMB, we embarked on the main trial (Stage 2) to determine the efficacy of circumcision for HIV prevention. In both stages, we individually randomize consenting HIV-negative men to an early circumcision Intervention Arm or delayed circumcision Control Arm. Follow up is for 24 months. We promised to offer circumcision to the Control Arm participants after completion of two years follow up, if there was evidence of safety at that time. However, we informed control arm participants that the efficacy of circumcision for HIV prevention was not be known until the 5^th^ project year, and that they may elect to wait until completion of the trial before deciding on circumcision.

A complementary but separate Gates Foundation supported trial enrolled HIV+ men and men who decline VCT was conducted concurrently in the same communities as the NIH funded trial.

**2.0 STAGE 2 STUDY OBJECTIVES**

The NIH funded trial enrolled 5,000 HIV- men.

**2.2 Stage 2 Objectives:**

Stage 2 followed Stage 1 and was a two armed individually randomized trial of circumcision versus a control condition, in HIV-negative men (n = 2,400 men enrolled per arm, additional to those enrolled in Stage 1, for a total of 2,500 men per arm). The objectives are:

**Primary Objective:**

1. To determine whether HIV incidence will be significantly different among HIV-negative male participants in the Circumcision Intervention Arm, compared to the Control Arm.

**Secondary Objectives**:

To determine:

1. Whether circumcision is acceptable to men in Rakai.

2. If circumcision is safe among HIV-negative men, with and without preexisting penile pathology (e.g., STDs, balanitis, phimosis).

3. Whether STD symptoms, prevalence and incidence are reduced among male participants in the circumcision intervention arm, compared with the control arm.

4. To assess whether men in the circumcision arm adopt higher risk behaviors compared with men in the control arm (i.e., whether the intervention engenders behavioral disinhibition).

**2.3 Time Line**

The time line is given in Table 1.

**3.0 SELECTION AND ENROLLMENT OF SUBJECTS**

**3.1 Population**

Stage 1 enrolled 200 HIV-negative men, with 100 randomized to immediate circumcision and 100 to delayed circumcision (control). Men were enrolled from two populations:

1. Men currently, enrolled in the ongoing Rakai Community Cohort study (RCCS).

2. Men who are not cohort participants but reside in communities accessible to the Kalisizo field station were also invited to participate and enrolled in the Kalisizo Field Station or satellite facilities (called “hubs”). These men were termed “walk in” participants.

**3.2 Inclusion Criteria:**

Male eligibility criteria for the NIH trial were:

- HIV-negative uncircumcised men who accepted to receive their HIV results

- aged 15 - 49 at screening

- who accepted circumcision and agree to randomization of timing of circumcision,

- were capable of and provided full informed consent,

- had no anatomical abnormality of the penis (e.g., hypospadias, severe phimosis) which would preclude safe circumcision, or which constituted a medical indication for therapeutic circumcision

- had no general medical condition that was a contraindication to surgery or use of local anesthesia

- hemoglobin  8 grams/dL

- intended to stay in Rakai District for at least one year and/or available for follow up

**3.3 Exclusion Criteria**

Men were excluded from the NIH trial if they were:

- HIV- positive

- refused receipt of HIV results

- aged <15 or > 49 at screening

- were circumcised or partially circumcised

- refused to accept randomization

- refusedconsent

- had anatomical abnormalities which were contraindications for circumcision or medical

conditions which required therapeutic circumcision

- had medical conditions which were contraindications for surgery or use of local anesthesia

- hemoglobin < 8 grams/dL which was not rectified by treatment ( section 4.2.c)

- did not intend to remain within Rakai for at least one year

**3.4 Enrolment and Randomization Procedures**

Stage 2 enrollment and follow up are summarized in figures 1 and 2 ( page 45-46).

**3.4.a Community Mobilization**

All target communities were informed of the study and men were invited to participate. The community mobilization messages emphasized:

- That the Rakai Health Sciences Program was conducting a study to assess the acceptability and safety of circumcision, in collaboration with the NIH.

- That there was preliminary evidence from Rakai and other studies that circumcision may reduce the risk of male HIV and STD acquisition, but this had not been proven. Therefore, a randomized trial was needed to assess whether circumcision is truly protective against HIV and STDs.

- That circumcision would be offered free of charge in fully equipped surgical theaters, and would be conducted by trained physicians under local anesthesia, in conditions of careful asepsis. Pain management and careful follow up would be provided.

- Men eligible for the study were HIV- negative , aged 15-49, willing to accept randomization, and willing to be followed up in the home/hub for at least two years post enrollment.

**3.4.b. Screening and Enrollment, Informed Consent**

- Screening and enrollment was conducted by Circumcision Enrollment Teams. Medical Officers supervised enrollment in the field and clinics. Enrollment teams generally consisted of a team supervisor, 4-5 research assistants, a data editor and a laboratory technician.

- Cohort participants were enrolled from ongoing cohort surveys. The non-cohort participants were enrolled at the Kalisizo Field Station or “hubs”.

- All men were offered VCT. Pre-result (i.e. pre-test) counseling was done in groups or individually and also included a video. Receipt of results and post-test counseling was done individually and in private.

- All men were interviewed to ascertain genital symptomatology (GUD, discharge, urethritis/dysuria, balanitis, phimosis and other relevant general medical conditions (Form 01 )..

- Men were examined to confirm that they were uncircumcised, and to detect pathology (infections, phimosis, penile abnormalities and GUD), and samples collected from under the foreskin (PCR and HPV swabs, to be assessed with separate funding.)

- Blood, swabs and urine samples were obtained for HIV and STD testing including serologic syphilis.

HIV and syphilis testing was conducted in real time, and results provided if the participant agreed to receipt of results. Men with serologic syphilis received treatment. Men with serologic syphilis who did not have genital lesions were eligible for enrollment (i.e., latent syphilis). Men with serologic syphilis and concurrent GUD (i.e., primary or secondary syphilis), were treated and enrolled if and when the genital lesions were resolved.

- Treatment was provided for infections and referral provided for penile abnormalities or intractable infections prior to enrollment. STD treatment was made available for partners.

- Finger stick blood was collected for hemoglobin measurement using a portable hemoglobinometers. Men with Hgb < 8.0g/dL were treated for anemia, but not enrolled in the NIH trial unless and until their Hgb  8.0g/dL

- Informed consent for screening was obtained. Men eligible for the study completed another consent document prior to enrollment and randomization (Consent Forms I and Ia).

- After randomization men were informed of their allocation and provided with a post-randomization information sheet describing immediate or delayed circumcision procedures (Form II).

- Men who were initially found ineligible due to treatable genital conditions, anaemia, or men reporting more than 30 days since last screening were re-assessed for eligibility using the eligibility form.

- Men were provided with a circumcision study ID# , as well as a Rakai alphanumeric study ID#. These were preprinted labels attached to questionnaires and samples. In addition, a photo ID allowed tracking and correct identification of subjects.

- Those men randomized to immediate circumcision were scheduled for surgery and given tetanus toxoid immunization. Arrangements were made for transport if needed.

- Men randomized to the control arm were informed that they would be re-contacted for follow up and were offered free circumcision, contingent on Program and DSMB approval, after 24 months

- Eligibility for enrollment into the NIH trial was recorded on the NIH Eligibility verification Form 22.

**3.4.c. Procedures at Enrollment**

- Cohort participants who had completed the baseline survey less than six months prior to enrollment, did not require a baseline re-interview.

- Cohort participants recruited from the ongoing RCCS cohort study who had completed a follow up interview on sociodemographics, risk behaviors and health more than six months before enrollment were administered the baseline survey (Form 02a).

- Non-cohort participants were asked to provide a baseline interview and samples using the current cohort questionnaires (Form 02a).

- All men received soap, and men scheduled for surgery were asked to wash their penis daily until surgery

**3.4.d. Randomization**

- Johns Hopkins University generated a list of randomized numbers in blocks of 20 to insure comparability of men randomized to intervention and control arms, within cohort communities or groups of “walk in” participants”.

- A random assignment sheet was placed in an opaque sealed envelope. The envelopes in blocks of 20, were generated by Rakai health sciences Program Data management using a computerized list provided by JHU, and was retained securely by the Circumcision Data Management Team.

- After enrollment and consent, men were asked to select an envelope from the block of 20 envelopes being used at that time. The envelope contained the assignment sheet showing the randomization number and random assignment to treatment or control arm. The Enrollment Team then affixed the label with the individual’s study ID number to the sheet. (Form 18).

- The assignment sheet with the study ID# labeled was entered into a data base to record study arm allocation.

- The list of random allocation numbers linked to participants was retained at JHU in locked files, and in password protected computers.

- All randomization envelopes were logged out and in daily. A data base monitored used and unused allocation numbers, and track disposition of all envelopes.

- Men were randomized at the enrollment visit. If participants change their minds and withdraw, or at a subsequent preoperative visit, the surgeon determines that circumcision is contraindicated for medical reasons, the men would still be considered enrolled and randomized.
- After randomization, men completed a consent comprehension test to ensure that they understood the trial and to correct any misunderstandings.

**4.0 STUDY TREATMENTS** AND PROCEDURES AFTER CLOSURE OF ENROLLMENT

**4.1. Surgery**

4.1.1 Trial

On the scheduled day of surgery transport was provided if needed. Men who previously consented but changed their minds and were contactable were interviewed to ascertain why they declined surgery. Repeat visits to contact absentees were conducted for a period of approximately one month, and if not contacted, their location and reason for absence were obtained from household members. Men who reported for surgery more than 30 days since last hemoglobin test had a repeat hemoglobin test.

Men who changed their minds: a) if at 6 months a man randomized to circumcision decides not to have surgery, he was classified as a cross-over and followed over two years**. b)** For men who receive delayed surgery between 1-6 months, the subsequent 6, 12 and 24 months visits were scheduled from time of surgery rather than from time of enrollment. For men with surgery delayed for less than 1 month (<1month), subsequent 6, 12, and 24 month follow up occured from time of enrollment.

Men who had a medical contraindication resulting in delayed surgery could come for surgery within 6 months post enrollment. If a man had a contraindication that did not resolved by 6 months, he was **classified as not eligible and withdrawn from the study.**

Men were re-consented for surgery (using the revised Surgical Consent Form III), and if they agreed to surgery they were briefly interviewed and examined by a clinical assistant, and by a physician who would certify fitness for surgery (Preoperative Interview/Exam 03).

Surgery was performed per protocol as indicated below:

**4.1.2 PROCEDURES AFTER CLOSURE OF ENROLLMENT**

All control participants will be informed of the trial findings and will be offered surgery. Control participants will be contacted in the chronological order of their enrollment, so that controls who enrolled earlier and completed 24 months follow up will be given initial priority, and after completion of surgery for these men, priority will then be accorded to men by date of enrollment. Controls who present at the the Kalisizo facility for unscheduled visits and who request surgery will also be provided with circumcision at time of presentation. Incident cases in the control arm who have completed 24 months follow up will be offered surgery, but will have more frequent follow up under a separate Gates funded trial protocol for HIV-infected men. Men initially randomized to the intervention arm who failed to return for surgery within six months of enrollment and were classified as crossovers, will also be offered surgery.

All participants, their spouses and the community at large will be informed of the trial results, and of the availability of surgery for trial participants who remain uncircumcised.

**4.1.a Pre-operative history and examination**

- All participants who have not received tetanus toxoid will be offered immunization.

- The genitalia will be inspected

The following additional samples will be collected: urine and swabs from under the foreskin for future testing, such as gonorrhea/chlamydia/HSV-2 and HPV, with separate funding. During surgery a filter paper blood spot will be collected for archival purposes.

- If there is clinical evidence of current infection (e.g., discharge, GUD), that might increase the risk of surgery, circumcision will be deferred and the patient will be treated and given penile hygiene instructions.
- If there are anatomic abnormalities (e.g., hypospadias, severe phimosis, urinary retention), the patient will be referred for evaluation to the consultant urologist, who will visit the clinic approximately at 2-4 week intervals to review these cases.
- For men who live a long distance from the Kalisizo clinic, a pre-operative overnight stay will be made available, if they choose to use this service.

**4.1.b Surgical Procedure:**

Sleeve circumcision will be performed as follows:

- The penis and both surfaces of the foreskin are preped.

- Local anaesthesia (a mixture of Lignocaine and Marcaine) will be used to block the dorsal penile nerves at the base of the penis and to infiltrate the frenulum.

- With the foreskin in place and the skin lying undistorted on the shaft, a line is drawn following the outlines of the corona and the V of the frenulum. The foreskin is then retracted and a line marked 0.5 - 1 centimeter proximal to the corona, straight across the base of the frenulum.

- With the foreskin retracted the first incision follows the line marked on the inner surface, and bleeding is controlled by bipolar cautery. The incision is deepened to mobilize the skin edge. The prepuce is then extended over the glans and the marked proximal incision is made on the external surface.

- The distal skin edge of the foreskin will be retracted using skin hooks and/or artery or Allis clips, or sutures to permit dissection to free the dartos fascia from the skin.

- The sleeve of prepuce is then removed and will be retained for pathophysiologic studies.

- The foreskin will be laid out flat and measurements taken of the margins for estimates of surface area. The foreskin will then be cut into four sections placed in safe-fix ^TM^ or other appropriate fixative for archiving in Uganda and the US for histopathology.

- All bleeders are controlled,

- Skin edges re-approximated with 4-O or 5-O absorbable sutures. The first stitch is a simple suture at the apex of the frenulum followed by a horizontal mattress suture at the base of the frenulum, and then three vertical mattress sutures of each of the remaining three points of the quadrants, and a minimum of two simple sutures between each principle mattress suture (a total of 13 or more stitches) .

- The wound is cleaned with normal saline

- Medicated, non-adhesive gauze is applied and knotted on the four principle sutures, another free medicated, non-adhesive gauze is wrapped around the first, and a regular gauze dressing applied, followed by an elastic bandage, held in place with strapping

- The procedure usually takes 45-60 minutes.

- The patients will recuperate for approximately 2-4 hours in a post-operative recovery room or until they are ready to go home.

- The surgical procedure and postoperative recovery will be recorded on Form 04 .

-. Men will be given well fitting underwear for comfort and to prevent wound disruption, if needed.

Other methods of circumcision such as forceps or dorsal slit may be used

**4.1.c Postoperative care**

- Analgesics will be provided (see 4.2)

- Men will not be discharged if they experience severe pain, cannot walk independently or have evidence of bleeding through the dressing

- Men will be instructed not to share their pain medications. The postoperative visit will be used to monitor analgesia use.

- Men will rest for a minimum of 30 minutes or longer until they feel able to return home All men will be examined by a physician or health worker prior to discharge and a Form 04 completed.

- Men will be provided with instructions on wound care and analgesia, and this will be reviewed with them before discharge (Postoperative patient Information Sheet 46).

- At time of discharge, men will be offered free transport. Men who decline or do not require transport (residence close to the Rakai Health Sciences Program clinic) will be advised to be accompanied by a family member, friend or Rakai Health Sciences Program staff.

- Free postoperative overnight stay facilities will be made available, if men choose to avail themselves of this service.

**4.1.d Asepsis**

- All theater staff will be trained in aseptic procedures, including documentation.

- Steam Indicators, Biological Indicators, autoclave tracking labels and instrument lists will be used to insure sterility, tracing of instruments and QC.

**4.1.e Emergency procedures:**

- A crash cart, IV and oxygen will be available for emergencies.

- The main potential emergency is excessive bleeding, which will be controlled by ligation of vessels and by pressure. If uncontrolled bleeding persists, a pressure dressing will be applied, and a catheter wrapped around the penile shaft as a tourniquet. If these measures do not control bleeding, an IV will be inserted and oxygen provided if needed, and the patient will be transported to hospital (Kalisizo Hospital < 5 minutes away; larger hospitals in Masaka (Masaka Hospital or Kitovu, a mission hospital ~30 minutes) away, if needed.

- Anaphylactic reaction to the local anaesthetic will be managed with epinephrine, oxygen, IV and transport to the Hospital, if needed.

**4.2 Concomitant Medications**

**4.2.a. Analgesia**

- Experience has shown that postoperative Tramadol is not needed for pain control due to the long action of the local anaesthesia. Also, Tramadol can delay discharge due to drowsiness Therefore postoperative and subsequent analgesia will use Acetominophen 325 to 650 mg, every 4-6 hours, as needed.

Postoperative analgesia with Tramadol hydrochloride 50-100 mg stat will be provided if Acetominophen does not control postoperative pain.

Men will be evaluated within 48 hours, and if moderate to severe pain is reported, Tramadol hydrochloride 50 - 100mg- will be dispensed.

**4.2.b. Treatment of Infections**

*1. Treatment of STDs and Balanitis*.

- we will use symptom-based management of symptomatic infections at time of survey. In addition, syphilis treatment will be provided on the basis of serology.

- The regimen of treatment will, where possible, use directly observed, single oral therapies developed and evaluated in the STD Control Trial. The treatment algorithms include the following:

1) *GUD*. Therapy will cover *T. pallidum, H. ducreyi* and HSV-2: azithromycin 1 gram, ciprofloxacine 500 mg stat, IM benzathine benzylpenicillin 2.4 million units if there is serologic evidence of syphilis, and acycolvir 400mg t.d.s for five days for active herpes (per PDR 2003) ,other therapy may be provided as medically indicated

2) Urethral discharge/dysuria in men. Therapy will cover *N. gonorrohoeae*, and *C. trachomatis*: azithromycin 1 gram, ciprofloxacine 500 mg.

3) *Balanitis* treatment will consist of metrondizaole 2 grams stat for anaerobes, and topical clotrimazole for *C. albicans*.

*2. Pre- or post-operative penile infections* (other than balanitis) will be treated with azithromycin and ciprofloxacin. Indications for treatment are: a) clinical evidence of infection among men presenting for circumcision or after circumcision, and b) symptoms suggestive of current STDs (GUD, discharge, dysuria), reported by men during surveys or at post-circumcision visits, in both arms. Uganda MoH standard of care will be used for partner notification (MoH-STD Form 4) – Form 35 and provision of free treatment.

**4.2.c. Treatment of Anemia**

Men with hemoglobin < 8 grams/dL, will receive iron/folate supplements and presumptive treatment for malaria and helminths using standard MoH regimens for treatment of anemia in adults. Enrollment and randomization will be deferred until the hemoglobin is increased to  8 grams/dL. If the patient does not respond to therapy, they will be referred for investigation of persistent anemia. Anemia resulting from hemoglobinopathies such as sickle cell disease will be referred.

**5.0 CLINICAL AND LABORATORY EVALUATIONS**

**5.1 Screening Evaluations**

**5.1.a. Screening Interview and Exam**

- Consent for screening was obtained (Form I)

- An interview ascertained symptoms of penile pathology and other relevant medical conditions. (Form 01)

- All participants were asked to provide blood for HIV testing and hemoglobin determination (See Lab Methods 5.5.1 and 5.5.3) to assess eligibility prior to enrollment Blood and urine was collected and stored for future STD testing (HSV-2, gonorrhea and chlamydia PCR) with other funds (See Lab Methods 5.5.2 ).

- An exam was performed to verify circumcision status, and to diagnose infection, balanitis, phimosis and anatomical abnormalities (Form 01 ).

- At time of exam, swabs were collected for future assays such as Hybrid Capture and/or PCR for HPV, and for Chlamydia and gonorrhea, and M-PCR for GUD (under separate funds).

**5.1.b.**  **Eligibility**

-Eligibility Screening consisted of willingness to participate, consent to circumcision and to randomization of timing of circumcision, confirmation of uncircumcised status, confirmation of HIV- negative status and acceptance of VCT, and confirmation of Hgb 8.0 g/dL. Thus, eligibility could only be determined after interview, exam and HIV/Hgb test which were recorded in the Eligibility Verification Form 22 .

**5.1.c. Enrollment and Randomization**

- After confirmation of eligibility men were consented for enrollment (Form I.a.), randomized and given a Post-Randomization Information Sheet (Form II) to inform them of their study arm allocation. Random allocation was recorded on Form 18.

**5.1.d. Sociodemographic and Behavioral Interview**

- Cohort participants routinely complete a sociodemographic, behavioral and health follow up interview during annual cohort visits (Form 48.). For cohort participants who completed this interview within six months of enrollment, no re-interview was needed. Cohort participants interviewed more than 6 months prior to enrollment were re-interviewed to up date behavioral information.

- Non-cohort participants were interviewed using the RCCS baseline interview Form 02a

**5.2. Evaluations Related to Surgery**

**5.2.a. Preoperative Assessment**

- Prior to circumcision, men were asked to consent to surgery (Form III), assessed by a clinical health worker or a physician. The clinical health worker took a general medical history, conducted a general systems exam and a genital exam (Form 03 ). The assessment included general medical history and general systems exam and a genital exam (Form 03 ). The clinical health worker or physician examined the penis and ensured that the patient is fit for surgery. Any infection or other abnormality which might be a contraindication for surgery were treated per protocol or by referral, and surgery was rescheduled.

- Swabs were taken for future assays such as PCR, HPV and culture

**5.2.b Surgery**

- The foreskin with the frenulum facing upwards, is placed on a board. The width and length is measured in centimeters and recorded.

- Four separate quadrants with full thickness sections of the foreskin is placed in safe-fix or other appropriate fixative and labeled with the ID#.

- A Surgical Record form (Form 04 ), records the surgery and post-operative status prior to discharge.

**5.3 Post-Surgical Evaluations for Intervention Arm, and Post-Enrollment Evaluations for Control Arm Participants in stage 2**

**5.3.a. Post-operative Evaluations**

The follow up schedule for control surgeries is as follows:

The schedule of postoperative evaluations for stage 2 of the trial is given in Figures 2

1^st^ post operative visit <48 hrs of surgery.

2^nd^ post operative visit at 7± 2 days following surgery.

3^rd^ post operative visit 3-6 weeks following surgery

Unscheduled visits can occur at any time if a participant experiences problems. Participants can contact the project via community counselors who are equipped with cell phones. If wound healing is incomplete at visit 3, men will be followed weekly until wound healing is certified.

These visits, which can take place in the participant’s home or at a convenient central location (“hub”), will record AEs, symptoms, resumption of intercourse, assessment of condom use, examination of the wound and collection of a culture swab if there is evidence of infection, evaluation of wound healing and certification if healing is complete. Blood, urine and penile swabs will not be routinely collected at these postoperative visits, unless needed for patient care.

**5.4.a** **Scheduled follow up - Both arms**

All men were seen 4-8 weeks post-Enrollment. For the intervention arm, this coincided with postoperative visit 3 to certify healing.

All participants are followed up at 6, 12 and 24 months to ascertain study end points, including HIV status (Form 12). At each 6, 12 and 24 month follow up contact, blood is collected for real time HIV and syphilis serology, and urine and penile swab samples are obtained for future STD testing (see 5.5.3)..

**5.4.b. Evaluations at the time of premature discontinuation**

If participants withdraw from the study, this will be recorded on a withdrawal form (Form 14).

**5.5. Laboratory Methods for Evaluations**

**5.5.1.HIV Testing**

**5.5.1.a. Screening**

At screening, all samples were assessed by two separate EIAs, and if both EIAs were negative, the participant was informed that he was HIV-uninfected and enrolled. . If both EIAs were positive the participant was informed that he was HIV infected. Men who failed to return for enrollment were followed up and attempted contacts were documented.

If the EIAs were discordant, HIV status was determined by western blot (WB), and only confirmed WB negative or positive participants were provided with results and

VCT. Indeterminate WB samples were subject to qualitative or quantitative PCR on plasma or serum prior to provision of HIV results. EIA/Western blot could be run in the field laboratory in Kalisizo, and thus results could be provided in a timely manner. Only participants with confirmed negative results were enrolled.

**5.5.1.b Follow up**

At follow up (6, 12 and 24 months) we use the two EIA/WB algorithm as described above. In addition, all seroconverters detected by two EIAs, are confirmed by WB. Indeterminate WBs are subjected to qualitative or quantitative PCR on plasma or serum prior to provision of HIV results. Seroconversions during the first 0-6 month follow up are evaluated, and if need be, the baseline and/or 4 week blood samples are tested by PCR to assess whether participants may have been in the window period at time of enrollment, or are early seroconverters by the 4 weeks follow up. However, the 4 weeks blood sample was not tested in real-time.

**5.5.1.c Rapid tests**

Rapid tests were not routinely used for screening. However, if shortages of EIA kits became a problem, the following procedure was to be used: Two separate and sensitive

rapid tests (such as Determine and Stat-Pak) were to be used for screening. We have shown that the negative predictive value is ~99.8%. Both rapid tests must have been negative for

persons to receive their HIV results and be enrolled. If either rapid test was positive (with a weak or strong band), or equivocal, the sample would be subjected to EIA/WB and no

results would be provided to the participant until the latter tests were completed, as described above. All rapid tests would be confirmed by two EIAs and WB as soon as these

became available.

Tests would be run in batches and technicians blinded. If a participant refused to provide blood during follow up, we requested a urine sample for HIV testing using a urinary EIA and Western blot

5.5.2 **STD Testing.** Serology is performed for syphilis and RPR-positive samples are confirmed by treponemal-specific tests such as TPHA, TPPA, or FTA-ABS.

Using other funding and separate protocols, stored samples will be tested in future for infections such as herpes simplex virus type-2 (HSV-2) infection using Focus EIA. *Neisseria gonorroheae* and *Chlamydia trachomatis* will be detected by PCR on male urine samples Since GUD is a cofactor for HIV acquisition, all ulcers are swabbed for future testing by PCR for syphilis, *H. ducreyi* and HSV-2 in U.S.A or in Uganda once assays are established in our laboratory. (At this time urinary PCR for chlamydia and gonorrhea are available and HIV PCR viral load is being validated). .

5.5.3. **Hemoglobin**

Hemoglobin was measured at screening in order to determine eligibility for enrollment (see eligibility 3.2). Hemoglobin is determined using portable hemoglobinometers (e.g., HemoCue). A finger stick fresh blood sample is collected in a cuvette inserted into the machine and read within 10 minutes. The machines are calibrated each morning using standard controls.

**5.6. Behavioral Research Methods for Evaluations.**

Behavioral studies have already been conducted on circumcision acceptability and perceptions of risk and benefit with other funds. Behavioral research was conducted concurrently with the trial to assess participant and community beliefs and attitudes towards circumcision, to evaluate the experience of men receiving surgery and the attitudes of control subjects allocated to delayed circumcision. These studies provided qualitative information on participant and community beliefs about possible protective effects of circumcision which might affect behaviors, including disinhibition. Separate informed consents were developed for these behavioral study participants. Subjects included male trial participants and non-trial participants, and females (irrespective of whether their partners are trial participants or not). The methods include:

*Structured Interviews* were conducted with male trial participants at enrollment and in follow up surveys to ascertain risk behaviors (number and types of partners, coital frequency, condom use, alcohol use etc.), self-perceived HIV risk and use of VCT.

*Rapid Ethnographic Assessment (REA).* For exploration of norms, values and meanings regarding circumcision, we conducted in-depth interviews and focus group discussions with men and women aged 15-49 . This information will assist in the development of education messages on the benefits and risks of circumcision, and will facilitate interpretation of information obtained by the structured interviews. Application of multiple data collection methods permits “triangulation”, and validation of findings. The domains to be specifically explored include perceptions, facilitators and barriers to circumcision, the relationship of circumcision to sexuality, religion, and risk for HIV/STDs. Behavioral data was collected throughout the study to adjust our education and community motivation programs as needed.

*In-depth interviews* were conducted with male and female key informants from different age groups (15-19, 20-29, 30-39, 40-49) to provide insights into the experiences, attitudes, perceptions and opinions of individuals. A total of approximately 40 informants (~5 male and ~5 female from each of the age groups), were identified through purposive sampling, based on informant’s enculturation and involvement in their respective communities. The sample included representation by residence, ethnicity, religion, economic activities, and education. Open-ended questions werbe used to encourage conversation and enable probing, to elicit information on experiences and on disinhibition. Although females were not enrolled in the trial, we believe it was crucial to enroll them in the REA, since their attitudes and support will be critical for male circumcision acceptance.

*Focus group discussions*. We conducted approximately 32 focus groups each stratified by marital status and gender in age groups 15-19, 20-29, 30-39 and 40-49 to elicit information on shared beliefs, opinions, group concerns and expectations, group knowledge and awareness, and other normative perspectives. The primary objective was to elicit normative perceptions of circumcision, terminology used locally in discussing circumcision, and opinions regarding its acceptability as an HIV/STD preventive measure. Focus group discussions with community gatekeepers, stakeholders as well as community members were held.

*Qualitative data management and analyses:* The ethnographic data was transcribed and translated by experienced Rakai Health Sciences Program personnel. The word processed text was then coded and organized using NVIVO. Further editing was done in Uganda and the US. Data analysis/summaries will be completed in the US and will focus on barriers to circumcision, means for overcoming barriers, incentives that might be acceptable, and perceptions or beliefs which might lead to behavioral disinhibition.

**6.0 DATA COLLECTION, SITE MONITORING AND ADVERSE EXPERIENCE REPORTING**

**6.1 Records to Be Kept.**

The records kept for each subject are consents and record forms. These are summarized in Tables 2. Files are maintained on each participant to provide a chronological record of all forms used. Files are organized by study number and maintained in locked secure storage rooms.

**6.2 Data Management**

- Data management is conducted by the Rakai Health Sciences Program and JHU. We have extensive experience managing large data sets. We use existing Rakai Health Sciences Program census and survey forms, with supplementary record forms specific to the trial. Forms were translated into Luganda and printed in Uganda.

- All participants have a household census enumeration. Census files are used to generate updated data to provide computer generated tracking forms, and pre-printed labels for all questionnaires and samples.

- Data collection is conducted by highly trained and experienced full-time, same-sex interviewers. Study editors check all completed questionnaires and query inconsistencies or errors in the field or hub so that interviewers can return to re-interview subjects and correct errors immediately.

- Subjects are identified by photo IDs and have permanent, unique alphanumeric check digit study ID numbers which prevent keying errors. Subjects enrolled from the cohort also have location numbers for community, household and census resident numbers to facilitate tracing.

- Data entry is done in Rakai Offices using Foxpro screens with programmed range and consistency checks. Lab data and questionnaire are double entered. The data entry system is adapted for this trial by experienced data managers in Uganda and JHU, and relational data bases will be constructed.

- The data is sent by FTP or zip files to JHU. Further data editing and cleaning are conducted at JHU and queries sent to Uganda for clarification and error correction.

- Security is maintained by storing all data files on codeword protected machines accessible only to senior data managers and analysts.

- Standard operating procedures are written and updated.

- Adherence to protocol is assessed by periodic checks on consent forms and hard copy

study instruments, as well as data sets.

**6.3. Clinical Site Monitoring and Record Availability**

Monitoring is conducted by Westat or other monitors at intervals to be determined by NIAID.

All study hard copy records will be available to site monitors and will be maintained in individual chronological files on all study participants. Computer files may be accessed on site during a monitoring visit.

**6.4. Procedures at each study visit.**

| Procedure/Tests | Screening/  Enrolment | Post-operative, Intervention | | 4 weeks | 6 months | 12 months | 24 months |
| --- | --- | --- | --- | --- | --- | --- | --- |
|  |  | < 48 hrs | 5-9 days |  |  |  |  |
| Sample collection | X |  |  | X | X | X | X |
| Blood | X |  |  | X | X | X | X |
| Urine | X |  |  |  | X | X | X |
| Swabs | X |  |  |  | X | X | X |
| GUD swabs | X |  |  | X | X | X | X |
| Tests in real time |  |  |  |  |  |  |  |
| Hgb | X |  |  |  |  |  |  |
| HIV | X |  |  |  | X | X | X |
| Syphilis | X |  |  |  | X | X | X |
| Exam | X | X | X | X | X | X | X |
| VCT | X |  |  |  | X | X | X |

**6.4. Serious Adverse Event Reporting**

The occurrence of severe adverse events (SAEs) is recorded at every post-randomization visit.

6.4a General Definitions

An AE is defined, as “any untoward medical occurrence in a participant and that does not necessarily have a causal relationship with the treatment. An AE can be any unfavorable and unintended sign (including an abnormal laboratory finding), symptom or disease temporarily associated with the beginning of the treatment (i.e., following randomization).**”** Adverse events during screening and prior to randomization that result from study participation were also reported.

- The adverse events are defined in Table 3 and AE notification is given in form 11a.

**6.4.b Severity of AEs**

- The severity of AEs is characterized as:

. *mild* (transient and not affecting activity),

*. moderate* (requiring modification of activity or additional medical/minor surgical/intervention),

*. severe* (resulting in: death, life threatening condition, incapacitating symptoms which require hospitalization, significant medical/surgical interventions or which result in persistent significant disability/incapacity).

- All severe occurrences are considered serious adverse events (SAEs) for reporting purposes, consistent with FDA definitions as follows:

. Life threatening: substantial risk of dying at the time of the AE

. Hospitalization (initial or prolonged): if admission to hospital or prolongation of hospital stay results from the AE.

. Disability: if the AE resulted in a significant, persistent or permanent change, impairment, damage or disruption in the patient’s body function/structure, physical activities or quality of life.

. Intervention required to prevent permanent impairment or damage: if it is suspected that the treatment may result in a condition which required medical or surgical intervention to preclude permanent impairment or damage to the patient.

**6.4.c Relationship of AEs to the intervention**

The relationship of AEs to study participation, particularly surgery or medications will in most cases be clear. We use the following classification.

. *definitely related* to study procedures such as surgery or anesthesia/analgesia (occurred during surgery or postoperatively),

. *probably related* (occurred during or after study procedures such as surgery, most likely to be due to the study procedure but could arise from other causes,

. *possibly related* (occurred during participation, but could equally or more plausibly be ascribed to other causes), and

. *unrelated* (an event clearly explained by causes other than the trial participation

- The timing of AEs is divided into screening/enrolment/randomization, operative, postoperative and during longer-term follow up.

**6.4.d Study Definition of Adverse Events**

The anticipated adverse events are largely those associated with surgery or study medications. The description of anticipated potential AEs is given in Table 3. (This is in part based on the AE classification used for the Kisumu trial of male circumcision). All medical officers were trained in the recognition and classification of the adverse events, and in the procedures required for reporting. The senior Medical Officers, will be responsible for AE reports.

Any other serious events not defined in Table 3 will be reported as SAEs.

**Table 3. Study Definition of Adverse Events**

| Adverse Event Type | Description of Adverse Event Type | Severity  and Codes |
| --- | --- | --- |

|  |  |  |
| --- | --- | --- |

| A. During Surgery |  |  |
| --- | --- | --- |

| Pain | Minor, not requiring additional anaesthesia | Mild (APS1) |
| --- | --- | --- |

|  | Moderate/severe controlled with additional anaesthesia* | Moderate (APS2) |
| --- | --- | --- |

|  | Severe, not controlled by additional anaesthesia | Severe (APS3) |
| --- | --- | --- |

| Excessive bleeding | More bleeding than usual, but easily controlled | Mild (ABL1) |
| --- | --- | --- |

|  | Bleeding that requires pressure dressing to control | Moderate (ABL2) |
| --- | --- | --- |

|  | Blood transfusion or transfer to another facility for management required | Severe (ABL3) |
| --- | --- | --- |

| Anesthetic-related event | Palpitations, vaso-vagal reaction or emesis | Mild (AAN1) |
| --- | --- | --- |

|  | Reaction to anesthetic requiring medical treatment in study clinic but not transfer to another facility | Moderate (AAN2) |
| --- | --- | --- |

|  | Anaphylaxis or any reaction requiring transfer to another facility | Severe (AAN3) |
| --- | --- | --- |

| Excessive skin removed | Adds time or material needs to the procedure, but does not result in any discernable adverse condition | Mild (AES1) |
| --- | --- | --- |
|  | Skin is tight, but additional operative work not necessary | Moderate (AES2) |

|  | Requires re-operation or transfer to another facility to correct the problem | Severe (AES3) |
| --- | --- | --- |

| Damage to the penis | Mild bruising or abrasion, not requiring treatment | Mild (ADP1) |
| --- | --- | --- |

|  | Bruise or abrasion to the glans or shaft of the penis requiring pressure dressing or additional surgery to control | Moderate (ADP2) |
| --- | --- | --- |

|  | Portion or all of the glans or shaft of the penis severed or burned by electrocautery | Severe (ADP3) |
| --- | --- | --- |

| B. First Month Post-Surgery |  |  |
| --- | --- | --- |

| Pain | Symptoms of pain requiring bed rest for less than half the day | Mild (BPA1) |
| --- | --- | --- |

|  | Pain requiring bed rest for more than half day | Moderate (BPA2) |
| --- | --- | --- |

|  | Excruciating pain requiring total bed rest | Severe |
| --- | --- | --- |

| Excessive bleeding | Dressing or other materials (for example underwear) spotted but dry | Mild (BBA1) |
| --- | --- | --- |

|  | Dressing or other materials wet with clotted blood requiring change of dressing | Moderate (BBA2) |
| --- | --- | --- |

|  | Dressing or other materials soaked with blood with obvious active bleeding requiring surgical exploration or transfusion | Severe (BBA3) |
| --- | --- | --- |

| Excessive skin removed | Client concerned but no discomfort on erection | Mild (BES1) |
| --- | --- | --- |

|  | Causes slight disconfort on erection but surgical correction not necessary. | Moderate (BES2) |
| --- | --- | --- |

|  | Interferes with life and surgical correction necessary | Severe (BES3) |
| --- | --- | --- |

| Insufficient skin removed | Prepuce partially covers the glans only when extended | Mild (BIS1) |
| --- | --- | --- |

|  | Prepuce still partially covers the glans and re-operation is required to correct | Moderate (BIS2) |
| --- | --- | --- |

|  | Not applicable |  |
| --- | --- | --- |

|  |  |  |
| --- | --- | --- |

| Swelling/Hematoma | More swelling than usual, but no treatment needed | Mild (BSH1) |
| --- | --- | --- |

|  | swelling requiring surgical exploration but no evidence of active bleeding | Moderate (BSH2) |
| --- | --- | --- |

|  | Rapidly expanding Heamatoma suggesting active bleeding requiring surgical exploration or referral | Severe (BSH3) |
| --- | --- | --- |

| Damage to the penis | Mild bruising or abrasion, not requiring treatment | Mild (BDP1) |
| --- | --- | --- |

|  | Bruise or abrasion to the glans or shaft of the penis requiring pressure dressing or additional surgery to control | Moderate (BDP2) |
| --- | --- | --- |

|  | Portion or all of the glans or shaft of the penis severed | Severe (BDP3) |
| --- | --- | --- |

| Infection | Pain and erythema with no obvious swelling | Mild (BIN1) |
| --- | --- | --- |

|  | Painful swelling with erythema or elevated temperature or purulent wound discharge | Moderate (BIN2) |
| --- | --- | --- |

|  | Cellulitis or wound necrosis | Severe (BIN3) |
| --- | --- | --- |

| Delayed wound healing | Healing takes longer than usual, but no extra treatment necessary | Mild (BDW1) |
| --- | --- | --- |

|  | Additional non-operative treatment required | Moderate (BDW2) |
| --- | --- | --- |

|  | Requires re-operation to correct | Severe (BDW3) |
| --- | --- | --- |

| Appearance | Client concerned, but no discernable deformity | Mild (BAP1) |
| --- | --- | --- |

|  | Minimal deformity does not require re-operation | Moderate (BAP2) |
| --- | --- | --- |

|  | Significant deformity requires re-operation to correct | Severe (BAP3) |
| --- | --- | --- |

| Problems with voiding | Transient complaint that resolves without treatment | Mild (BVO1) |
| --- | --- | --- |

|  | Requires a special return to the clinic, but no additional treatment required | Moderate (BVO2) |
| --- | --- | --- |

|  | Requires referral to another facility for management | Severe (BVO3) |
| --- | --- | --- |

| Wound dehiscence | Wound disruption involving no more than one principal suture. | Mild (BWD1) |
| --- | --- | --- |
|  | Wound disruption and involving two or more principal sutures. No surgical intervention required. | Modarete (BWD2) |
|  | Significant wound disruption requiring surgical correction | Severe (BWD3) |
|  |  |  |

| **C. One Month or More Post-Surgery** |  |  |
| --- | --- | --- |

| Infection** | Pain and erythema no obvious swelling | Mild (CIN1) |
| --- | --- | --- |

|  | Painful swelling/purulent wound discharge | Moderate (CIN2) |
| --- | --- | --- |

|  | Cellulitis or wound necrosis | Severe (CIN3) |
| --- | --- | --- |

| Delayed wound healing | Healing takes longer than usual, but no extra treatment necessary | Mild (CDW1) |
| --- | --- | --- |

|  | Additional non-operative treatment required | Moderate (CDW2) |
| --- | --- | --- |

|  | Requires re-operation to correct | Severe(CDW3) |
| --- | --- | --- |

| Appearance | Client concerned, but no discernable deformity | Mild (CAP1) |
| --- | --- | --- |

|  | Significant scarring or other cosmetic problem, but does not require re-operation | Moderate (CAP2) |
| --- | --- | --- |

|  | Requires re-operation to correct | Severe (CAP3) |
| --- | --- | --- |

| Excessive skin removed | Client concerned, but there is no deformity on erection | Mild (CES1) |
| --- | --- | --- |

|  | Causes slight discomfort on erection but surgical correction not necessary | Moderate (CES2) |
| --- | --- | --- |

|  | Interferes with sexual life and surgical correction is necessary | Severe (CES3) |
| --- | --- | --- |

| Insufficient skin removed | Prepuce partially covers the glans only when extended | Mild (CIS1) |
| --- | --- | --- |

|  | Prepuce still partially covers the glans and re-operation is required to correct | Moderate (CIS2) |
| --- | --- | --- |

|  | Not applicable |  |
| --- | --- | --- |

|  |  |  |
| --- | --- | --- |

|  |  |  |
| --- | --- | --- |

| Torsion of penis | Torsion is observable, but does not cause pain or discomfort. | Mild (CTP1) |
| --- | --- | --- |

|  | Causes mild pain or discomfort on erection, but additional operative work not necessary | Moderate (CTP2) |
| --- | --- | --- |

|  | Requires re-operation or transfer to another facility to correct the problem | Severe (CTP3) |
| --- | --- | --- |

|  |  |  |
| --- | --- | --- |

| Erectile dysfunction | Client reports occasional inability to have an erection | Mild (CED1) |
| --- | --- | --- |

|  | Client reports frequent inability to have an erection | Moderate (CED2) |
| --- | --- | --- |

|  | Client reports complete or near complete inability to have erections | Severe (CED3) |
| --- | --- | --- |

| Psycho-behavioral problems | Client reports mild sexual dissatisfaction attributed to circumcision, but no significant psycho-behavioural consequences | Mild (CPB1) |
| --- | --- | --- |

|  | Client reports significant sexual dissatisfaction attributed to circumcision, but no significant psycho-behavioural consequences | Moderate (CPB2) |
| --- | --- | --- |

|  | Significant depression or other psychological problems attributed by the participant to the circumcision | Severe (CPB3) |
| --- | --- | --- |
| Other AES | Other AEs are described below*** |  |

**COMMENTS:**

* Additional anesthesia implies a volume of local anesthetic in excess of initial amount drawn up in the syringe and calculated to be sufficient for patient’s weight before surgery.

** Infection is indicated by swelling, erythema, pain, elevated temperature ( locally or systemically, or purulent discharge)

*** **Other AEs**

**New AE codes**

During the February 2006 review, it was decided that specificity could be improved by addition of new codes. This did not affect the diagnosis of AEs, but added more detail for future analyses. The additional codes were:

1. A new code for joint dehiscence (BWD) and infection (BIN) AEs, indicated by BWD/BIN. Degree of severity is per individual event and the first event to occur is reported first.

2. New codes for stich sinus: SS = Sinus at Suture site SS1 = Sinus with non-puss discharge SS2 = Sinus with purulent discharge. This would be included in infectious AEs, but adds specificity.

3. New code for anatomical site of lesion (F = Frenulum), because many wound dehiscence, poor skin apposition and infection involved the frenulum.

4. New codes for pain on intercourse: PI = Pain on Intercourse PI1 = No interference with sex, PI2 = Reduced sexual frequency, PI3 = Refrain from intercourse due to pain

5. A new code was added for allergic reaction to the iodine prep, since we observed cases of blisters following surgical site preparation

PREP REACTION APR 1 = MILD BLISTERS RESOLVED

APR 2 = MORE SEVERE BLISTERS REQUIRING TREATMENT

6. A number of cases of pain or wound dehiscence occurred after intercourse, so we coded intercourse related AEs: I = RELATIONSHIP TO INTERCOURSE

7. A number of cases of wound dehiscence were due to external trauma (e.g., heavy lifting, bicycle accidents) so this was coded as EX = EXTERNAL CAUSE

8. Code for stitch granuloma: SG=Stitch granuloma. CSG 1=Small capable of resolving,no need for surgical removal. CSG 2=Not capable of resolving, requires surgical intervention. CSG 3=Not applicable

No changes were made to the AE definitions, although with hindsight, we believe these could be improved for monitoring of control surgery related AEs.

All other SAEs resulting in hospitalization or death are reported to DAIDS and the IRBs of record in real time (i.e., within 10 days of PI notification of the event).

Genitourinary AEs other than those listed in the above tables are reported as cumulative aggregate events to the DSMB and IRBs at annual intervals. “Other AEs” that are not included in the above list and do not affect the genitourinary system, are reported to the DSMB in annual aggregate tabulations, for circumcised men during the first 90 days following surgery. These “other AEs” are also reported for controls within 90 days of enrollment. The Medical Officer will be informed of “other AEs” within 10 days of notification.

The “other AEs” reportable with in 90 days of enrollment will include:

1) Liver or pancreatic abnormalities

2) Neurologic conditions of the CNS (e.g., meningitis, encephalitis, convulsions and headaches, visual and auditory disturbances, strokes), and peripheral neurologic conditions (e.g., peripheral neuropathies, motor weakness etc.).

3) Myocarditis and pericarditis

4) Dermatologic condition affecting the genital, pubic, perianal or groin

5) Injuries and accidents

Common illnesses unrelated to circumcision will be excluded from the “other AE” category to avoid unnecessary reporting of irrelevant events. The excluded AEs are as follows:

1. Malaria and other common parasitic infections.
2. Gastrointestinal tract (GIT) diseases, including diarrhea, gastroenteritis (bacterial, viral or parasitic), and other GIT conditions, and oropharangeal infections
3. Respiratory illnesses (URTI, ALRI, pleurisy), including TB, influenza or other respiratory infections.
4. Angina, myocardial infarction, congestive cardiac failure,
5. Dermatologic conditions (e.g., scabies, infected and uninfected rashes, pruritis, fungal infection), except those affecting the genital, pubic, perianal or groin
6. Neoplasms not affecting the genitourinary tract
7. Surgical conditions not related to the intervention (eg., hernias) and which do not require hospitalization

**6.4.e Management of AEs**

- Free medical care is provided for AEs directly related to participation including surgical care if needed. If referral for medical or surgical complications is required, participants are referred to the Kalisizo Hospital (2-3 minutes driving time) from the surgical facility the Rakai Hospital or hospitals in the neighboring District of Masaka (approximately 30 minutes driving time) from the surgical facility**.**

**6.4.f Reporting of AEs**

- All AEs are ascertained and documented by Project medical officers

- All deaths and grade 3 or higher SAEs are notified to the Ugandan and U.S. PIs within 48 hours of detection, by e-mail or fax.

- The U.S. PI then notifies the relevant IRBs (Johns Hopkins, Columbia University and Uganda Virus research Institute) and DAIDS Medical Officer in writing as soon as possible, and not later than 10 days after identification of the SAE. The nature, severity, timing and relationship of the AE to circumcision or study medications is specified by the medical officer. A summary of how the severity and relationship to study participation was determined is provided by the PI, and clarifications made by phone with the medical officer as needed.

**.**

**6.4.g Tabulations of AEs**

- The PI submits aggregate reports of all AEs and tabulations to the DSMB. AEs are described by type, time since surgery or medications, severity and relationship to study procedures.

Dates of AEs are also tabulated to assess temporal trends or patterns.

- AEs are tabulated by total number, and by number of AEs per individual for those participants experiencing more than one AE.

**6.4.h Source documentation**

**-** Operative and postoperative AEs use Rakai Health Sciences Program documentation (Forms 11 )

- Medical visits to health centers or other facilities outside of the Rakai Health Sciences Program cannot be completely documented due to poor records. We will endeavor to obtain copies of hospital medical records, after obtaining permission from the index person. Rakai Health Sciences Program reports (such as questionnaires), are also used to provide information.

- To maximize documentation, Rakai Health Sciences Program offesr free medical care or referral for injury or illnesses as a result of study participation. Phone numbers of medical officers are provided on the Postoperative patient Information Sheet (Form 46 ), we provide transport if needed and reimburse participants for transport costs. In addition, we arrange community health workers to provide a contact person in each village who can facilitate contact with the Project. Records are maintained for unscheduled visits (Form 16), and an AE report completed if indicated**.**

**7.0 STATISTICAL CONSIDERATIONS**

As noted previously this is an individually randomized, un-blinded trial of male circumcision to be conducted in two stages. The sample size estimates, by study stage are provided for each study endpoint, end points are defined, and an analysis plan is presented.

**7.2. Stage 2 Sample Size for Study Endpoints**

**7.2.a. Primary Endpoint: Male HIV Incidence.**

***Efficacy:***

The Rakai STD Control Study^78^ found an incidence of 1.8/100 PY among the uncircumcised men and 0.9/100 PY among the circumcised men, with a Poisson adjusted RR = 0.53.^1^ We base our sample size estimates on this efficacy, and use a two-sided test with  = 0.05 and 1- = 0.80.

***Assumptions:***

We assume two years follow up, with participants enrolled between 9-26 months of the study and follow up completed by study month 50, and an annual loss to follow up of 15% (please see feasibility). We also estimate the loss of power due to possible contamination (i.e., cross-over) of 7.5-10%.

***Allowance for contamination or reduced magnitude of anticipated efficacy:***

We previously observed a RR = 0.53 for HIV incidence in circumcised relative to uncircumcised men, and have powered the study to be able to detect a lower efficacy of RR = 0.57. We have also assessed potential effects of contamination as follows: Let RR* be the observed rate ratio in the presence of cross-over (p), and let RR be the true rate ratio in the absence of contamination. Then RR* = (p + [1-p] x RR)/(p x RR + [1-p]) = RR/([p x RR] + [1-p]) (J. Hughes , pc). With p = 0.075 (i.e., 7.5% contamination), the observed RR* = 0.55. The sample size needs to be increased to account for this cross-over effect. With 2,500 men enrolled per arm we would accumulate 2,125 py in the 1^st^ follow up year and 1,806 py in the 2^nd^ follow up year, with a total over two years follow up = 3,932 py, which could detect a RR* ~ 0.57 with 80% power if the true RR = 0.53. (Please note this is 18.4% larger than the 3,209 py needed in the absence of contamination). Therefore, we will enroll 2,500 men per arm providing 3,932 py per arm.

***Allowance for data dependent stopping:***

We propose using formal interim analysis methods for monitoring the primary outcome (incidence of HIV infection) as well as the safety aspect of the study. Lan-DeMets group sequential methods with O'Brien-Fleming bounds ^117,118^ will be utilized to ensure the maintenance of an overall Type I error rate of 5%. For monitoring HIV incidence, we anticipate one interim analysis to allow DSMB to decide whether we can commence circumcisions in the control arm, and allow sufficient time for completion of control surgeries before the end of the project. Assuming an interim analysis at project month 40, we will accrue a total of 3,028 py observation per arm, which represents 77% (3028 * 2 / 3932 * 2) of the information to be accumulated during the trial. This information fraction, for example, will give sequential O'Brien-Fleming boundaries equal to 2.303 for the interim analysis and 2.017 for the final analysis. Without using these boundaries, we estimate (via simulation with 10,000 runs) that the overall Type I error rate wouldl be inflated to approximately 7.7%. Using these boundaries, we estimate that the Type I error rate is maintained at 0.05 and that the power for a RR of 0.53 is equal to 89.5%. Furthermore, with this RR, we estimate that there is approximately a 70% chance of crossing the boundary at the interim analysis. Sequential confidence intervals ^118a^ will be used when reporting results. The investigators will be blinded for the interim analysis. Monitoring of safety will be done similarly, except that it will occur on a continuous basis, and will use the methods of Lan *et al.*

***Age criteria and selection of high risk groups:***

Rakai has is a mature generalized epidemic setting with relatively high incidence throughout the population. Table 5 shows male age-specific HIV incidence. Incidence is lowest among adolescents, high and sustained among men aged 20-29, and declines modestly at older ages. Exclusion of adolescent men 15-19, would exclude a group with considerable HIV incidence and which is of great importance in future programs. Restricting enrollment to the highest risk age group (20-29 years) would marginally increase incidence relative to the total male population 15-49, but would only include 39.5% of all men. Enrollment restricted to men aged 20-29 would require adding many more communities in order to maintain power, but this complicates logistics and inflates costs. Moreover, acceptability was highest (65% in ages 20-29), so self-selective acceptability will automatically over sample these highest risk age groups. We therefore propose to enroll all consenting men aged 15-49.

**7.2.b. Male STDs and STD Symptoms:**

***STDs:***

All participants will be tested for HIV and syphilis at enrollment, 6, 12 and 24 months.. For budgetary reasons, additional specimens will be collected for selective STD testing with other funds. (Please see laboratory methods). From prior data the expected infection rates are: syphilis prevalence ~10% and incidence 2.2/100 py. The RR for syphilis in circumcised versus uncircumcised men was 0.71. With 2,500 HIV- men we expect 2,125 and 1,806 per arm, observed at the 1^st^ and 2^nd^ follow ups, respectively (a total of 3,931 observations per arm). We have >80% power to detect a RR of 0.70 for the effects of circumcision on syphilis prevalence, and >80% to detect a RR = 0.5 in syphilis incidence.

***STD symptoms:***

The RR for GUD in HIV- circumcised versus uncircumcised men was 0.70. With 2,500 men enrolled per arm and 85% follow up, we have 85% power to detect a difference of this magnitude. The RR of balanitis in all circumcised versus uncircumcised men was 0.53 (prevalence 11.6% in circumcised and 22.8% in uncircumcised), and we have 98% power to detect an effect of this magnitude. Therefore, these sample sizes are adequate, even with cross-over effects.

Thus, the study is adequately powered to detect reductions in STDs and STD symptoms in the circumcision relative to the control arm**.**

**7.2.c. Safety of Circumcision**

The frequency of complications following adult circumcision in the Rakai trials is approximately 3.6%. However, surgical and postoperative complications were expected to be ~2%, and were monitored continuously to insure safety. Monitoring of the rate of complications associated with circumcision is done using similar techniques described for monitoring efficacy, except that analysis will occur on a continuous basis. To facilitate the proper analysis of this continuous safety data, we propose to use the methods of Lan, Rosenberger, and Lachin in which they demonstrate how to construct a sequential boundary (Wald boundary) for a Z-statistic used to continuously monitoring data that maintains an overall Type I error rate. The methods build upon the spending-function methods of Lan & DeMets, and can also be used when switching from occasional (e.g. group sequential) monitoring to continuous monitoring.

We proposed to conduct interim analyses if required for DSMB safety assessment at 20 and 32 months. We expected to have completed 1,250 circumcisions by 20 months and 2,500 by 32 months. Using StatXact (Cytel Software Corp), we estimated the smallest number of AEs that would give an exact 1-sided 95% lower confidence bound on the estimated rates of AEs (not adjusted for interim analyses). With 1,250 men circumcised by 20 months, if the total AE rate  2.64% (33 events), the lower 95% CI bound would significantly exceed the expected rate of 2.0% (25 events), and for moderate/serious AEs, a lower 95% CI bound of an observed rate of 0.88% (11 events) would significantly exceed the expected rate of 0.5% (7 events). Similarly, at 32 months with 2,500 surgeries completed, if the total AE rate  2.48% (62 events), the lower 95% CI would exceed the expected 2.0% rate (50 events), and for moderate/serious AEs, with an observed rate of 0.72% (20 events), the lower 95% CI would exceed the expected rate of 0.5% (13 events).

**7.2.d. Behavioral disinhibition:**

From prior data we expect ~ 25% of men to have multiple sex partners, ~ 17.0% to use condoms inconsistently, ~ 5.0% to consistently use condoms, ~ 46.0% report alcohol consumption in the previous 7 days and ~42% report alcohol consumption with sex. These behaviors have been shown to be significantly associated with HIV risk in the Rakai population. Assuming these rates pertain in the control arm, we will have 80% power to detect the following markers of behavioral disinhibition in the intervention arm: a) an increase in multiple sex partners to 30%, b) a reduction of inconsistent condom use to 13.2%, and c) a reduction in consistent condom use to 3% (i.e., an overall reduction in condom use of - 5.8%), and d) and an increase in alcohol use within the past 7 days to 50.4%, or of alcohol consumption with sex to 46.4%.

The relevance of changes in behaviors of this magnitude are discussed in 7.5.b.

**7.3. Accrual and Feasibility:**

The average number of men aged 15-49 per community is ~ 190, of whom we expected 137 to be uncircumcised and HIV-negative (84% uncircumcised and 86% HIV-).^2^ Assuming 50% acceptance of circumcision we expected an average of 69 subjects enrolled per community. With the required sample size of 2,500 men per arm we could enroll this many participants by surveillance in ~36 communities per arm, for a total of ~72 communities. It is feasible to expand coverage to additional communities. However, we would also allow non-cohort participants to enroll as “walk in subjects”. We believed that the current cohort surveillance participants and walk in participants would readily achieve the sample size goals. (Our previous trials enrolled over 12,000 subjects).

We estimated accrual of ~ 150-160 subjects per month. The accrual in relation to timing of the three main DSMB meetings is: ~ 2870 subjects by month 9; ~ 5000 subjects by month ~ 31 (with two year follow up of the 200 Stage 1 participants completed); and 5000 subjects by month 41 (with two year follow up of ~ 2069 volunteers completed).

***Feasibility of follow up:***

In the present trial, the eligibility criteria included intent to remain in the community for at least one year. We implemented additional procedures for active tracing. In brief, we a) asked relatives of absent subjects to contact them or to provide contact addresses, b) asked absentees to return for follow up and compensate them for time and travel expenses, and c) established peripheral hubs in urban centers such as Masaka and Kampala where most absentees go for work or education, in order to facilitate follow up outside the District. With eligibility based on intent to stay and active tracing, we believed that we could achieve a minimum of 85% or more follow up. In our maternal-infant trial we achieved 94% follow up of 4,036 persons, and in our MER study we have achieved 95.4% follow up on a sample of 383 subjects. We therefore believde follow up of 85% was feasible in the proposed trial.

**7.4 Randomization and Stratification**

**-** Johns Hopkins University generated a list of randomized numbers in blocks of 20 to insure comparability of men randomized to intervention and control arms, within cohort communities or groups of “walk in” participants”.

- A random assignment sheet was placed in an opaque sealed envelope. The envelopes in blocks of 20, were generated by Rakai Health Sciences Program Data management using a computerized list provided by JHU, and were retained securely by the Circumcision Data Management Team.

- After enrollment and consent, men were asked to select an envelope from the block of 20 envelopes being used at that time. The envelope contained the assignment sheet showing the randomization number and random assignment to treatment or control arm. The Enrollment Team then affixed the label with the individual’s study ID number to the sheet. (Form 18 ).

- The assignment sheet with the study ID# labeled was entered into a data base to record study arm allocation.

- The list of random allocation numbers linked to participants were retained at JHU in locked files, and in password protected computers.

- All randomization envelopes were logged out and in daily. A data base monitored used and unused allocation numbers, and track disposition of all envelopes.

- Men were randomized at the enrollment visit. If participants changde their minds and withdraw, or at a subsequent preoperative visit, the surgeon determined that circumcision is contraindicated for medical reasons, the men wer still considered enrolled and randomized.

**7.5 Analysis Plan**

**7.5.a Stage 2 Analysis Plan**

***Primary Endpoint, HIV incidence.***

HIV incidence per 100 person-years (PY) will be estimated, assuming seroconversion occurred at the mid-point of each follow up interval. In both arms, time from enrollment will be accumulated up to 24 months of follow up. Person time will discount the postoperative period of sexual abstinence for intervention arm subjects. Baseline samples of subjects who seroconvert during the first follow up interval will be tested by detuned EIA and PCR to detect prevalent infections during the window period, and any suspected prevalent cases will be excluded from incidence analyses.

Exploratory data analysis will examine the populations in the two arms at enrollment to assess baseline comparability. HIV incidence will be determined by strata of covariates such as age, marital status, behaviors (e.g., number of partners, condom use), health status and other variables to identify potential confounders. We will also assess contamination (i.e., cross-over) among men allocated to circumcision who do not receive surgery, and among control subjects who opt to have circumcisions performed from non-Project services.

An intent-to-treat approach will be taken, and all men who enroll will be included, irrespective of whether intervention arm participants ultimately receive circumcision, or whether control arm participants crossover and receive circumcision prior to 24 months follow up. The rationale is that a proportion of enrolled men who initially accept circumcision may change their mind prior to receipt of surgery, and such men are likely to be self-selected. Because circumcision is delayed in the control arm, such self-selection could be differential between randomization groups. Therefore, in the circumcision arm, enrolled men who subsequently refuse circumcision, will be ascribed half the person time from enrollment to scheduled date of circumcision.

We will assess HIV incidence in enrolled HIV-negative men. The primary analytical model that will be used will be Poisson regression to compare the HIV incidence rates between the two study arms.^113^ The relative incidence of HIV in the circumcision arm versus the control arm, will be estimated using Poisson regression models with adjustment for individual-level variables found to differ between groups at enrollment, and for potential confounding variables. Potential confounders determined from prior risk analyses will include age, marital status, risk behaviors (i.e, number and nature of partners, condom use and alcohol use), or imbalance of covariates between study arms will be used for adjustment in the primary models. Secondary analyses will include any additional potential confounders identified during exploratory data analysis and will include covariates associated with HIV incidence (p < 0.15), or covariates with an incidence RR > 2.0. Poisson multivariate models will be fit for the whole population and for strata of particular interest (e.g., by age, sexual risk behaviors etc.). Although we are doing an individually randomized trial, the structure and logistics of field work requires that men be randomized within communities. Thus, we will also fit Poisson models with random effect terms for each community, to insure adjustment for clustering in HIV incidence.

We will also assess the associations between selected STDs and STD symptoms and incident HIV infection to determine whether effects of circumcision on HIV incidence may be mediated by STD cofactors. If, as expected from prior analyses, selected STDs and STD symptoms such as GUD are associated with incident HIV, we will assess the circumcision effects on HIV incidence using stratified **models or via interaction terms.**

***Secondary Endpoints:***

***Male STD Prevalence and GUD.***

Prevalent syphilis and STD symptoms (GUD, discharge/dysuria) at baseline will be compared between study arms. At 6, 12 and 24 months follow up visits we will determine the period prevalence of STD symptoms and cumulative prevalence of syphilis (the latter based on prevalence because of persistence in seropositivity). In persons free of initial serologic infection at baseline, incident syphilis will be derived, and covariate adjusted rate ratios of incident STD symptom endpoints will be estimated by Poisson regression modeling as described above.

***Behavioral change (disinhibition).***

To assess possible behavioral disinhibition we will determine the frequency and changes in reported frequency of risk behaviors (e.g., number, type of partners, condom use, alcohol use) by study Arm, at enrollment and at each follow up. Data will be displayed graphically for each follow up visit. We will estimate the adjusted odds ratios of risk behaviors in the intervention versus the control arms using multivariate logistic regression for each survey visit. We will assess intra-individual changes in risk behaviors as follows. For continuous variables (e.g., number of partners), the intra-individual change in risk behaviors from enrollment will be determined, and the mean within-individual behavioral change between study arms will be assessed using repeated measures ANOVA or paired t tests. For dichotomous variables, we will assess the change in risk behavior over time, and estimate adjusted odds ratios of risk behavioral change between study arms by logistic multivariate regression. For categorical behavioral outcomes (e.g., non use of condoms, inconsistent and consistent use) we will use multivariate polychotomous logistic regression. In addition, we will have data from ongoing cohort surveillance to measure behavioral trends among non-participants, and we will determine behavioral change in the non-trial population in order to assess general trends, and to compare such non-trial associated behaviors with those reported by participants in both arms. A composite risk score will be developed on the basis of these analyses.

The relevance of changes in risk behaviors with respect to HIV incidence will be determined from prior Rakai studies of risk factors for HIV acquisition and from epidemiologic data generated by the circumcision trial. The behaviors specified above have been shown to be associated with a risk (rate ratio) ~ 2.0 or more in our observational analyses. We will estimate the effect on HIV incidence by study arm, of observed behavioral disinhibition (should it occur), using stratified analyses and multivariate analyses and stochastic modeling.

**7.6 Interim Data and Safety Monitoring**

The DSMB halted the trial for efficacy on December 12, 2006 after an unscheduled interim analysis with 73% of total information demonstrated efficacy.

***Safety.*** The expected rate of total AEs following circumcision is expected to be ~ 2%. This will continuously monitored during the trial and specifically assessed by the DSMB after completion of Stage 1 (~ 4-7 months), and at 20 and 31 and 40 months. The DSMB will be blinded to any tabulations by study arm.

***Acceptability.*** Acceptability of circumcision should be  50% among eligible men. If acceptance is less, the DSMB will evaluate the data from the pilot study to determine whether **to** proceed with the full-scale randomized clinical trial, to change accrual methods or to propose an alternative design.

**Stopping rules:** The following stopping rules are suggested**:**

***Efficacy.*** We propose an analysis at 40 months. If HIV incidence in one arm significantly exceeds that of the other arm, it is proposed that the trial be discontinued following DSMB review and discussion with Program. We suggest that formal statistical monitoring methods such as the Lan-DeMets group sequential approach ^118^ with an O’Brien-Fleming type spending function. These methods minimize the chance of inappropriately terminating the trial in its early stages. If interim analyses suggest equivalence of HIV incidence in the two arms, conditional power methods will be used to estimate the chance that further data accumulation will result in significant differences. If the probability is low, consideration will be given to stopping the trial.

***Behavioral changes, STDs, and disinhibition.*** It is possible that, despite intensive health education, circumcision may lead to false expectations of protection, engendering increased risk behaviors. If there is evidence of increased risk behaviors we would intensify health education efforts. However, if such improved education is unsuccessful, we suggest establishing stopping rules using the following possible measures of disinhibition in the intervention versus the control arm: a) a significant decline in the frequency or consistency of condom use, b) a significant increase in the number of extramarital partners, c) a significant increase in use of alcohol with sex, d) a significant increase in HIV incidence or selected STDs which are markers for risk behaviors (e.g., incident syphilis).

**8. HUMAN SUBJECTS**

We will first describe services provided by the Rakai Health Sciences Program and relevant actions as background to consideration of human subject issues.

**8.1. Services of relevance to Human Subject Considerations**

**8.1.a. Community Advisory Board (CAB)**

The CAB reviewed the proposed trial and their letter of approval and list of members is attached. The CAB recommended that efforts be made to promote condom use among circumcised men, that well fitting garments and transport be provided to men immediately after circumcision if required, and that compensation be offered ($15.00) for lost time following surgery. These recommendations are incorporated into the proposal. The CAB recommended that men over age 49 be provided with circumcision if they request the procedure, and subject to DSMB approval, we will make this service available after completion of the trial. The CAB did not express reservations about the delayed circumcision provided in the control arm, nor about randomization. With assistance of the CAB and our community mobilization team we established liaison with Christians and animist religious leaders, to address any concerns about circumcision.

**8.1.b. Condom Promotion:**

Condoms were provided free to all enrolled men who were advised to practice consistent use, and to obtain re-supplies from community depots maintained by the Program. Circumcised men will were advised to only resume intercourse after certified wound healing and **to practice consistent** condom use. Current condom use has increased from 9.9% in 1995 to > 30% in 2001. Use with extramarital partners was 30.8% in 1995 and is currently over 60%. Thus, the Rakai Health Sciences Program has markedly increased condom use by the community-based education and distribution program. However, despite repeated intensive Rakai Health Sciences Program condom promotion via community meetings, counseling, and community distribution of supplies, consistent condom use remains low (~ 8 % in 2001), hence HIV incidence is stable at around 1.4/100py, and there is a need for innovative prevention interventions.

**8.1.c. Voluntary HIV Counseling and Testing (VCT):**

The Project strongly promotes and provides free VCT on request to individuals or couples, and VCT is encouraged at community meetings and community counseling sessions. Ready access to VCT is provided by trained full-time counselors resident in the communities and 2 counseling supervisors. VCT is provided in the home or at the counselors office, at the client’s discretion. Pre- and post-test counseling and long-term counseling support are available. The counseling messages include information on HIV/STD transmission and prevention including fidelity, abstinence, condom use and other safe sex behaviors, partner notification and couples counseling, and general health. Post-test counseling includes interpretation of HIV results, prevention of transmission/acquisition and support. We adhere to Ugandan Government policies on VCT^83b^ which explicitly require that VCT be voluntary and preclude disclosure of results to third parties, including sexual partners, without the written permission of the index individual.

**8.1.d. General medical Care and Health Education.**

All trial participants have access to free general health care provided by Rakai Health Sciences Program at time of scheduled contact. Between surveys, care is available via Government health services. The Project provides free health education, condom promotion and free condoms to all residents (participants and non-participants**).**

**8.2. Institutional Review Board (IRB) Reviews.**

The study was reviewed and approved by the Scientific and Ethical Committee (SEC) of the Ugandan Virus Research Institute (FWA 00001254, expiry 8/30/04), Johns Hopkins University Committee for Human Research (CHR# H.32.02.05.10.B), and Columbia University Institutional Review Board (IRB# 14741).

We are collaborating with Dr. Bernard Lo, Bioethicist, who advises us regarding resolution of ethical questions throughout the trial. We get annual continuing review approvals from SEC and WIRB.

**8.3. Training in Research Ethics:**

All Rakai Health Sciences Program senior investigators in the US and Uganda, have taken and passed courses in Research Ethics/Good Clinical Practice. All other Ugandan project personnel having contact with participants, participant data or laboratory samples, have been trained in Uganda, based on materials from Johns Hopkins and Columbia.

**8.4. Proposed involvement of Human Subjects:**

The trial participants are 5,000 uncircumcised, HIV-negative adolescent and adult men aged 15-49, resident in Rakai communities in southwestern Uganda. We excluded uncircumcised boys under age 15, because the low HIV incidence in such young males would not add to the study power, and we wished to establish safety and efficacy at older ages, before assessing circumcision in boys under age 15.

Men were consented for screening and if found to be eligible, they were consented for enrollment. Consenting HIV-negative men were randomly assigned to an immediate circumcision arm (intervention arm) in which they were offered the service within approximately 1 month of enrolment, or to a delayed circumcision (control arm), in which they were offered circumcision approximately 24 months after enrolment. Circumcision is carried out by trained and supervised physicians. Participants are monitored after surgery to maximize care. Men in both arms were interviewed and asked to provide biological samples at enrolment and at 6,12 and 24 month visits

Interview information was obtained at baseline, time of surgery and at each follow up visit and included sociodemographic characteristics, risk behaviors (e.g., number and type of partners, condom use, sexual networks, alcohol use, coercive sex), health information including symptoms of STDs or penile pathology (e.g., discharge, dysuria, GUD, balanitis, post-operative infection or bleeding), and symptoms suggestive of OIs or AIDS (e.g., weight loss, chronic diarrhea, cough, KS etc). Most questions had already been tested in prior Rakai Health Sciences Program surveys.

Blood samples are collected from men for detection of HIV, STDs and hemoglobin as per protocol. Male urine samples are collected for detection of STDs with other funds. Prior to surgery, subpreputial swabs are obtained for assessment of STDs as per protocol and men reporting GUD are asked for an ulcer swab. The foreskin of circumcised men is retained for histopathology.

Subjects are identified by a permanent study ID number on all forms and samples, and a polaroid photograph is used to insure identity during follow up.

**8.5 Recruitment and Consent**

Participants were mobilised through community mobilization meetings and home visits. With the assistance of our Community Advisory Board (CAB), we informed the population of the research. Following contact with village leaders, we conducted open community meetings during which the community was informed of the nature of the trial, including randomization into one of two arms, and the possible risks and benefits of male circumcision. Following the community informational meetings, all age eligible men aged 15-49 residing in the community (identified from Rakai Health Sciences Program data files) or non-cohort participants (“walk ins”) were invited to go to the Kalisizo Clinic or central locations in the communities (“hubs”) for screening and enrollment. They were clearly informed that participation is entirely voluntary and that they would not forego any Rakai Health Sciences Program services (condoms, VCT, access to Rakai Health Sciences Program clinics) should they decline enrollment into the trial. The Screening and Enrollment Consents are given in Appendix 1, Forms I and I.a.

All prospective volunteers were offered VCT. After completing a screening consent and the screening procedures, HIV-negative men who indicated that they were willing to accept circumcision and randomization into either the intervention (immediate circumcision) or control (delayed circumcision) arms, and who agreed to VCT were asked to provide written consent for enrollment into the NIH trial. The consent process included oral group or individual presentations with a video (where possible) and an opportunity to ask questions during preliminary health education. Participants were given an opportunity to read the consent if they are literate, or to have the consents read to them if illiterate Then individual consent was then obtained. The consent included information regarding the design of the trial, requirements for participation including follow-up and data/sample collection, the risks and potential benefits of circumcision, and the freedom to refuse in part or in whole, or to withdraw from the trial, without loss of benefits and privileges. Contact names of physicians and phone numbers are provided for questions or complaints. The copy of the enrollment consent retained by the participants did not indicate HIV status or sponsor, except by a code (NIH form I.a., Gates Foundation form I..b), in order to avoid potential disinhibition among participants in the NIH trial, and to prevent breach of confidentiality and stigmatization of men enrolled in the Gates Foundation sponsored trial**.** After randomization, men completed a consent comprehension test to ensure that they understood the trial and to correct any misunderstandings

Men who were unsure about participation, but who did not refuse outright, were told that they should take time to consider their decision. They were re-contacted after 2-3 days to determine whether they decide or decline to participate.

As is standard operating procedure, consent forms were translated into Luganda, and back translated into English. A certificate of translation was obtained. Subjects were asked to sign or thumb print the consent document, and consent was independently witnessed. Enrolment of unemancipated minors aged 15-17 required both the assent of the parent/guardian and independent individual consent by the minor. A copy of the signed form was retained by the subjects, and original forms were maintained in safe, locked stores

Consent was obtained on screening, enrollment and prior to surgery.

**8.6. Ethnic Groups and Minors**

Residents of Rakai District are all Black African, and belong to several tribal groups, of which the Baganda, Banyarwanda, Bakiga and Banyankole are the largest. Men were enrolled regardless of tribal affiliation.

As indicated above, the study included respondents aged 15-17. This is in keeping with recent NIH standards that minors be included in research related to conditions which affect their health. In Rakai, HIV, STDs and genital tract conditions represent health problems at young ages. Safety and healing following circumcision needed to be examined in adolescent males, since in countries such as Kenya, there was growing evidence that males in this age group were seeking out the procedure.

Emancipated adolescents, defined as persons under the age of 18 who are heads of households, or who live independently of their parents or guardians and are not in school, were asked to provide individual informed consent. Unemancipated minors aged 15-17 were likewise asked to provide individual informed assent, but their enrolment was also contingent on written consents by the parent or guardian. (Please note: if the parent or guardian consented but the minor did not assent, that minor was not enrolled.**)**

**8.7 Potential Risks to Participants**

**8.7.a Risks of circumcision:**

The rate of complications following circumcision reported in the clinical literature is less than 2% and include transient hyperesthesia, hematoma, bleeding and infection. Damage to the glans or shaft is extremely rare. Physical activity or intercourse early after surgery could exacerbate bleeding, bruising or infection. Intercourse before wound healing could increase the risk of HIV acquisition. Allergy, including anaphylaxis can occur with local anesthesia and antiseptics. Drowsiness or unsteadiness can occur with pain medications.

**8.7.b Confidentiality and social harm.**

There is a risk of breech of confidentiality if patient records, interviews or lab results are revealed to third parties. There is a risk that misidentification could cause an individual to be given an incorrect diagnosis or inappropriate treatment, or be inadvertently denied indicated treatment. There is a risk that knowledge of positive HIV results received via voluntary counseling and testing (VCT) could lead to distress, marital disruption or partner abuse.

**8.7.d Disinhibition:**

If circumcised men believe themselves to be at reduced risk of HIV or STD infections, they may adopt higher risk behaviors. If the copy of the consent form retained by HIV-negative men enrolled in the NIH trial documented HIV status, this “proof” of negative status could be used to negotiate unsafe sexual behaviors. To avoid this dilemma, the copy of the consent provided to participants will not indicate HIV status directly, or indirectly (e.g., by specifying NIH or Gates trial enrollment).

**8.7.e Risk related to biological specimen collection.**

Blood and urine collection is minimally invasive and entails minimal risk. Foreskins are saved for separate studies: the risk of collection is summarized under risks of circumcision.

**8.8 Procedures for Protecting Against Risk.**

**8.8.a Risks of circumcision:**

The risk of complications during or following circumcision is minimized by careful training and certification of physicians, supervision by an experienced urologist and referral/exclusion of men with anatomic abnormalities of the genitalia. A physician at the Kalisizo Hospital is available to assist with surgical complications. The hospital is only minutes away from the Rakai clinic facility. The risk of infection is minimized by: i) In men with evidence of current infection, surgery is deferred, and men are treated with antibiotics until infection has resolved. ii) Surgery is performed in a dedicated surgical facility, with strict adherence to aseptic procedures, autoclaving of all instruments, and careful dressing and monitoring of wound healing. iii) Any postoperative infection is treated with antibiotics and, if indicated, men are hospitalized at project expense for wound care. The risks of bleeding or bruising are minimized by: i) careful surgical technique and attention to bleeders, ii) use of an occlusive dressing, iii) postoperative bed rest and provision of well fitting garments and transport home, iii) encouraging participants to refrain from physical work and activity for approximately 3-5 days, and to refrain from intercourse until complete wound healing is certified. Men are provided with clear information on risks of premature resumption of physical work, and compensation is paid for work time lost (the equivalent of $15, to enable them to take the day of surgery, and the subsequent 3-5 days, off work). Potential risk of HIV is minimized by advising men to refrain from intercourse until the wound is healed, and promotion of condoms when intercourse is resumed. We provide detailed information and follow up for subjects to minimize risks. Postoperative pain is controlled by analgesia and limitation of activity. Men experiencing hyperesthesia are told this is usually transient, but if it does not resolve within a week, they are referred to the urologist for free care.

**8.8.b Confidentiality and social harm**

The Rakai Health Sciences Program has, for fourteen years, maintained full confidentiality of participant records. Informed consent documents are retained in locked filing cabinets and store rooms, accessible only to senior investigators or designated staff. Case report forms retain participant’s names until completion of analysis. All questionnaires are stored in secure, locked facilities. Only designated staff have access to these records. Photos used to identify persons are kept in locked cabinets and are under the control of the study supervisor. All computerized data bases are password protected. Electronic records containing confidential or sensitive information only contain the study ID # without the participant’s name. Files of lab results are maintained in a separate safe computer file, with study ID numbers, and contain no personal identifiers.

Misidentification is avoided by the photo ID and by use of unique, alphanumeric check digit ID numbers. The check digit number avoids data entry errors. All documents and samples are labeled with pre-printed study numbers to avoid transcription errors. Lab results are double entered to avoid data keying errors.

**8.8.c HIV Voluntary Counseling and Testing (VCT): protection of confidentiality**

Individuals and couples in the Rakai cohort are offered VCT, and those who accept are flagged for expeditious assay. HIV results are provided on the basis of two concordant EIAs (i.e., concordantly negative or positive). If EIAs are discordant, results are not provided until WB confirmation is available. The following procedures are followed to minimize risk of social or psychological harm that might result from breech of confidentiality. HIV result letter used by the counselor to inform the participant, contains the study ID# and the participant’s name to insure correct identification, but the HIV result is coded so as to be un-interpretable by a third party.

Participation in VCT (both individual and couples counseling), although strongly encouraged, is entirely voluntary. Counselors provide pre-results counseling for all participants and inform them of the potential for social harm, and advise them on how to mitigate such consequences. Participants may opt to learn their results and receive post-test counseling, or they may decline to learn their results. Only men who agree to learn their HIV results are enrolled in the NIH sponsored trial. Results are provided in private to individuals or couples (if both individuals have elected to participate in couples counseling). If recipients opt for individual counseling, they are encouraged to inform their partners of the test results but, in accord with Ugandan Government policy, no results are revealed to third parties, including spouses, without an individual’s express, written permission.

**8.8.d Disinhibition:**

To avoid false expectations of protection by circumcision we carefully informed participants that the efficacy of the interventions was unknown at that time, that monogamy with an HIV-negative partner or consistent use of condoms were the only proven methods of preventing infection, and that avoidance of high risk behaviors is imperative to reduce HIV risk. (Condoms are provided free of charge by the Project and we recommend that condoms be used by all men). Because we have designed the study within Rakai communities, all members of the community - men and women - are informed via town meetings of the need to continue other safe sex practices. The message is reiterated during each community town meeting for the duration of the study.

To minimize disinhibition among men in the NIH trial, and to prevent the use of copies of the consent form as “proof” of HIV-negative status, the consent copies do not indicate HIV status. Disinhibition is also minimized by intensive health education and monitoring of risk behaviors both via surveys and by qualitative research methods.

We monitored behaviors carefully during the trial and if an excess of risk behaviors was observed, the DSMB would determine whether this can be rectified, or whether the trial should be terminated.

8.8.e Risks of biological sample collection.

Venipunctures and genital ulcer swabs are collected by highly trained and experienced Rakai Health Sciences Program survey and clinical personnel. Over 80,000 serological specimens and 80,000 urine specimens were collected during the Rakai STD Control Trial for HIV Prevention, with no significant adverse event.

To avoid potential risks of incorrect HIV diagnoses, results are only provided on the basis of concordant EIAs with WB confirmation of discordant EIAs.

**8.9 Study Benefits to Participants**

**8.9.a Health Care:**

Residents of Rakai Health Sciences Program study communities, whether or not they have agreed to participate in any Rakai study, are provided with health education via town meetings and are offered condoms.

All residents also have access to Rakai Health Sciences Program mobile clinics for free general health care and symptom-based STD treatment at the time of our annual survey rounds.

All residents have access to the Rakai Health Sciences Program fixed clinic in Kalisizo for STD treatment; study participants are offered STD treatment for free in the fixed clinic.

All HIV Positive residents, who are study participants, are offered free health services and treatment for OIs through the Rakai Health Sciences Program fortnight HIV clinic in our fixed facility in Kalisizo, and in one mobile clinic which services distant communities.

In addition to the benefits above, study participants are offered free, confidential individual and couples voluntary counseling and testing.

**8.9.b STD testing and treatment provided to participants**

In addition to the symptom-based STD treatment offered to all study community residents through our mobile clinics at the time of each study round, circumcision trial participants in both arms are routinely tested for syphilis, and offered treatment on the basis of results. In addition, participants are examined and any pathology diagnosed clinically is treated. We use highly effective, single dose observed therapy, as we did in the STD control trial. Drugs include azithromycin, ciprofloxacin, cefixime, metronidazole and IM benzathine penicillin. Participants in both study arms are also provided with HSV-2 suppressive therapy, if there is evidence of active herpetic ulceration at the time of the survey or prior to circumcision.

**8.9.c General health care:**

At the time of each survey round, Rakai Health Sciences Program mobile clinics offer free general health care, including antimalarials and antibiotics as required, to all study community residents. Symptom-based care includes treatment for respiratory conditions, thrush, fever, skin conditions, pain and diarrhea.

**8.9.d Treatment for Opportunistic Infections and ARVs**

As indicated above, all HIV-positive residents of Rakai study villages are offered free care at fortnightly clinics held in our Kalisizo facility and in one mobile clinic, which services distant villages. We provide prophylaxis and treatment of OIs, and ARVs via the Presidential AIDS Initiative (PEPFAR). Care is provided when appropriate and feasible.

**8.9.e Provision of free circumcision:**

Men in the intervention arm were offered free circumcision within one month of enrolment.

Men in the control arm are provided with free circumcision after 24 months follow up as a service, contingent on DSMB assessment of safety. They were told that information on efficacy was not available then, and that they could defer circumcision to the end of the study when the last men enrolled into the two study arms complete 2 years of follow up. (Please note: The DSMB stopped the trial after interim analysis showed more that 50% efficacy.). Therefore, HIV-negative uncircumcised men in the control arm of the NIH and Gates sponsored trials will be offered surgery. )

**8.9.f Condoms**

Condoms are provided free of charge to study participants via the survey teams, health education team, condom sale agents.

HIV counselors and our community mobilizers. The former reside permanently in the study communities.

**8.10. Compensation for Participants**

We proposed to provide $30.00 (approximately 52,500/= Uganda shilling) as compensation for time lost due to surgery and the time required for screening/ enrollment and follow-up. All men received $3.00 compensation at time of enrollment, and for each scheduled visit at 3-6 weeks, 6, 12 and 24 months. Men in the immediate circumcision arm received $15.0 compensation distributed as $5.00 on day of surgery, and $5.00 at each of the 48 hour and 7 ±2 days visits. Control arm men will receive $15.00 compensation for time lost due to surgery. Based on discussions with our Community Advisory Board, the proposed amount was deemed to fairly recompense participants, without being a coercive inducement. Experience during stage one suggested that this distribution of compensation can maintain high compliance rates and avoid disinhibition that might occur with larger lump sum payments.

Men will be invited to attend focal points or “hubs” located in the communities and will be compensated for transport cost.

**8.11 Benefit-Risk Ratio**

The risks of the proposed Circumcision Trial are reasonable in relation to the benefits. Adult circumcision entails relatively minor surgery under local anaesthesia, with low rates of complications. The training and supervision of physicians performing the procedure and the conditions under which it is performed should minimize these risks. Stage 1 results showed that circumcision offered in the Rakai study clinic is safe . The individual benefits of the intervention are potential protection from HIV and STDs, and improved penile hygiene. There are substantial societal benefits. It is now known that circumcision is efficacious. This has provided critical information for policy and development of programs for HIV prevention. Because circumcision is a one time procedure, such benefits could be life long**.**

The risks of breech of confidentiality are minor, and the Rakai Health Sciences Program has put in place numerous safeguards to prevent such breeches and to avoid misidentification errors. The benefits also include access to HIV results and counseling through VCT, free condoms and health care.

The risk of disinhibition could not be determined prior to study initiation, but would be offset by careful education and patient instruction. We have neither observed disinhibition in our previous intervention trials nor in this trial.

**9.0 PUBLICATION OF RESEARCH FINDINGS**

Results of the research are communicated to the Ugandan Ministry of Health and to study communities.

A Publication Committee consisting of senior investigators will be formed to decide on topics for papers, and allocation of responsibility for manuscripts. First authorship will be given to the investigator who takes primary responsibility for specific analyses and writing of papers. Papers or abstracts will be submitted only after review by the Publication Committee.

**Table 1. Time Line**

| **Activities (Project months)** | **Year 1** | **Year 2** | **Year 3** | **Year 4** | **Year 5** |
| --- | --- | --- | --- | --- | --- |
| **Stage 1**  **(~ mths 1-6)** | **xxxxxx** |  |  |  |  |
| **Assessment of acceptability/safety**  **(~ mth 6-7)** | **x** |  |  |  |  |
| **Finalize mobilization message etc.** | **x** |  |  |  |  |
| **Stage 2** |  |  |  |  |  |
| **Enrollment/Randomization**  **(~ mths 9-26)** | **xxxx** | **xxxxxxxxxxxx** | **xx** |  |  |
| **Intervention Circumcisions**  **(~ mths 9-27)** | **xxxx** | **xxxxxxxxxxxx** | **xxx** |  |  |
| **6 month follow up**  **(~ mths 14-32)** |  | **xxxxxxxxxx** | **xxxxxx** |  |  |
| **12 month follow up**  **(~ mths 20-38)** |  | **xxxx** | **xxxxxxxxxxxx** | **xx** |  |
| **24 month follow up**  **(~ mths 32-50)** |  |  | **xxxx** | **xxxxxxxxxxxx** | **xx** |
| **DSMB safety monitoring**  **(~ mths 19,31)** |  | **x** | **x** |  |  |
| **DSMB Interim Analyses**  **(~ mths 40, 56)** |  |  |  | **X** | **X** |
| **Circumcise Controls**  **(~ mths 24-56)** |  |  | **xxxxxxxxx** | **xxxxxxxxxxxx** | **xxxxxxxxxxxx** |
| **Analysis and reports**  **(~ mths 51-60)** |  |  |  |  | **xxxxxxxxxxxx** |

Table 2. Summary of Forms for Stage 2 of the Trial

| **Record Forms** | **Contacts** | | | | | | | |
| --- | --- | --- | --- | --- | --- | --- | --- | --- |
|  | **1** | **2** | **3** | **4** | **5** | **6** | **7** |  |
| **Pre-Enrollment interview for cohort participants interviewed within past 6 months ( 02a/48)** | **x** |  |  |  |  |  |  |  |
| **Screening Consent (I)** |  | **x** |  |  |  |  |  |  |
| **Enrolment Interview (02b)** |  | **x** |  |  |  |  |  |  |
| **Eligibility verification form ( 22)** |  | **x** |  |  |  |  |  |  |
| **Enrolment Consent (I.a.)** |  | **x** |  |  |  |  |  |  |
| **Interview for non-cohort participants^1^ and for cohort participants interviewed > 6 months previously ( 02a)** |  | **x** |  |  |  |  |  |  |
| **Randomization form ( 18)** |  | **x** |  |  |  |  |  |  |
| **Post-randomization Information sheet (II)** |  | **x** |  |  |  |  |  |  |
| **Surgical consent (III)** |  |  | **x** |  |  |  |  |  |
| **Pre-operative Interview/ Exam (03)** |  |  | **x** |  |  |  |  |  |
| **Surgical record (04)** |  |  | **x** |  |  |  |  |  |
| **Postoperative patient information sheet ( 46)** |  |  | **x** |  |  |  |  |  |
| **Post-operative visit forms** |  |  |  | **x...** |  |  |  |  |
| **Follow up Visits 6, 12, 24 months (12)** |  |  |  |  | **x** | **x** | **x** |  |
| **Adverse Events Reporting Form (11)** |  |  | **x** | **x** | **x** | **x** | **x** |  |
| **Protocol deviation form (13)** |  | **x** | **x** | **x** | **x** | **x** | **x** | **x** |
| **Withdrawal form (14)** |  | **x** | **x** | **x** | **x** | **x** | **x** | **x** |
| **Missed visit form (15)** |  |  | **x** | **x** | **x** | **x** | **x** | **x** |
| **Control subjects consent for surgery (IV)** |  |  |  |  |  |  |  | **x** |

**^1^Men who were not previously enrolled in the cohort or cohort participants last interviewed > 6 months before enrollment will complete the interview after enrollment into the trial**

**Table 4. Number of AEs, exact confidence intervals and 1-sided p values for all AEs and for moderate/serious AEs.**

| **Number of events /**  **Estimated rate (%)** | **Exact 95% Confidence intervals** | **Exact 1-sided p-value for testing vs. 2% rate** | **Exact 1-sided p-value for testing vs. 0.5% rate** |
| --- | --- | --- | --- |
| **0** | **0.00-3.52** | **.1326** | **.6058** |
| **1** | **0.05-4.92** | **.4033** | **.3942** |
| **2** | **0.36-6.60** | **.5967** | **.0898** |
| **3** | **0.82-7.99** | **.3233** | **.0141** |
| **4** | **1.38-9.43** | **.1410** | **.0017** |
| **5** | **1.99-11.03** | **.0508** | **.0002** |
| **6** | **2.65-12.16** | **.0155** | **<.0001** |
| **7** | **3.33-13.49** | **.0041** | **<.0001** |
| **8** | **3.52-14.84** | **.0009** | **<.0001** |

**Table 5. Male HIV Incidence by Age**

| **Age groups** | **15-19** | **20-29** | **30-39** | **40-49** | **All** |
| --- | --- | --- | --- | --- | --- |
| **HIV Incidence/100 py in uncircumcised men (observed)** | **1.1** | **2.1** | **1.9** | **1.8** | **1.8** |
| **Estimated number of men enrolled per arm** | **435** | **988** | **582** | **495** | **2,500** |
