# Supplementary material for: Effects of Genital Ulcer Disease and Herpes Simplex Virus Type 2 on the Efficacy of Male Circumcision for HIV Prevention: Analyses from the Rakai Trials
Source: PLoS Med. 2009 Nov 24;6(11):e1000187. doi: 10.1371/journal.pmed.1000187 (PMC2771764; doi:10.1371/journal.pmed.1000187)
Supplement: Text S2 — Gates Trial protocol. Protocol for trial of MC in HIV-positive men and their partners, and HIV-negative men who declined post-test VCT. (0.73 MB DOC) [file pmed.1000187.s002.doc]

**RANDOMIZED TRIAL OF MALE CIRCUMCISION: STD, HIV AND BEHAVIORAL EFFECTS IN MEN, WOMEN AND THE COMMUNITY.**

**PROTOCOL**

Principal Investigators: Maria J. Wawer, Columbia University

David Serwadda, Makerere University

Co-Investigators: Ronald H. Gray, Johns Hopkins University

Stephen Gange, Johns Hopkins University

Nelson Sewankambo, Makerere university

Fred Wabwire-Mangen, Makerere University

Tom Lutalo, Uganda Virus Research institute

Stephen Watya, Makerere University

Noah Kiwanuka, Rakai Health Sciences program

Godfrey Kigozi, Rakai Health Sciences program

Fred Nalugoda, Rakai Health Sciences program

Consultants Bernard Lo, UCSF

Roberta Heath, Johns Hopkins University

Study Site: Rakai District, Uganda

Period of Study 7/1/02 to 6/20/07

Contact Address

Maria J. Wawer MD MSH

Professor

Heilbrun Center for Population and Family Health

Columbia University

Floor B-2

60 Haven Avenue

New York

NY 10032

212 304 5278

Email: mwawer@jhsph.edu

**TABLE OF CONTENTS**

[1 INTRODUCTION 3](#__RefHeading___Toc86134306)

[1.1 Background 3](#__RefHeading___Toc86134307)

[1.2 Rationale 3](#__RefHeading___Toc86134308)

[1.3 Study Designs 3](#__RefHeading___Toc86134309)

[2 STAGE 2 STUDY OBJECTIVES 3](#__RefHeading___Toc86134310)

[2.1 Primary objectives: 3](#__RefHeading___Toc86134311)

[2.2 Secondary objectives: 3](#__RefHeading___Toc86134312)

[2.3 Time Line 3](#__RefHeading___Toc86134313)

[3 SELECTION AND ENROLMENT OF SUBJECTS 3](#__RefHeading___Toc86134314)

[3.1 Study Populations 3](#__RefHeading___Toc86134315)

[3.1.1 Gates Supported Circumcision Trial Populations:. 3](#__RefHeading___Toc86134316)

[3.1.2 Rakai Community Cohort Surveillance Study (RCCS) Populations:. 3](#__RefHeading___Toc86134317)

[4 GATES SPONSORED CIRCUMCISION TRIAL METHODS 3](#__RefHeading___Toc86134318)

[4.1 Inclusion Criteria for the Gates sponsored Circumcision Trial: 3](#__RefHeading___Toc86134319)

[4.2 Exclusion Criteria for the Gates Sponsored Circumcision Trial 3](#__RefHeading___Toc86134320)

[4.3 Enrolment and Randomization Procedures 3](#__RefHeading___Toc86134321)

[4.3.1 Community Mobilization 3](#__RefHeading___Toc86134322)

[4.3.2 Screening, Enrollment and Informed Consent 3](#__RefHeading___Toc86134323)

[4.3.3 Procedures at Enrollment 3](#__RefHeading___Toc86134324)

[4.3.4 Randomization 3](#__RefHeading___Toc86134325)

[5 STUDY TREATMENTS 3](#__RefHeading___Toc86134326)

[5.1 Surgery 3](#__RefHeading___Toc86134327)

[5.1.1 Pre-operative history and examination 3](#__RefHeading___Toc86134328)

[5.1.2 Surgical Procedure: 3](#__RefHeading___Toc86134329)

[5.1.3 Postoperative care 3](#__RefHeading___Toc86134330)

[5.1.4 Asepsis 3](#__RefHeading___Toc86134331)

[5.1.5 Emergency procedures: 3](#__RefHeading___Toc86134332)

[5.2 Concomitant Medications 3](#__RefHeading___Toc86134333)

[5.2.1 Analgesia 3](#__RefHeading___Toc86134334)

[5.2.2 Treatment of Infections 3](#__RefHeading___Toc86134335)

[5.2.3 Treatment of Anemia 3](#__RefHeading___Toc86134336)

[6 CLINICAL AND LABORATORY EVALUATIONS 3](#__RefHeading___Toc86134337)

[6.1 Screening Evaluations 3](#__RefHeading___Toc86134338)

[6.1.1 Screening Interview and Exam 3](#__RefHeading___Toc86134339)

[6.1.2 Eligibility 3](#__RefHeading___Toc86134340)

[6.1.3 Enrollment and Randomization 3](#__RefHeading___Toc86134341)

[6.1.4 Sociodemographic and Behavioral Interview 3](#__RefHeading___Toc86134342)

[6.2 Evaluation Related to Surgery 3](#__RefHeading___Toc86134343)

[6.2.1 Preoperative Assessment 3](#__RefHeading___Toc86134344)

[6.2.2 Surgery 3](#__RefHeading___Toc86134345)

[6.3 Post-Surgical Evaluations for Intervention Arm, and Post-Enrollment Evaluations for Control Arm Participants in stage 2 3](#__RefHeading___Toc86134346)

[6.3.1 Post-operative Evaluations 3](#__RefHeading___Toc86134347)

[6.3.2 Scheduled follow up - Both arms 3](#__RefHeading___Toc86134348)

[6.3.3 Evaluations at the time of premature discontinuation 3](#__RefHeading___Toc86134349)

[6.4 Laboratory Methods for Evaluations 3](#__RefHeading___Toc86134350)

[6.5 Behavioral Research Methods for Evaluations. 3](#__RefHeading___Toc86134351)

[7 DATA COLLECTION, SITE MONITORING AND ADVERSE EXPERIENCE REPORTING 3](#__RefHeading___Toc86134352)

[7.1 Records to Be Kept. 3](#__RefHeading___Toc86134353)

[7.2 Data Management 3](#__RefHeading___Toc86134354)

[7.3 Clinical Site Monitoring and Record Availability 3](#__RefHeading___Toc86134355)

[7.4 Serious Adverse Event Reporting 3](#__RefHeading___Toc86134356)

[7.4.1 General Definitions 3](#__RefHeading___Toc86134357)

[7.4.2 Severity of AEs 3](#__RefHeading___Toc86134358)

[7.4.3 Relationship of AEs to the intervention 3](#__RefHeading___Toc86134359)

[7.4.4 Study Definition of Adverse Events 3](#__RefHeading___Toc86134360)

[7.4.5 Adverse Event Type 3](#__RefHeading___Toc86134361)

[7.5 A. During Surgery 3](#__RefHeading___Toc86134362)

[7.5.1 B. First Month Post-Surgery 3](#__RefHeading___Toc86134363)

[7.5.2 Management of AEs 3](#__RefHeading___Toc86134364)

[7.5.3 Reporting of AEs 3](#__RefHeading___Toc86134365)

[7.5.4 Tabulations of AEs 3](#__RefHeading___Toc86134366)

[7.5.5 Source documentation 3](#__RefHeading___Toc86134367)

[8 STATISTICAL CONSIDERATIONS 3](#__RefHeading___Toc86134368)

[8.1 Preamble 3](#__RefHeading___Toc86134369)

[8.1.1 Initial Assessment of Safety. 3](#__RefHeading___Toc86134370)

[8.1.2 Ongoing Monitoring of Circumcision Safety During the Trials 3](#__RefHeading___Toc86134371)

[8.2 Primary Endpoint: Male HIV Incidence. 3](#__RefHeading___Toc86134372)

[8.3 Male STDs and STD Symptoms: 3](#__RefHeading___Toc86134373)

[8.4 Female HIV Acquisition*.* 3](#__RefHeading___Toc86134374)

[8.5 Behavioral disinhibition: 3](#__RefHeading___Toc86134375)

[8.6 Accrual and Feasibility: 3](#__RefHeading___Toc86134376)

[8.7 Randomization and Stratification 3](#__RefHeading___Toc86134377)

[8.8 Analysis Plan 3](#__RefHeading___Toc86134378)

[8.8.1 Primary Endpoint, HIV incidence in Males. 3](#__RefHeading___Toc86134379)

[8.8.2 Female HIV Acquisition 3](#__RefHeading___Toc86134380)

[8.8.3 Secondary Endpoints: 3](#__RefHeading___Toc86134381)

[8.9 Interim Data and Safety Monitoring 3](#__RefHeading___Toc86134382)

[9 RAKAI COMMUNITY COHORT STUDY (RCCS) 3](#__RefHeading___Toc86134383)

[9.1 Populations 3](#__RefHeading___Toc86134384)

[9.2 Inclusion Criteria for the RCCS Populations 3](#__RefHeading___Toc86134385)

[9.3 Brief description of the cohort surveillance 3](#__RefHeading___Toc86134386)

[9.4 Enrollment of women who are not RCCS participants 3](#__RefHeading___Toc86134387)

[9.5 Utilization of information from the RCCS 3](#__RefHeading___Toc86134388)

[10 HUMAN SUBJECTS 3](#__RefHeading___Toc86134389)

[10.1 Services of relevance to Human Subject Considerations 3](#__RefHeading___Toc86134390)

[10.2 Institutional Review Board (IRB) Reviews. 3](#__RefHeading___Toc86134391)

[10.3 Training in Research Ethics: 3](#__RefHeading___Toc86134392)

[10.4 Proposed involvement of Human Subjects: 3](#__RefHeading___Toc86134393)

[10.5 Recruitment and Consent 3](#__RefHeading___Toc86134394)

[10.6 Ethnic Groups and Minors 3](#__RefHeading___Toc86134395)

[10.7 Ethical Justification for Inclusion of HIV-Positive Men and Men who Decline VCT 3](#__RefHeading___Toc86134396)

[10.7.1 Direct Benefit of Circumcision for HIV+positive Men: 3](#__RefHeading___Toc86134397)

[10.7.2 Ethical Dilemmas that would arise if HIV positive men were excluded: 3](#__RefHeading___Toc86134398)

[10.7.3 Safety and Programmatic Considerations 3](#__RefHeading___Toc86134399)

[10.8 Ethical Considerations for Inclusion of Women. 3](#__RefHeading___Toc86134400)

[10.8.1 Potential Benefits to Women: 3](#__RefHeading___Toc86134401)

[10.8.2 Ethical Dilemmas that Arise if Women were Excluded: 3](#__RefHeading___Toc86134402)

[10.9 Potential Risks to Participants 3](#__RefHeading___Toc86134403)

[10.10 Procedures for Protecting Against Risk. 3](#__RefHeading___Toc86134404)

[10.11 Study Benefits to Participants 3](#__RefHeading___Toc86134405)

[10.12 Compensation for Participants 3](#__RefHeading___Toc86134406)

[10.13 Benefit-Risk Ratio 3](#__RefHeading___Toc86134407)

[11 PUBLICATION OF RESEARCH FINDINGS 3](#__RefHeading___Toc86134408)

12 EXPERIENCE OF INVESTIGATORS

13 LITERATURE CITED

14 APPENDICES

**CASE REPORT FORMS- 2^ND^ STAGE**

00.Registration form.

01.Screening Interview and Exam.

02.a.Pre-baseline Interview

02.b.Enrolment Interview

03.Pre- operative Interview and Exam

04.Surgical and postoperative record

05.First day POP follow up Interview and Exam

07.First week POP follow up Interview and Exam

08.Second week POP follow up Interview and Exam

09.a. Third week POP follow up Interview and Exam

09.b. Fourth week POP follow up Interview and Exam-Immediate

09.c. Fourth week follow up Interview and Exam -Delayed

09.d. Fifth week POP follow up Interview and Exam

09.e. Sixth week POP follow up Interview and Exam.

10.Syphilis result form

11a. Adverse Event report form

11b. Adverse Event clinical monitoring form

12a. Sixth month follow up questionnaire

12b.12 Monthly Interview and Exam

12c. 24 Monthly Interview and Exam

13.Protocol deviation form

14.Withdrawal form

15. Missed visit/ Refusal.

16.Unscheduled visit form

17.Visit Track card

18.Randomization sheet

19.a. Screening verification flow sheet.

19.b. Enrolment verification flow sheet

19.c. Surgical verification flow sheet.

19.d. FUP verification flow sheet

20.Emergency Equipment inventory checklist.

21.b.Gates rejection form

22.Eligibility Verification form

23.Sterilizer QA Record

24.Circumcision instrument set

25.Communication Flag

27.Daily productivity log sheet

28.Linkage form.

29.VCT Result communication form

30.VCT Counselor’s checklist

31. Pre-test counseling communication form

32.VCT Result letter.

33.Mobilization/Health education guide.

34.Sterilizer Record log

35.MOH-STD FORM 4 (Partner notification)

36.Appointment card

37.Error detection sheet

38.Re-interview/Tape recording sheet

39.Certification assessment form

40.Verbal autopsy.

41.Verify biological monitoring card

41.a. Verify biological monitoring control card

42.Invitation to enroll in RCCS

43.Comprehension test interview

44.Spouse referral form

45.RCCS-Circumcision referral form.

46. Postoperative Patient information sheet

48.RCCS FUP Interview

Lab Form # 001 Specimen collection form.

Lab Form # 002 HemoCue Collection and Result Form

Lab Form # 003 Rapid HIV testing worksheet.

Lab Form # 004(a) Blood receipt form

Lab Form # 004(b) Urine receipt form.

Lab Form # 004(c) PCR swabs log form

Lab Form # 004(d) HPV shipper log form.

Lab Form # 005(a) RPR card test.

Lab Form # 005(b) TPPA confirmatory test.

Lab Form # 006 Wound infection culture and Gram stain results form

Lab Form # 007 Foreskin log form

Lab Form # 015 Participant result form.

**CONSENT FORMS.**

1. Screening consent

I.b. Enrollment consent Gates.

II.a. Post randomization Information sheet- Immediate

II.b. Post randomization Information sheet- Delayed

III Surgical consent Form

IV Control subjects consent for surgery

V RCCS Baseline Consent

VI RCCS Follow-Up Consent

# INTRODUCTION

## Background

The DSMB closed enrollment of the trial on December 19, 2006 and requested an interim analysis to assess possible harm associated with non-significant increased rates of male-to-female HIV transmission. Circumcision of HIV+ men was paused until this review. The interim analysis suggested increased rate of male-to-female transmission among couples who resumed sex five or more days prior to certified wound healing. At a DSMB review of the interim analysis on March 1, 2007 it was recommended that a protocol be developed for more intensive follow up of circumcised HIV+ men to assess wound healing, and that this protocol be reviewed and approved by the DSMB and IRBs before resuming circumcision of HIV+ participants. The amendments are given in sections 6.1 and 7.3.1.

**Circumcision in HIV-Negative Men and Risks of HIV/STD Acquisition**

***Circumcision and HIV risks in HIV-negative men:***

Three randomized trials have shown that circumcision reduces male HIV incidence by 50-60%. Many, but not all cross-sectional epidemiologic studies, particularly from Africa, show lower HIV prevalence rates among circumcised compared with uncircumcised heterosexual men.^1-8, 23-27^ In addition, ecologic analyses suggest that the distribution of prevalent HIV is inversely correlated with the prevalence of male circumcision.^28-29b^ A meta-analysis which included 18 cross-sectional or case-control studies of prevalent HIV and 3 cohort studies (two from the Rakai Health Sciences Program), estimated an adjusted relative risk of HIV infection in circumcised relative to uncircumcised men of 0.42 (CI 0.34-0.52).^3^ The reduction in risk was even more pronounced in men with high HIV exposure (RR=0.29, CI 0.20-0.41).^3^ In Rakai, HIV risks were lower in circumcised men both for incident infection (RR=0.53, CI0.3-0.9)^1^ and prevalent HIV (OR=0.41, CI 0.3-0.5).^2^ The protective effects were most marked in highly exposed men in HIV-discordant couples: within this subgroup, there were no seroconversion in circumcised HIV-negative men, compared with an incidence of 16.7 per 100py in uncircumcised men (p = 0.0004).^1,30^ A study of high risk trucking company employees in Kenya also found reduced HIV acquisition in circumcised men (RR= 0.25, CI 0.1-0.53).^5,31^ In Kenyan men with GUD, HIV incidence was lower among those who were circumcised (RR=0.41, CI 0.23-0.93).^5a^ Circumcision is also associated with a lower risk of HIV in homosexual men.^32^

In Rakai, the protective effects of circumcision are most pronounced among men in whom the procedure was performed before puberty. Compared with uncircumcised men, pre-pubertal circumcision ( 12 years) was associated with significantly reduced risks of prevalent HIV (OR = 0.39, CI 0.29-0.53), as was circumcision performed between the ages 13-20 years (OR = 0.46, CI 0.28-0.77). However, risks of prevalent infection were not significantly reduced with circumcision after age 20 (OR= 0.78 (CI 0.42-1.43).^2^ Circumcision before puberty was also associated with a significantly lower rate ratio of incident HIV (RR = 0.49, CI 0.3-0.8), compared with uncircumcised men, whereas circumcisions after age 12 years was not significantly protective (RR = 0.70, CI 0.3-1.6).^1^ The apparent attenuation of protective effects with older age of circumcision probably reflects confounding by medical indication. In Rakai, 75% of post-pubertal circumcisions were performed for health indications (e.g., phimosis), and it is likely that such pathology is, in part, a consequence of prior STD infections, which are markers for high risk behaviors.^1^ However, it is also possible that scar formation following adult circumcision may result in more friable tissue and less keratinization than with pre-pubertal circumcision, and a friable scar might increase vulnerability to HIV infection among men circumcised in adulthood, particularly if intercourse is resumed before complete wound healing. It is also possible that preexisting infections, which are indications for adult circumcision, may retard healing. We do not know of other data on HIV risk in relation to age of circumcision or indications for the procedure, nor are we aware of information on scar formation or whether healing is delayed by preexisting infections.^9^ Thus, although adolescent or adult circumcision is likely to be protective against HIV, observational data cannot quantify the magnitude of this effect, due to probable confounding by indication and factors affecting postoperative tissue repair. Data are urgently needed to determine and optimize safety of post-pubertal circumcision, and to assess whether preceding pathology may affect HIV acquisition or wound healing.

***Circumcision and risks of STDs and STD symptoms in HIV-negative men***

Circumcision is associated with reduced male risks of viral and bacterial STDs,^24,25,33-38^ genital ulceration,^2,924,37,39^ urinary tract infections^41-43^ and balanitis.^45,46^ In Rakai, GUD is significantly less frequent in circumcised compared with uncircumcised men (RR=0.70, CI 0.54-0.93),^2^ and symptoms of penile inflammation or infection consistent with balanitis are also lower among circumcised men (RR= 0.51, CI0.33-0.77 , unpublished). Circumcision is also associated with reduced risk of penile cancer due to HPV.^44,47^ Thus, the benefits of circumcision may extend to a wide range of STDs and other genital tract infections. This is important because the circumcision effect on HIV may be mediated by protection from STDs.

## Rationale

Observational data and three randomized trials show that male circumcision reduces the rate of HIV infection in men by approximately 50-60%. Thus circumcision is an important public health measure because, despite initial costs, it could provide long-term risk reduction in males and secondary protection against infection in their female partners, without incurring recurrent costs. However, the possible efficacy of circumcision for prevention of HIV in women is unclear.

## Study Designs

We have conducted two complementary individually randomized, unblinded, two armed trials to assess the efficacy of circumcision for prevention of HIV and STD acquisition in men and women in rural communities of Rakai District, Ugandan. The two trials and study populations are summarized in Table 1 and are as follows:

## Summary of the Gates and NIH supported trials

| Populations | **NIH Trial** | | **Gates Trial** | |
| --- | --- | --- | --- | --- |
| **Trial** | **Eligible population** | **N** | **Eligible population** | **N** |
| Males | HIV-, accept VCT, | 5000 | HIV+ | ~ 800 |
|  |  |  | HIV-neg decline VCT | ~ 1000 |
|  |  |  | HIV-neg men who accept VCT , after NIH-funded study completed enrolment | ~500 |
| **Surveillance Cohort** |  |  |  |  |
| Females | na |  | All women in the surveillance cohort | ~ 6000 |
| Males | na |  | Men enrolled in surveillance cohort who decline trial enrollment | ~ 3000 |

***a. NIH Supported trial of male circumcision for prevention of HIV and STD acquisition in HIV- men.***

NIH has supported a trial of 5,000 HIV-negative men who agree to learn their HIV results. The trial was closed early due to efficacy on December 12, 2006, and a paper reporting the findings has been published in Lancet. Circumcision is now being offered to all HIV-negative controls.

***b. Gates Foundation supported trial male circumcision: STD, HIV and behavioral effects in men, women and the community.***

The complementary but separate Gates Foundation supported trial enrolled HIV+ men and men who decline to learn their HIV results. This trial has been conducted concurrently in the same communities as the NIH funded trial, using identical procedures.

In addition, the Gates sponsored study is assessing the safety of circumcision in HIV+ men, the efficacy of circumcision to prevent HIV and STDs in female partners, and the effects of circumcision on risk behaviors (i.e., disinhibition) in enrolled men, their partners and the community.

This protocol summarizes the Gates Foundation trial.

#

# STAGE 2 STUDY OBJECTIVES

## Primary objectives:

1. To assess the efficacy of male circumcision for prevention of HIV negative men and women.

2. To assess the safety of circumcision in HIV positive men and their partners.

## Secondary objectives:

1. To assess the efficacy of male circumcision for prevention of STD infection in men and women.

2. To assess behavioral disinhibition (i.e., adoption of higher risk behaviors) in men, women and the general community following the introduction of male circumcision.

3. To assess the acceptability of circumcision to men and women.

## Time Line

The time line is given in Table 1

# SELECTION AND ENROLMENT OF SUBJECTS

## Study Populations

Two populations will be studied (see Table 1):

### Gates Supported Circumcision Trial Populations: Men enrolled in the randomized trial of male circumcision consisted of ~ 997 HIV+ positive uncircumcised men and ~ 1000 HIV- negative uncircumcised men who decline to learn their HIV results. On the recommendation of the DSMB, enrollment into the Gates trial was closed on December 19, 2006, but follow up is ongoing.

### These trial participants were identified from

1. Men who were enrolled in the ongoing Rakai Community Cohort Study (RCCS). These men are termed “cohort participants”.

2. Men who were not RCCS participants but resided in accessible communities accessible These men are termed “walk in” participants.

### Rakai Community Cohort Surveillance Study (RCCS) Populations: Women n ~ 6000) and men not enrolled in the circumcision trial (~ 3000), who are participants in the RCCS.

To simplify the description of the study design we will first summarize the Gates sponsored circumcision trial and then summarize the RCSS populations.

# GATES SPONSORED CIRCUMCISION TRIAL METHODS

The trial is monitored by an Independent Data Safety and Monitoring Board (DSMB).

## Inclusion Criteria for the Gates sponsored Circumcision Trial:

***Male eligibility criteria for the trial are:***

- HIV-positive uncircumcised men

- HIV-indeterminate uncircumcised men

- HIV-negative uncircumcised men who declined to receive their HIV *results (Please note, these men are ineligible for the complementary NIH trial because US Government regulations require acceptance of VCT prior to HIV testing). They are included in the Gates trial because Ugandan testing policy precludes mandatory disclosure of results and allows persons who decline VCT to be enrolled into studies).*

- aged 15-49 at screening

- who accepted circumcision and agreed to randomization of timing of circumcision,

- were capable of and provided full informed consent,

- had no anatomical abnormality of the penis (e.g., hypospadias, severe phimosis) which could preclude safe circumcision,

- had no general medical condition that is a contraindication to surgery or use of local anesthesia

- hemoglobin  8 grams/dL

- intended to stay in Rakai District for at least one year, and/or available for follow up.

In addition, once enrolment of HIV-negative men who accepted VCT was completed into the NIH-funded trial (target enrolment of 5,000 men), such men were enrolled into the Gates-funded trial, until such time as the Gates study achieved its goal of ~800 HIV+ men. The HIV uninfected men were included to avoid unmasking and stigmatization of the HIV+ men, and to increase study power to assess HIV and STD endpoints in HIV-negative men, particularly in subgroups. The enrollment of HIV-negative men had equipoise because the effects of circumcision on HIV acquisition were unknown at that time.

## Exclusion Criteria for the Gates Sponsored Circumcision Trial

Men were excluded from the trial if they were:

- HIV-negative and accepted HIV result (these men were enrolled in the complementary NIH trial until such a time that the NIH study target of 5,000 HIV-neg men who accept VCT was achieved. When enrollment into NIH was completed, HIV-neg men who accepted VCT continued to be enrolled in the Gates study until such time as the complementary target of ~800 HIV+ men was also achieved. The HIV-neg men were enrolled in order to mask the HIV+ status of the primary Gates target population, and to increase the power of the Rakai studies to assess HIV and STD end points in HIV-neg men.)

- aged <15 or > 49 at screening.

- were circumcised or partially circumcised

- refused to accept randomization

- refused consent

- had anatomical abnormalities which were contraindications for circumcision

- had medical conditions which were contraindications for surgery or use of local

anesthesia.

- hemoglobin < 8 grams/dL which is not rectified by treatment

- did not intend to remain within Rakai for at least one year or are otherwise inaccessible for follow up

## Enrolment and Randomization Procedures

Study enrollment and follow up are summarized in the flow charts (Figure 1 and 2).

### Community Mobilization

All target communities were informed of the study and men were invited to participate. The community mobilization messages emphasized:

- That the Rakai Health sciences program is conducting a study to assess the acceptability and safety of circumcision, in collaboration with the Gates Foundation and NIH.

- That there is preliminary evidence from Rakai and other studies that circumcision may reduce the risk of male HIV and STD acquisition, but this has not been proven. Therefore, a randomized trial is needed to assess whether circumcision is truly protective against HIV and STDs.

- That circumcision will be offered free of charge in fully equipped surgical theaters, and will be conducted by trained physicians under local anesthesia, in conditions of careful asepsis. Pain management and careful follow up will be provided.

- That the trials will enroll both HIV-positive and HIV-negative uncircumcised men who may choose to accept or to decline HIV results

That enrollment into the trials will not disclose a person’s HIV status, which will remain completely confidential.

- That women will be included in the study to assess the possible risks or benefits of male circumcision to women

- That general RCCS community will be monitored to assess risk behaviors, irrespective of trial enrollment.

### Screening, Enrollment and Informed Consent

- Screening and enrollment was conducted by Circumcision Enrollment Teams. . Medical Officers supervise enrollment in the field and clinics. Enrollment teams generally consisted of a team supervisor, 4-5 research assistants, a data editor and a laboratory technician.

***Screening***

- Cohort participants were identified during ongoing cohort surveys. The non-cohort participants were enrolled at the Kalisizo Field Station or other “hubs”.

- Informed consent for screening was obtained

- A brief screening history and genital exam was conducted (Form 01)

- All men wrere offered VCT. Pre-result (i.e. pre-test) counseling was done in groups or individually and could also include a video. Receipt of results and post-test counseling was done individually and in private.

- Blood was collected for HIV testing and for hemoglobin measurement using a hemoglobinometer (e.g., HemoCue portable photometer). Men with Hgb < 8.0g/dL were treated for anemia, but not enrolled in the trial unless and until their Hgb  8.0g/dL

- Eligibility for the Gates or NIH trials, or ineligibility for either trial was determined Rejection form 21

***Enrollment and Randomization***

- Men eligible for the Gates trial completed an enrollment consent (Consent Form I.b).

- Enrolled men were randomized

- After randomization men were informed of their study allocation arm and provided with a post-randomization information sheet describing immediate and delayed circumcision procedures (Form 18).

- Men who were initially found ineligible due to treatable conditions, anaemia, or men report more than 30 days since last screening were re-assessed for eligibility using the eligibility re-assessment form.

- Men were provided with a circumcision study randomization ID# , as well as a Rakai alphanumeric study ID#. These were preprinted on adhesive labels which were affixed to questionnaires and samples. In addition, a photo ID was taken to allow tracking and correct identification of subjects.

- Those men randomized to immediate circumcision were scheduled for surgery and given Tetanus toxoid immunization. Arrangements were made for transport if needed.

- Men randomized to the control arm were informed that they will be re-contacted for follow up and will be offered free circumcision, contingent on DSMB approval, after 24 months

### Procedures at Enrollment

- Cohort participants who had completed the baseline survey less than six months prior to enrollment, did not require a baseline re-interview.

- Cohort participants recruited from ongoing RCCS cohort study who had completed a follow up interview on sociodemographics, risk behaviors and health more than six months before enrollment had a baseline survey administered (Form 02a).

- Non-cohort participants were asked to provide a baseline interview and samples using the current cohort questionnaires (Form 02a).

- All men received soap, and encouraged to practice penile hygiene

- Eligibility for enrollment into the Gates trial was recorded on the Eligibility verification Form 22

### Randomization

- Johns Hopkins generated a list of randomized numbers in blocks of 20 to insure comparability of men randomized to intervention and control arms, within cohort communities or groups of “walk in” participants”.

- A random assignment sheet was placed in an opaque sealed envelope generated by Rakai health sciences Program Data management using a computerized list provided by JHU, and was retained securely by the Circumcision Data Management Team.

- After enrollment and consent, men were asked to select an envelope from the block of 20 envelopes being used at that time. The envelope contained the assignment sheet showing the randomization number and random assignment to treatment or control arm. The Enrollment Team then affixed the label with the individual’s study ID number to the sheet. (Form 18). - The assignment sheet with the study ID# labeled was entered into a data base to record study arm allocation.

- The list of random allocation numbers linked to participants was retained in locked files, and in password protected computers.

- All randomization envelopes were logged out and in daily. A data base monitored used and unused allocation numbers, and tracked disposition of all envelopes.

- Men were randomized at the enrollment visit. If participants change their minds and withdrew, or at a subsequent preoperative visit, the surgeon determined that circumcision was contraindicated for medical reasons, the men were still considered enrolled and randomized. - Men were offered tetanus toxoid immunization.

-After randomization, men completed a consent comprehension test to ensure that they understand the trial and to correct any misunderstandings.

# STUDY TREATMENTS AND PROCEDURES AFTER CLOSURE OF ENROLLMENT

## Surgery

## 6.1a Trial

On the scheduled day of surgery transport was provided if needed. Men who previously consented but changed their minds and were contactable were interviewed to ascertain why they declined surgery. Repeat visits to contact absentees were conducted for a period of approximately one month, and if not contacted, their location and reason for absence were obtained from household members. Men who reported for surgery more than 30 days since last hemoglobin test had a repeat hemoglobin test. Men who changed their minds or who had a medical contraindication resulting in delayed surgery can could come for surgery within 6 months post enrollment. If at 6 months a man randomized to circumcision decided not to have surgery, he was classified as a cross-over and followed over two years**.** If a man had a contraindication that did not resolveby 6 months, he was **classified as not eligible and withdrawn from the study.**

For men who received delayed surgery between 1-6 months, the subsequent 6, 12 and 24 months visits were scheduled from time of surgery rather than from time of enrollment. For men with surgery delayed for less than 1 month (<1month), subsequent 6, 12, and 24 month follow up occured from time of enrollment.

Men were re-consented for surgery (Surgical Consent Form III), and if they agreed to surgery they were briefly interviewed and examined by a clinical assistant, and by a physician who certified fitness for surgery (Preoperative Interview/Exam Form 03).

**6.1b PROCEDURES AFTER CLOSURE OF ENROLLMENT**

All trial participants, their spouses and the community at large will be informed of the findings from the trial of HIV-negative men, and the interim findings from the trial of HIV+ men and their partners. HIV-negative uncircumcised men in the control arm of the NIH and Gates sponsored trials will be offered surgery. HIV+ men randomized to the intervention arm, and HIV+ controls who have completed 24 months follow up will be offered surgery. Men initially randomized to the intervention arm who failed to return for surgery within six months of enrollment and were classified as crossovers, will also be offered surgery

### Pre-operative history and examination

-All participants who have not received tetanus toxoid will be offered immunization.

- The genitalia will be inspected

The following additional samples will be collected: serum for HIV testing, urine and swabs from under the foreskin for future STD testing, such as gonorrhea/chlamydia/HSV-2 and HPV with separate funding. During surgery a filter paper blood spot will be collected for archival purposes.

- If there is clinical evidence of current infection (e.g., discharge, GUD), that might increase the risk of surgery, circumcision will be deferred and the patient will be treated and given penile hygiene instructions.

If there are anatomic abnormalities (e.g., hypospadias, severe phimosis, urinary retention), the patient will be referred to a consultant urologist, for evaluation. The urologist will visit the clinic approximately at 2-4 week intervals to review these cases. Men who were initially found ineligible due to treatable conditions, anaemia, or men report more than 30 days since last screening will be re-assessed for eligibility using the eligibility re-assessment form.

- For men who live a long distance from the Kalisizo clinic, a pre-operative overnight stay will be made available, if they choose to use this service.

### Surgical Procedure:

Sleeve circumcision will be performed as follows:

- The penis and both surfaces of the foreskin are preped.

- Local anaesthesia (a mixture of Linocaine and Marcaine) will be used to block the dorsal penile nerves at the base of the penis and to infiltrate the frenulum.

- With the foreskin in place and the skin lying undistorted on the shaft, a line is drawn following the outlines of the corona and the V of the frenulum. The foreskin is then retracted and a line marked 0.5-1 centimeter proximal to the corona, straight across the base of the frenulum. With the foreskin retracted the first incision follows the line marked on the inner surface, and bleeding is controlled by bipolar cautery. The incision is deepened to mobilize the skin edge. The prepuce is then extended over the glans and the marked proximal incision is made on the external surface

- The distal skin edge of the foreskin will be retracted using skin hooks and/or artery or Allis clips, or sutures to permit dissection to free the dartos fascia from the skin..

.

- The sleeve of prepuce is then removed and will be retained for pathophysiologic studies.

The foreskin will then be cut into two vertical sections placed in a preservative such as safe-fix^TM^ for archiving in Uganda and the US for histopathology].

- All bleeders are controlled,

- Skin edges re-approximated with 4-O or 5-O absorbable sutures. The first stitch is a simple suture at the apex of the frenulum followed by a horizontal mattress suture at the base of the frenulum, and then three vertical mattress sutures of each of the remaining three points of the quadrants, and a minimum of two simple sutures between each principle mattress suture (a total of 13 or more stitches) .

- The wound is cleaned with normal saline

- Medicated, non-adhesive gauze is applied and knotted on the four principle sutures, another free Medicated, non-adhesive gauze is wrapped around the first, and a regular gauze dressing applied, followed by an elastic bandage, held in place with strapping

- The procedure usually takes 45-60 minutes.

- All patients will received a tetanus booster immunization.

- The patients will recuperate for a minimum of 30 minutes or longer in a postoperative recovery room they are ready to go home.

- The surgical procedure and postoperative recovery will be recorded on Form 04.

- Men will be given well fitting garments for comfort, if needed.

Other methods of circumcision such as forceps or dorsal slit may be used

### Postoperative care

- Analgesia will be provided (see 4.2)

- Men will not be discharged if they experience severe pain, cannot walk independently or have evidence of bleeding through the dressing

- Men will be instructed not to share their pain medications. The postoperative visit will be used to monitor analgesia use.

- Men will rest for a minimum of 30 minutes or longer until they feel able to return home . All men will be examined by a physician or health worker prior to discharge and a Form 04 completed.

- Men will be provided with instructions on wound care and analgesia, and this will be reviewed with them before discharge (Postoperative patient Information Sheet 46).

.

- At time of discharge, men will be offered free transport. Men who decline or do not require transport (residence close to the Rakai Health sciences program clinic) will be advised to be accompanied by a family member, friend or Rakai Health sciences program staff.

- Free postoperative overnight stay facilities will be made available, if men choose to avail themselves of this service.

### Asepsis

- All theater staff will be trained in aseptic procedures, including documentation.

- Steam Indicators, Biological Indicators, autoclave tracking labels and instrument lists will be used to insure sterility, tracing of instruments and QC.

### Emergency procedures:

- A crash cart, IV and oxygen will be available for emergencies.

- The main potential emergency is excessive bleeding, which will be controlled by ligation of vessels and by pressure. If uncontrolled bleeding persists, a pressure dressing will be applied, and a catheter inserted if needed. If these measures do not control bleeding, an IV will be inserted and oxygen provided if needed, and the patient will be transported to hospital (Kalisizo Hospital < 5 minutes away; larger hospitals in Masaka (Masaka Hospital or Kitovu, a mission hospital ~30 minutes) away, if needed.

- Anaphylactic reaction to the local anaesthetic will be managed with epinephrine, oxygen, IV and transport to the Hospital, if needed.

## Concomitant Medications

### Analgesia

- Experience has shown that postoperative Tramadol is not needed for pain control due to the long action of the local anaesthesia. Also, Tramadol can delay discharge due to drowsiness Therefore postoperative and subsequent analgesia will use Acetominophen 325 to 650 mg, every 4-6 hours, as needed.

Postoperative analgesia with Tramadol hydrochloride 50-100 mg stat will be provided if Acetominophen does not control postoperative pain.

Men will be evaluated by a home visit the next day, and if moderate to severe pain is reported, Tramadol hydrochloride 50 - 100mg- will be dispensed.

### Treatment of Infections

*1. Treatment of STDs and Balanitis*.

- we will use symptom-based management of symptomatic infections at time of survey. In addition, syphilis treatment will be provided on the basis of serology.

- The regimen of treatment will, where possible, use directly observed, single oral therapies developed and evaluated in the STD Control Trial. The treatment algorithms include the following:

1) *GUD*. Therapy will cover *T. pallidum, H. ducreyi* and HSV-2: azithromycin 1 gram, ciprofloxacine 500 mg, IM benzathine benzylpenicillin 2.4 million units if there is serologic evidence of syphilis, acycolvir 400mg t.d.s for five days for active herpes (per PDR 2003), other therapy may be provided as medically indicated.

2) Urethral discharge/dysuria in men. Therapy will cover *N. gonorrohoeae*, and *C. trachomatis*: azithromycin 1 gram, ciprofloxacine 500 mg.

3) *Balanitis* treatment will consist of metrondizaole 2 grams stat for anaerobes, and topical clotrimazole for *C. albicans*.

*2. Pre- or post-operative penile infections* (other than balanitis) will be treated with azithromycin and ciprofloxacin. Indications for treatment are: a) clinical evidence of infection among men presenting for circumcision or after circumcision, and b) symptoms suggestive of current STDs (GUD, discharge, dysuria), reported by men during surveys or at post-circumcision visits, in both arms. Uganda MoH standard of care will be used for partner notification (MoH-STD Form 4)- Form 35 and provision of free treatment.

### Treatment of Anemia

Men with hemoglobin < 8 grams/dL, will receive iron/folate supplements and presumptive treatment for malaria and helminths using standard MoH regimens for treatment of anemia in adults. Enrollment and randomization will be deferred until the hemoglobin is increased to  8 grams/dL. If the patient does not respond to therapy, they will be referred for investigation of persistent anemia. Anemia resulting from hemoglobinopathies such as sickle cell disease will be referred.

# CLINICAL AND LABORATORY EVALUATIONS

## Screening Evaluations

### Screening Interview and Exam

- Consent for screening was obtained (Form I)

- An interview ascertained symptoms of penile pathology and other relevant medical conditions. (Form 01)

- All participants were asked to provide blood for HIV testing and hemoglobin determination to assess eligibility prior to enrollment. Blood was stored for future STD testing with other funds (See Lab methods).

- An exam was performed to verify circumcision status, and to diagnose infection, balanitis, phimosis and anatomical abnormalities (Form 01).

- Swabs were collected for future assays such as PCR, HPV and M-PCR for GUD (under separate funds).

### Eligibility

-Eligibility Screening consisted of willingness to participate, consent to circumcision and to randomization of timing of circumcision, confirmation of uncircumcised status, confirmation of HIV status, and confirmation of Hgb 8.0 g/dL. Thus, eligibility could only be determined after interview, exam and HIV/Hgb test which were recorded in the Eligibility Verification Form 22.

### Enrollment and Randomization

- After confirmation of eligibility men were consented for enrollment (Form I. b.), randomized and given a Post-Randomization Information Sheet (Form II) to inform them of their study arm allocation. Random allocation was recorded on Form 18.

### Sociodemographic and Behavioral Interview

- Cohort participants routinely complete a sociodemographic, behavioral and health follow up interview during annual cohort visits (Form 48.). For cohort participants who completed this interview within six months of enrollment, no re-interview was needed. Cohort participants interviewed more than 6 months prior to enrollment were re-interviewed to up date behavioral information.

- Non-cohort participants were interviewed using the RCCS baseline interview Form 02a

## Evaluation Related to Surgery

### Preoperative Assessment

- Prior to circumcision, men are asked to consent to surgery (amended Form III), assessed by a clinical health worker or a physician. The clinical health worker takes a general medical history, conducts a general systems exam and a genital exam (Form 03). The assessment includes general medical history and general systems exam and a genital exam (Form 03). The clinical health worker or physician examines the penis and ensures that the patient is fit for surgery. Any infection or other abnormality which might be a contraindication for surgery is treated per protocol or by referral, and surgery is rescheduled.

- Swabs are taken for future assays such as PCR, HPV and culture

### Surgery

- The foreskin with the frenulum facing upwards, will be placed on a board. The width and length will be measured in centimeters and recorded.

- Two separate quadrants with full thickness sections of the foreskin will be placed in safe-fix or other appropriate fixative and labeled with the ID#.

- A Surgical Record form (Form 06), will record the surgery and post-operative status prior to discharge.

## Post-Surgical Evaluations for Intervention Arm, and Post-Enrollment Evaluations for Control Arm Participants in stage 2

### Post-operative Evaluations

The schedule of postoperative evaluations for stage 2 of the trial is given in Figures 2

1^st^ post operative visit <48 hrs of surgery.

2^nd^ post operative visit at 7 ± 2 days following surgery.

3^rd^ post operative visit 3-6 weeks following surgery

Unscheduled visits can occur at any time if a participant experiences problems. Participants can contact the project via community counselors who are equipped with cell phones. If wound healing is incomplete at visit 3, men will be followed weekly until wound healing is certified.

These visits, which can take place in the participant’s home or at a convenient central location (“hub”), will record AEs, symptoms, resumption of intercourse, assessment of condom use, examination of the wound and collection of a culture swab if there is evidence of infection, evaluation of wound healing and certification if healing is complete.

Under this amendment, all HIV+ subjects and a sub sample of about 200 HIV negative men enrolled in the Gates funded trial will be followed at weekly intervals until complete wound healing is documented. At each visit, including the currently scheduled one and four week visits, the wound will be inspected using a magnifying glass, and records maintained of the development of scar tissue, scabs, breaches in skin integrity, adsorption of sutures, stitch sinuses, wound dehiscence, infection, hematoma and bleeding. The anatomic site of any problems will be noted. Follow up will continue until all AEs have been resolved. Records will also be maintained of wound care, resumption of intercourse and condom use. A SOP will be developed to ensure uniform certification of wound healing by clinical officers. Digital photographs may be taken to document lesions with the participant’s permission and will not include identifying facial features.. The participant will be asked to sign the photograph indicating their permission.

Consideration will be given to use of Imalux Optical Coherence Tomography (OCT) to monitor scar formation and depth (1-2 mm). However, the cost of this equipment is $60,000 and there would be delays in purchase, shipping and training for use. Therefore, rather than delay circumcision in HIV+ men, we propose to monitor healing visually until decisions on purchase of OCT equipment have been made.

At each weekly postoperative visit 5 mL of venous blood will be collected for determination of HIV viral load to assess whether surgery affects men’s viremia.

Scheduled follow up - Both arms

All men were seen 4-8 weeks post-Enrollment. For intervention arm, this coincided with post operative visit 3 to certify healing.

All participants are followed up at 6, 12 and 24 months to ascertain study end points, including HIV status (Form 12). HIV+ men and their female partners will be followed at 18 months to ascertain study end points at a time earlier than 2 years. For masking purposes, a subsample of HIV-negative men enrolled in the Gates sponsored trial, and their female partners, will also have an additional follow up at 18 months.

Evaluations at the time of premature discontinuation

If participants withdraw from the study, this will be recorded on a withdrawal form (Form 14).

## Laboratory Methods for Evaluations/

The samples are tested by two separate EIAs, and if both EIAs are negative or both are positive, the participant can be enrolled into the respective tirals. . If the EIAs are discordant, HIV status will be determined by western blot, prior to enrolment into the appropriate trial. Indeterminant Western blots will be tested by PCR.

**HIV Status.**

All participants who were tested using Rapid HIV test had their HIV results confirmed using two separate EIAs with Western blot confirmation of discordant EIA tests*.* New seroconverters are also confirmed by Western blot. Tests are run in batches and technicians blinded. If a participant refuses to provide blood during follow up, we request a urine sample for HIV testing using a urinary EIA and Western blot.

**STD Testing.** Serology is performed for syphilis and RPR-positive samples are confirmed by treponemal-specific tests such as TPHA, TPPA, or FTA-ABS.

Using other funding and separate protocols, stored samples will be tested in future for infections such as herpes simplex virus type-2 (HSV-2) infection using test kits such as the Focus EIA. *Neisseria gonorroheae* and *Chlamydia trachomatis* will be detected by PCR or equivalent methods on male urine samples Since GUD is a cofactor for HIV acquisition, all ulcers will be swabbed for future testing by PCR or equivalent methods for syphilis, *H. ducreyi* and HSV-2 in U.S.A.

**Hemoglobin**

Hemoglobin is determined using portable hemoglobinometers. A finger stick fresh blood sample is collected in a cuvette and read within 10 minutes. .

## Behavioral Research Methods for Evaluations.

Behavioral studies have already been conducted on circumcision acceptability and perceptions of risk and benefit with other funds. Behavioral research has been conducted concurrently with the trial to assess participant and community beliefs and attitudes towards circumcision, to evaluate the experience of men receiving surgery and the attitudes of control subjects allocated to delayed circumcision. These studies also provide qualitative information on participant and community beliefs about possible protective effects of circumcision which might affect behaviors, including disinhibition. Separate informed consents were developed for these behavioral study participants. Subjects included male trial participants and non-trial participants, and females (irrespective of whether their partners are trial participants or not). The methods include:

*Structured Interviews* are conducted with male trial participants at enrollment and in follow up surveys to ascertain risk behaviors (number and types of partners, coital frequency, condom use, alcohol use etc.), self-perceived HIV risk and use of VCT.

*Rapid Ethnographic Assessment (REA).* For exploration of norms, values and meanings regarding circumcision, we conducted in-depth interviews and focus group discussions with men and women aged 15-49 . This information will assist in the development of education messages on the benefits and risks of circumcision, and will facilitate interpretation of information obtained by the structured interviews. Application of multiple data collection methods permits “triangulation”, and validation of findings. The domains to be specifically explored include perceptions, facilitators and barriers to circumcision, the relationship of circumcision to sexuality, religion, and risk for HIV/STDs. Behavioral data will be collected throughout the study to adjust our education and community motivation programs as needed.

*In-depth interviews* are conducted with male and female key informants from different age groups (15-19, 20-29, 30-39, 40-49) to provide insights into the experiences, attitudes, perceptions and opinions of individuals. A total of approximately 40 informants (~5 male and ~5 female from each of the age groups), were identified through purposive sampling, based on informant’s enculturation and involvement in their respective communities. The sample included representation by residence, ethnicity, religion, economic activities, and education. Open-ended questions are used to encourage conversation and enable probing, to elicit information on experiences and on disinhibition. Although females are not enrolled in the trial, we believe it is crucial to enroll them in the REA, since their attitudes and support will be critical for male circumcision acceptance.

*Focus group discussions*. We conducted approximately 32 focus groups each stratified by marital status and gender in age groups 15-19, 20-29, 30-39 and 40-49 to elicit information on shared beliefs, opinions, group concerns and expectations, group knowledge and awareness, and other normative perspectives. The primary objective is to elicit normative perceptions of circumcision, terminology used locally in discussing circumcision, and opinions regarding its acceptability as an HIV/STD preventive measure. Focus group discussions with community gatekeepers, stakeholders as well as community members were held.

*Qualitative data management and analyses* : The ethnographic data is transcribed and translated by experienced Rakai Health sciences program personnel. The word processed text is then coded and organized using NVIVO. Further editing is done in Uganda and the US. Data analysis/summaries will be completed in the US and will focus on barriers to circumcision, means for overcoming barriers, incentives that might be acceptable, and perceptions or beliefs which might lead to behavioral disinhibition.

# DATA COLLECTION, SITE MONITORING AND ADVERSE EXPERIENCE REPORTING

## Records to Be Kept.

Records for the Gates Trial are kept in secure filling cabinets, separate from the records of the NIH trial participants. The records to be kept for each subject are consent and information forms (I-V) and record forms 01-16. These are summarized in Tables 2.a. and 2.b. Files are maintained on each participant to provide a chronological record of all forms used. Files are organized by study number and maintained in locked cabinets in secure storage rooms.

## Data Management

- Data management is conducted by the Rakai Health Sciences Program and JHU. We have extensive experience managing large data sets. We are using existing Rakai Health Sciences Program census and survey forms, with supplementary record forms specific to the trial. Forms are translated into Luganda and printed in Uganda.

- All participants have a household census enumeration. Census files are used to generate updated data to provide computer generated tracking forms, and pre-printed labels for all questionnaires and samples.

- Data collection is conducted by highly trained and experienced full-time, same-sex interviewers. Field editors check all completed questionnaires and query inconsistencies or errors in the field so that interviewers can return to re-interview subjects and correct errors immediately.

- Subjects are identified by photo IDs and have permanent, unique alphanumeric check digit study ID numbers which prevent keying errors. Subjects enrolled from the cohort also have location numbers for community, household and census resident numbers to facilitate tracing.

- Data entry is done in Rakai Offices using Foxpro screens with programmed range and consistency checks. All lab data and questionnaires are double entered.. The data entry system is adapted for this trial by experienced data managers in Entebbe and JHU, and relational data bases will be constructed.

- The data is sent by FTP or zip files to JHU. Further data editing and cleaning are conducted at JHU and queries sent to Uganda for clarification and error correction.

- Security is maintained by storing all data files on codeword protected machines accessible only to senior data managers and analysts.

- A central manual of operations and standard operating procedures were written and updated as need arose.

- Adherence to protocol is assessed by periodic checks on consent forms and hard copy study instruments, as well as data sets.

## Clinical Site Monitoring and Record Availability

Monitoring will be conducted at intervals to be determined by the Gates Foundation and the DSMB.

All study hard copy records will be available to site monitors and will be maintained in individual chronological files on all study participants. Computer files may be accessed on site during a monitoring visit.

## Serious Adverse Event Reporting

The occurrence of severe adverse events (SAEs) is recorded at every post-randomization visit.

### General Definitions

An AE is defined as “any untoward medical occurrence in a participant and that does not necessarily have a causal relationship with the treatment. An AE can be any unfavorable and unintended sign (including an abnormal laboratory finding), symptom or disease temporarily associated with the beginning of the treatment (i.e., following randomization).**”** In this trial we recorded AES during or after screening.**”** Adverse events during screening and prior to randomization that result from study participation were also reported.

- The adverse events are defined in Table 3 and AE notification is given in form 11a.

### Severity of AEs

- The severity of AEs is characterized as:

. *mild* (transient and not affecting activity),

*. moderate* (requiring modification of activity or additional medical /minor surgical/intervention),

*. severe* (resulting in: death, life threatening condition, incapacitating symptoms which require bed rest, hospitalization, significant medical/surgical interventions or which result in persistent significant disability/incapacity).

- All severe occurrences are considered serious adverse events (SAEs) for reporting purposes, consistent with FDA definitions as follows:

. Life threatening: substantial risk of dying at the time of the AE

. Hospitalization (initial or prolonged): if admission to hospital or prolongation of hospital stay results from the AE.

. Disability: if the AE resulted in a significant, persistent or permanent change, impairment, damage or disruption in the patient’s body function/structure, physical activities or quality of life.

. Intervention required to prevent permanent impairment or damage: if it is suspected that the treatment may result in a condition which required medical or surgical intervention to preclude permanent impairment or damage to the patient.

### Relationship of AEs to the intervention

The relationship of AEs to study participation, particularly or medications will in most cases be clear. We use the following classification

. *definitely related* to study procedures such as surgery or anesthesia/analgesia (occurred during surgery or postoperatively),

. *probably related* to study procedures such as surgery or anesthesia/analgesia (occurred during surgery or postoperatively), but could arise from other causes,

. *possibly related* (occurred during participation, but could equally or more plausibly be ascribed to other causes), and

. *unrelated* (an event clearly explained by causes other than the trial participation ).

- The timing of AEs is divided into screening/enrollment/randomization, operative, postoperative and during longer-term follow up.

### Study Definition of Adverse Events

The anticipated adverse events are largely those associated with surgery or study medications. The description of anticipated potential AEs is given in Table 3. (This is in part based on the AE classification used for a NIH supported trial of male circumcision in Kisumu, Kenya). All medical officers are trained in the recognition and classification of the adverse events, and in the procedures required for reporting. The senior Medical Officers, is responsible for AE reports.

Any other serious events not defined in Table 3 are reported as SAEs.

**Table 3. Study Definition of Adverse Events**

| Adverse Event Type | Description of Adverse Event Type | Severity  and Codes |
| --- | --- | --- |

|  |  |  |
| --- | --- | --- |

| A. During Surgery |  |  |
| --- | --- | --- |

| Pain | Minor, not requiring additional anaesthesia | Mild (APS1) |
| --- | --- | --- |

|  | Moderate/severe controlled with additional anaesthesia* | Moderate (APS2) |
| --- | --- | --- |

|  | Severe, not controlled by additional anaesthesia | Severe (APS3) |
| --- | --- | --- |

| Excessive bleeding | More bleeding than usual, but easily controlled | Mild (ABL1) |
| --- | --- | --- |

|  | Bleeding that requires pressure dressing to control | Moderate (ABL2) |
| --- | --- | --- |

|  | Blood transfusion or transfer to another facility for management required | Severe (ABL3) |
| --- | --- | --- |

| Anesthetic-related event | Palpitations, vaso-vagal reaction or emesis | Mild (AAN1) |
| --- | --- | --- |

|  | Reaction to anesthetic requiring medical treatment in study clinic but not transfer to another facility | Moderate (AAN2) |
| --- | --- | --- |

|  | Anaphylaxis or any reaction requiring transfer to another facility | Severe (AAN3) |
| --- | --- | --- |

| Excessive skin removed | Adds time or material needs to the procedure, but does not result in any discernable adverse condition | Mild (AES1) |
| --- | --- | --- |
|  | Skin is tight, but additional operative work not necessary | Moderate (AES2) |

|  | Requires re-operation or transfer to another facility to correct the problem | Severe (AES3) |
| --- | --- | --- |

| Damage to the penis | Mild bruising or abrasion, not requiring treatment | Mild (ADP1) |
| --- | --- | --- |

|  | Bruise or abrasion to the glans or shaft of the penis requiring pressure dressing or additional surgery to control | Moderate (ADP2) |
| --- | --- | --- |

|  | Portion or all of the glans or shaft of the penis severed or burned by electrocautery | Severe (ADP3) |
| --- | --- | --- |

| B. First Month Post-Surgery |  |  |
| --- | --- | --- |

| Pain | Symptoms of pain requiring bed rest for less than half the day | Mild (BPA1) |
| --- | --- | --- |

|  | Pain requiring bed rest for more than half day | Moderate (BPA2) |
| --- | --- | --- |

|  | Excruciating pain requiring total bed rest | Severe |
| --- | --- | --- |

| Excessive bleeding | Dressing or other materials (for example underwear) spotted but dry | Mild (BBA1) |
| --- | --- | --- |

|  | Dressing or other materials wet with clotted blood requiring change of dressing | Moderate (BBA2) |
| --- | --- | --- |

|  | Dressing or other materials soaked with blood with obvious active bleeding requiring surgical exploration or transfusion | Severe (BBA3) |
| --- | --- | --- |

| Excessive skin removed | Client concerned but no discomfort on erection | Mild (BES1) |
| --- | --- | --- |

|  | Causes slight disconfort on erection but surgical correction not necessary. | Moderate (BES2) |
| --- | --- | --- |

|  | Interferes with life and surgical correction necessary | Severe (BES3) |
| --- | --- | --- |

| Insufficient skin removed | Prepuce partially covers the glans only when extended | Mild (BIS1) |
| --- | --- | --- |

|  | Prepuce still partially covers the glans and re-operation is required to correct | Moderate (BIS2) |
| --- | --- | --- |

|  | Not applicable |  |
| --- | --- | --- |

|  |  |  |
| --- | --- | --- |

| Swelling/Hematoma | More swelling than usual, but no treatment needed | Mild (BSH1) |
| --- | --- | --- |

|  | swelling requiring surgical exploration but no evidence of active bleeding | Moderate (BSH2) |
| --- | --- | --- |

|  | Rapidly expanding Heamatoma suggesting active bleeding requiring surgical exploration or referral | Severe (BSH3) |
| --- | --- | --- |

| Damage to the penis | Mild bruising or abrasion, not requiring treatment | Mild (BDP1) |
| --- | --- | --- |

|  | Bruise or abrasion to the glans or shaft of the penis requiring pressure dressing or additional surgery to control | Moderate (BDP2) |
| --- | --- | --- |

|  | Portion or all of the glans or shaft of the penis severed | Severe (BDP3) |
| --- | --- | --- |

| Infection | Pain and erythema with no obvious swelling | Mild (BIN1) |
| --- | --- | --- |

|  | Painful swelling with erythema or elevated temperature or purulent wound discharge | Moderate (BIN2) |
| --- | --- | --- |

|  | Cellulitis or wound necrosis | Severe (BIN3) |
| --- | --- | --- |

| Delayed wound healing | Healing takes longer than usual, but no extra treatment necessary | Mild (BDW1) |
| --- | --- | --- |

|  | Additional non-operative treatment required | Moderate (BDW2) |
| --- | --- | --- |

|  | Requires re-operation to correct | Severe (BDW3) |
| --- | --- | --- |

| Appearance | Client concerned, but no discernable deformity | Mild (BAP1) |
| --- | --- | --- |

|  | Minimal deformity does not require re-operation | Moderate (BAP2) |
| --- | --- | --- |

|  | Significant deformity requires re-operation to correct | Severe (BAP3) |
| --- | --- | --- |

| Problems with voiding | Transient complaint that resolves without treatment | Mild (BVO1) |
| --- | --- | --- |

|  | Requires a special return to the clinic, but no additional treatment required | Moderate (BVO2) |
| --- | --- | --- |

|  | Requires referral to another facility for management | Severe (BVO3) |
| --- | --- | --- |

| Wound dehiscence | Wound disruption involving no more than one principal suture. | Mild (BWD1) |
| --- | --- | --- |
|  | Wound disruption and involving two or more principal sutures. No surgical intervention required. | Modarete (BWD2) |
|  | Significant wound disruption requiring surgical correction | Severe (BWD3) |
|  |  |  |

| **C. One Month or More Post-Surgery** |  |  |
| --- | --- | --- |

| Infection** | Pain and erythema no obvious swelling | Mild (CIN1) |
| --- | --- | --- |

|  | Painful swelling/purulent wound discharge | Moderate (CIN2) |
| --- | --- | --- |

|  | Cellulitis or wound necrosis | Severe (CIN3) |
| --- | --- | --- |

| Delayed wound healing | Healing takes longer than usual, but no extra treatment necessary | Mild (CDW1) |
| --- | --- | --- |

|  | Additional non-operative treatment required | Moderate (CDW2) |
| --- | --- | --- |

|  | Requires re-operation to correct | Severe(CDW3) |
| --- | --- | --- |

| Appearance | Client concerned, but no discernable deformity | Mild (CAP1) |
| --- | --- | --- |

|  | Significant scarring or other cosmetic problem, but does not require re-operation | Moderate (CAP2) |
| --- | --- | --- |

|  | Requires re-operation to correct | Severe (CAP3) |
| --- | --- | --- |

| Excessive skin removed | Client concerned, but there is no deformity on erection | Mild (CES1) |
| --- | --- | --- |

|  | Causes slight discomfort on erection but surgical correction not necessary | Moderate (CES2) |
| --- | --- | --- |

|  | Interferes with sexual life and surgical correction is necessary | Severe (CES3) |
| --- | --- | --- |

| Insufficient skin removed | Prepuce partially covers the glans only when extended | Mild (CIS1) |
| --- | --- | --- |

|  | Prepuce still partially covers the glans and re-operation is required to correct | Moderate (CIS2) |
| --- | --- | --- |

|  | Not applicable |  |
| --- | --- | --- |

|  |  |  |
| --- | --- | --- |

|  |  |  |
| --- | --- | --- |

| Torsion of penis | Torsion is observable, but does not cause pain or discomfort. | Mild (CTP1) |
| --- | --- | --- |

|  | Causes mild pain or discomfort on erection, but additional operative work not necessary | Moderate (CTP2) |
| --- | --- | --- |

|  | Requires re-operation or transfer to another facility to correct the problem | Severe (CTP3) |
| --- | --- | --- |

|  |  |  |
| --- | --- | --- |

| Erectile dysfunction | Client reports occasional inability to have an erection | Mild (CED1) |
| --- | --- | --- |

|  | Client reports frequent inability to have an erection | Moderate (CED2) |
| --- | --- | --- |

|  | Client reports complete or near complete inability to have erections | Severe (CED3) |
| --- | --- | --- |

| Psycho-behavioral problems | Client reports mild sexual dissatisfaction attributed to circumcision, but no significant psycho-behavioural consequences | Mild (CPB1) |
| --- | --- | --- |

|  | Client reports significant sexual dissatisfaction attributed to circumcision, but no significant psycho-behavioural consequences | Moderate (CPB2) |
| --- | --- | --- |

|  | Significant depression or other psychological problems attributed by the participant to the circumcision | Severe (CPB3) |
| --- | --- | --- |

**COMMENTS:**

* Additional anesthesia implies a volume of local anesthetic in excess of initial amount drawn up in the syringe and calculated to be sufficient for patient’s weight before surgery.

** Infection is indicated by swelling, erythema, pain, elevated temperature (locally or systemically, or purulent discharge)

**New AE codes**

During the February 2006 review, it was decided that specificity could be improved by addition of new codes. This did not affect the diagnosis of AEs, but added more detail for future analyses. The additional codes were:

1. A new code for joint dehiscence (BWD) and infection (BIN) AEs, indicated by BWD/BIN. Degree of severity is per individual event and the first event to occur is reported first.

2. New codes for stich sinus: SS = Sinus at Suture site SS1 = Sinus with non-puss discharge SS2 = Sinus with purulent discharge. This would be included in infectious AEs, but adds specificity.

3. New code for anatomical site of lesion (F = Frenulum), because many wound dehiscence, poor skin apposition and infection involved the frenulum.

4. New codes for pain on intercourse: PI = Pain on Intercourse PI1 = No interference with sex, PI2 = Reduced sexual frequency, PI3 = Refrain from intercourse due to pain

5. A new code was added for allergic reaction to the iodine prep, since we observed cases of blisters following surgical site preparation

PREP REACTION APR 1 = MILD BLISTERS RESOLVED

APR 2 = MORE SEVERE BLISTERS REQUIRING TREATMENT

6. A number of cases of pain or wound dehiscence occurred after intercourse, so we coded intercourse related AEs: I = RELATIONSHIP TO INTERCOURSE

7. A number of cases of wound dehiscence were due to external trauma (e.g., heavy lifting, bicycle accidents) so this was coded as EX = EXTERNAL CAUSE

8. Code for stitch granuloma: SG=Stitch granuloma. CSG 1=Small capable of resolving,no need for surgical removal. CSG 2=Not capable of resolving, requires surgical intervention. CSG 3=Not applicable

No changes were made to the AE definitions, although with hindsight, we believe these could be improved for monitoring of control surgery related AEs.

### Management of AEs

- Free medical care is provided for AEs related to the procedure, including surgical care if needed. If referral for medical or surgical complications is required, participants are referred to the Kalisizo Hospital (2-3 minutes driving time), the Rakai Hospital or hospitals in the neighboring District of Masaka (approximately 30 minutes driving time).

### Reporting of AEs

- All AEs are ascertained and documented by Program medical officers

- All deaths and SAEs are notified to the Ugandan and U.S. PIs within 48 hours, by e-mail or fax.

- The U.S. and Ugandan PIs then notifies the relevant IRBs (Johns Hopkins, Columbia University and Uganda Virus research Institute) in writing as soon as possible, and not later than 10 days after identification of the SAE. The nature, severity, timing and relationship of the AE to circumcision or study medications is specified by the medical officer. A summary of how the severity and relationship to study participation was determined is provided by the PI, and clarifications are made by phone with the medical officer as needed.

.

### Tabulations of AEs

- The PI will submit aggregate reports of all AEs and tabulations via the Program Statisitician to the DSMB. AEs will be described by type, time since surgery or medications, severity and relationship to study treatments. Dates of AEs are also tabulated to assess temporal trends or patterns.

- AEs are tabulated by total number, and by number of AEs per individual for those participants experiencing more than one AE**.**

### Source documentation

**-** Operative and postoperative AEs use Rakai Health Sciences Program documentation (Forms 11)

- Medical visits to health centers or other facilities outside of the Rakai Health Sciences Program cannot be completely documented due to poor records. We endeavor to obtain copies of hospital medical records where feasible, after obtaining permission from the index person. Rakai Health Sciences Program reports (such as questionnaires), are used to provide information.

- To maximize documentation Rakai Health Sciences Program offers free medical care or referral for injury or illnesses as a result of study participation. Phone numbers of medical officers are provided on the Postoperative patient Information Sheet (Form 46), we provide transport if needed and reimburse participants for transport costs. In addition, we arrange community health workers to provide a contact person in each village who can facilitate contact with the Project. Records are maintained for unscheduled visits (Form 16), and an AE report completed if indicated**.**

# STATISTICAL CONSIDERATIONS

## Preamble

There are several trial end points including:

- circumcision safety

- circumcision acceptability

- circumcision efficacy for prevention of HIV acquisition in HIV-negative men

- circumcision efficacy for prevention of STD acquisition in HIV-negative and HIV-positive men

- circumcision efficacy for prevention of male-to-female HIV and STD infections

- the effects of circumcision on behavioral disinhibition.

As noted previously this Gates supported study is an individually randomized, unblinded trial of male circumcision in ~ 800 HIV-positive men and~ 1000 HIV-negative men who decline VCT, to be conducted in conjunction with a NIH supported trial of 5000 HIV-negative men who accept VCT. The sample size estimates for safety and acceptability of circumcision are based on the number of men enrolled in the Gates trial. The sample size for the end point of HIV acquisition in circumcised and uncircumcised HIV-negative men is based on the combined Gates (n ~ 1000) and NIH (n ~ 5000) populations. The NIH trial is independently powered for an HIV incident end point and the 1000 HIV- men contributed by the Gates trial component enhance this power and allow analyses of subgroups. However, the 1000 men enrolled in the Gates trial will not provide power for an independent HIV end point.

The end points of HIV transmission from HIV-positive men to HIV uninfected women in relationships with circumcised and uncircumcised men, are based on the population of HIV+ men enrolled in the Gates trial, and anticipated HIV seroconverters from both the NIH and Gates trials.

### Initial Assessment of Safety.

We initially assessed of safety after completing the first 100 circumcision patients who received intensive postoperative follow up . This was followed by a pause in enrollment to evaluate safety.

The frequency of complications following adult circumcision in the Rakai trials is approximately 3.6%. However, review of the surgical literature suggests that complications occur in approximately 2% of patients, and what we define as moderate or severe AEs occur in approximately <0.5% of surgeries. The literature is based on clinical case reports, and the intensity of ascertainment or duration of observation cannot be fully assessed. It is possible that the intense surveillance and careful definition and probing for complications in this research study may differ from these case series, and that our estimates of frequency of events, especially minor AEs which are more likely to detected by intensive surveillance may be incorrect (i.e., we may observe a higher frequency than that reported in the surgical literature as a result of more intensive ascertainment and definitions of minor events**).**

Our initial assessment of safety was based on the first 100 circumcisions performed. Assuming that the expected number of AEs were < 2%, and that moderate and severe AEs were < 0.5% (based on limited data from surgical case series), we used StatXact to construct exact 95% confidence intervals and exact one-sided p-values for a **binomial proportion with N =** 100, to compare the number of observed AEs against two “null” hypotheses; a) that all AEs will be < 2.0% and b) that moderate/severe AEs will be < 0.5%. These estimates are shown in Table 4.

There were less than six AEs observed in Stage 1, the 95%CI still contained 2%, compatible with the anticipated rate, and the study proceeded as planned. (Note: the DSMBs reviewed AEs in Stage 1 and agreed that the trials could proceed.)

As noted above, intensive postoperative surveillance might yield higher rates of minor AEs, relative to those reported in routine case series. Therefore, it is necessary to carefully consider the severity of AEs observed, before making a judgement on whether observed AEs exceed the expected numbers.

### Ongoing Monitoring of Circumcision Safety During the Trials

Please note, these estimates are based on the total populations of HIV- men enrolled in the Gates and NIH trials

Surgical and postoperative complications were expected to be ~2%, and were monitored continuously during stage one and periodically in Stage 2 (at approximately 4-7 months, ~20 and ~40 months) to ensure safety. Monitoring of the rate of complications associated with circumcision is done using similar techniques described for monitoring efficacy. To facilitate the proper analysis of this continuous safety data, we propose to use the methods of Lan, Rosenberger, and Lachin in which they demonstrate how to construct a sequential boundary (Wald boundary) for a Z‑statistic used to continuously monitoring data that maintains an overall Type I error rate. The methods build upon the spending‑function methods of Lan & DeMets, and can also be used when switching from occasional (e.g. group sequential) monitoring to continuous monitoring.

We planned to conduct interim analyses if required for DSMB safety assessment at 20 and 32 months. We expected to have completed 1,500 circumcisions by 20 months and 3,000 by 32 months. Using StatXact (Cytel Software Corp), we estimated the smallest number of AEs that would give an exact 1-sided 95% lower confidence bound on the estimated rates of AEs (not adjusted for interim analyses). With 1,250 men circumcised by 20 months, if the total AE rate  2.64% (33 events), the lower 95% CI bound would significantly exceed the expected rate of 2.0% (25 events), and for moderate/serious AEs, a lower 95% CI bound of an observed rate of 0.88% (11 events) would significantly exceed the expected rate of 0.5% (7 events). Similarly, at 32 months with 2,500 surgeries completed, if the total AE rate  2.48% (62 events), the lower 95% CI would exceed the expected 2.0% rate (50 events), and for moderate/serious AEs, with an observed rate of 0.72% (20 events), the lower 95% CI would exceed the expected rate of 0.5% (13 events)

## Primary Endpoint: Male HIV Incidence.

As noted above, the power to detect the efficacy of circumcision on male HIV acquisition is based on the total HIV-negative population of ~ 6,000 men, comprised of ~ 1,000 HIV-negative men in the Gates trial and 5000 men enrolled in the NIH trial. We present here the estimates for this population

***Efficacy:***

The Rakai STD Control Study^78^ found an incidence of 1.8/100 PY among the uncircumcised men and 0.9/100 PY among the circumcised men, with a Poisson adjusted RR = 0.53.^1^ We base our sample size estimates on this efficacy, and use a two-sided test with  = 0.05 and 1- = 0.80.

***Assumptions:***

We assumed two years follow up, with participants enrolled between 9-26 months of the study and follow up completed by study month 50, and an annual loss to follow up of 15% (please see feasibility). We also estimated the loss of power due to possible contamination (i.e., cross-over) of 7.5-10%.

***Allowance for contamination or reduced magnitude of anticipated efficacy:***

We previously observed a RR = 0.53 for HIV incidence in circumcised relative to uncircumcised men, and powered the study to be able to detect a lower efficacy of RR = 0.57. We also assessed potential effects of contamination as follows: Let RR* be the observed rate ratio in the presence of cross-over (p), and let RR be the true rate ratio in the absence of contamination. Then RR* = (p + [1-p] x RR)/(p x RR + [1-p]) = RR/([p x RR] + [1-p]) (J. Hughes , pc). With p = 0.075 (i.e., 7.5% contamination), the observed RR* = 0.55. The sample size needs to be increased to account for this cross-over effect. With 2,500 men enrolled per arm we would accumulate 2,125 py in the 1^st^ follow up year and 1,806 py in the 2^nd^ follow up year, with a total over two years follow up = 3,932 py, which could detect a RR* ~ 0.57 with 80% power if the true RR = 0.53. (Please note this is 18.4% larger than the 3,209 py needed in the absence of contamination). Therefore, we enrolled ~ 2,500 men per arm into the NIH trial providing 3,932 py per arm.

***Allowance for data dependent stopping:***

We propose using formal interim analysis methods for monitoring the primary outcome (incidence of HIV infection) as well as the safety aspect of the study. Lan‑DeMets group sequential methods with O'Brien‑Fleming bounds ^117,118^ will be utilized to ensure the maintenance of an overall Type I error rate of 5%. For monitoring HIV incidence, we anticipate one interim analysis to allow DSMB to decide whether we can commence circumcisions in the control arm, and allow sufficient time for completion of control surgeries before the end of the project. Assuming an interim analysis at project month 40, we will accrue a total of 3,028 py observation per arm, which represents 77% (3028 * 2 / 3932 * 2) of the information to be accumulated during the trial. This information fraction, for example, will give sequential O'Brien‑Fleming boundaries equal to 2.303 for the interim analysis and 2.017 for the final analysis. Without using these boundaries, we estimate (via simulation with 10,000 runs) that the overall Type I error rate wouldl be inflated to approximately 7.7%. Using these boundaries, we estimate that the Type I error rate is maintained at 0.05 and that the power for a RR of 0.53 is equal to 89.5%. Furthermore, with this RR, we estimate that there is approximately a 70% chance of crossing the boundary at the interim analysis. Sequential confidence intervals ^118a^ will be used when reporting results. The investigators will be blinded for the interim analysis. Monitoring of safety will be done similarly, except that it will occur on a continuous basis, and will use the methods of Lan *et al.*

***Age criteria and selection of high risk groups:***

Rakai has is a mature generalized epidemic setting with relatively high incidence throughout the population. Table 5 shows male age-specific HIV incidence. Incidence is lowest among adolescents, high and sustained among men aged 20-29, and declines modestly at older ages. Exclusion of adolescent men 15-19, would exclude a group with considerable HIV incidence and which is of great importance in future programs. Restricting enrollment to the highest risk age group (20-29 years) would marginally increase incidence relative to the total male population 15-49, but would only include 39.5% of all men. Enrollment restricted to men aged 20-29 would require adding many more communities in order to maintain power, but this complicates logistics and inflates costs. Moreover, acceptability was highest (65% in ages 20-29), so self-selective acceptability would automatically over sample these highest risk age groups. We therefore proposed to enroll all consenting men aged 15-49.

***Additional Power provided by the ~ 1,000 HIV-negative men who decline VCT, enrolled in the Gates sponsored trial and approximately 500 additional HIV-negative men enrolled into the Gates trial after NIH trial enrolment is completed.***

Prior analyses have shown that persons who refuse VCT have higher risk behaviors than persons who accept VCT, and that this difference in risk profile is reflected in higher HIV incidence among the former populations. (HIV incidence was 1.5/100 py in men declining VCT versus 1.3/100 py in men accepting VCT.

We estimated that about 400 HIV-negative men would decline VCT per arm, assuming the loss to follow up and cross over rates above, we anticipated that these men would add an additional ~ 1256 py per arm, to the person time contributed by the HIV- men enrolled in the NIH trial. Thus, the total person years observation contributed by the combined populations of HIV-negative men from the two trials is 5188 py (1256 + 3,932 py per arm). This would enhance the study power to detect a lesser efficacy of circumcision, and help guard against loss of power if the assumptions regarding follow up and cross-over prove to be too conservative.

## Male STDs and STD Symptoms:

These estimates are based on the total populations of HIV- negative men enrolled in the Gates and NIH trials.

*STDs:*

All participants will be tested for HIV and syphilis at all study rounds. For budgetary reasons, additional specimens will be collected for selective STD testing with other funds. (Please see laboratory methods). From prior data the expected infection rates are: syphilis prevalence ~10% and incidence 2.2/100 py. The RR for syphilis in circumcised versus uncircumcised men was 0.71. With 5492 observations per arm, we have >80% power to detect a RR of 0.70 for the effects of circumcision on syphilis prevalence, and >80% to detect a RR = 0.5 in syphilis incidence.

***STD symptoms:***

The RR for GUD in HIV-negative circumcised versus uncircumcised men was 0.70. With 2,500 men enrolled per arm in the NIH funded study, and approximately 1,100 per arm in the Gates-funded study, and 85% follow up, we have 85% power to detect a difference of this magnitude. The RR of balanitis in all circumcised versus uncircumcised men was 0.53 (prevalence 11.6% in circumcised and 22.8% in uncircumcised), and we have 98% power to detect an effect of this magnitude. Therefore, these sample sizes are adequate, even with cross-over effects.

Thus, the study is adequately powered to detect reductions in STDs and STD symptoms in the circumcision relative to the control arm.

## Female HIV Acquisition*.*

Information on women will be derived from the RCSS surveillance surveys, and women are not enrolled in the male circumcision trials. Female HIV acquisition associated with the male partner’s circumcision status will be assessed among HIV-negative women in relationships with HIV-positive men. Please note, we do not enroll HIV-discordant couples; the men are individually enrolled in the circumcision trials and the women individually enrolled in the RCSS. Couples linkage will only be done retrospectively after completion of the trials, although promotion of couples VCT will be ongoing throughout the study. The information on male partner circumcision and HIV status will be derived from this retrospective linkage.

Our observational data in HIV-discordant couples found a male-to-female transmission incidence of 5.2/100 py if the male was circumcised and 13.2/100py if the male was uncircumcised (RR = 0.39). (Please note, we strongly promote condoms and couples counseling, but acceptance of these services within married couples is low). We estimate sample sizes required to detect an efficacy of this magnitude, with  = 0.05 and 1- = 0.80. The person years accrued for female exposure in discordant couples with an HIV+ male partner can be estimated from the number of prevalent and incident discordant couples with HIV+ men, as follows:

***i.*** *Person years contributed by prevalent HIV+positive men in discordant relationships with HIV- negative women:* We estimate that 500 HIV positive males per arm will accept enrollment . From prior data, 80% of men can be linked to a female partner and 55% of prevalent HIVpositive men have HIV-negative female partners. Thus we expect 220 HIV prevalent males in a discordant relationship (i.e., 500 x 0.8 x 0.55). Assuming

85% annual follow up, we estimate that 220 HIV-discordant, male HIV+/female HIV- couples, will provide 186 py in the 1^st^ follow up year and 158 py in the 2^nd^ follow up year, yielding a total of 344 person years in exposed, initially HIV- women. With 344 py per arm, will have 85% power to detect a RR = 0.39. Assuming 7.5% contamination, the sample size is sufficient to detect a RR* of 0.41 with 80% power.

***ii. Sample size for female acquisition after stratification by male HIV viral load*:**

We observed no seroconversions among women if their HIV+ male partner’s viral load was below 50,000 copies per mL and the HIV-positive male was circumcised, whereas among couples with HIV-infected uncircumcised men, the incidence rate was 9.6/100 py. Thus, stratifying by the viral load of HIV-positive males we will be able to detect an effect of this magnitude with 110 py, per arm ( = 0.05 and 1- = 0.80. In our study, 87.1 % of infected men in discordant relationships had viral loads < 50,000 copies per mL, so in this subgroup we expect to observe ~ 239 py in each arm. Therefore, the study has 98% power to detect an effect of circumcision in men with HIV-1 viral loads <50,000 cps/mL. Since this is an *a priori* hypothesis, based on observational data, such subgroup analysis is justified.

## Behavioral disinhibition:

These estimates are based on the total populations of HIV- men enrolled in the Gates and NIH trials

From prior data we expect ~ 25% of men to have multiple sex partners, ~ 17.0% to use condoms inconsistently, ~ 5.0% to consistently use condoms, ~ 46.0% report alcohol consumption in the previous 7 days and ~42% report alcohol consumption with sex. These behaviors have been shown to be significantly associated with HIV risk in the Rakai population. Assuming these rates pertain in the control arm, we will have 80% power to detect the following markers of behavioral disinhibition in the intervention arm: a) an increase in multiple sex partners to 30%, b) a reduction of inconsistent condom use to 13.2%, and c) a reduction in consistent condom use to 3% (i.e., an overall reduction in condom use of - 5.8%), and d) and an increase in alcohol use within the past 7 days to 50.4%, or of alcohol consumption with sex to 46.4%.

We will also assess behaviors among the ~1000 men who decline VCT, compared to the men who accept VCT, to determine whether VCT acceptance is associated with lower risk behaviors.

## Accrual and Feasibility:

The average number of men aged 15-49 per community is ~ 190, of whom we expect 137 to be uncircumcised and HIV-negative (84% uncircumcised and 86% HIV-).^2^ Assuming 50% acceptance of circumcision we expect an average of 69 subjects enrolled per community. We enrolled male participants from RCSS communities and allowed non-cohort participants to enroll as “walk in subjects”. We believed that the current cohort surveillance participants and walk in participants will readily achieve the sample size goals. (Our previous trials enrolled over 12,000 subjects).

***Feasibility of follow up:***

In the present trial, the eligibility criteria include intent to remain in the community for at least one year. We will implement additional procedures for active tracing. In brief, we will a) ask relatives of absent subjects to contact them or to provide contact addresses, b) ask absentees to return for follow up and compensate them for time and travel expenses, and c) establish peripheral hubs in urban centers such as Masaka and Kampala where most absentees go for work or education, in order to facilitate follow up outside the District. With eligibility based on intent to stay and active tracing, we believe that we can achieve a minimum of 85% or more follow up. In our maternal-infant trial we achieved 94% follow up of 4,036 persons, and in our MER study we have achieved 95.4% follow up on a sample of 383 subjects. We therefore believed follow up of 85% was feasible in the proposed trial.

## Randomization and Stratification

- Johns Hopkins generated a list of randomized numbers in blocks of 20 to insure comparability of men randomized to intervention and control arms, within cohort communities or group of “walk in” participants”.

- A random assignment sheet was placed in an opaque sealed envelope. The envelopes in blocks of 20, were generated by Rakai Health Sciences Program Data management using a computerized list provided by JHU, and were retained securely by the Circumcision Data Management Team.

- After enrollment and consent, men were asked to select an envelope from the block of 20 envelopes being used at that time. The envelope contained the assignment sheet showing the randomization number and random assignment to treatment or control arm. The Enrollment Team then affixed the label with the individual’s study ID number to the sheet. (Form 18).

- The assignment sheet with the study ID# labeled was entered into a data base to record study arm allocation.

- The list of random allocation numbers linked to participants was retained at JHU in locked files, and in password protected computers.

- All randomization envelopes were logged out and in daily. A data base monitored used and unused allocation numbers, and tracked disposition of all envelopes.

- Men were randomized at the enrollment visit. If, participants change their minds and withdrew, or at a subsequent preoperative visit, the surgeon determined that circumcision was contraindicated for medical reasons, the men were considered enrolled and randomized.

## Analysis Plan

### Primary Endpoint, HIV incidence in Males.

This analysis will be done separately for HIV-negative men enrolled in the Gates and NIH trials, and for the combined population of HIV-negative males enrolled in both trials.

HIV incidence per 100 person-years (PY) will be estimated, assuming seroconversion occurred at the mid-point of each follow up interval. In both arms, time from enrollment will be accumulated up to 24 months of follow up. Person time will discount the postoperative period of sexual abstinence for intervention arm subjects. Baseline samples of subjects who seroconvert during the first follow up interval will be tested by detuned EIA and PCR to detect prevalent infections during the window period, and any suspected prevalent cases will be excluded from incidence analyses.

Exploratory data analysis will examine the populations in the two arms at enrollment to assess baseline comparability. HIV incidence will be determined by strata of covariates such as age, marital status, behaviors (e.g., number of partners, condom use), health status and other variables to identify potential confounders. We will also assess contamination (i.e., cross-over) among men allocated to circumcision who do not receive surgery, and among control subjects who opt to have circumcisions performed from non-Project services.

An intent-to-treat approach will be taken, and all men who enroll will be included, irrespective of whether intervention arm participants ultimately receive circumcision, or whether control arm participants crossover and receive circumcision prior to 24 months follow up. The rationale is that a proportion of enrolled men who initially accept circumcision may change their mind prior to receipt of surgery, and such men are likely to be self-selected. Because circumcision is delayed in the control arm, such self-selection could be differential between randomization groups. Therefore, in the circumcision arm, enrolled men who subsequently refuse circumcision, will be ascribed half the person time from enrollment to scheduled date of circumcision.

We will assess HIV incidence in enrolled HIV-negative men. The primary analytical model that will be used will be Poisson regression to compare the HIV incidence rates between the two study arms.^113^ The relative incidence of HIV in the circumcision arm versus the control arm, will be estimated using Poisson regression models with adjustment for individual-level variables found to differ between groups at enrollment, and for potential confounding variables. Potential confounders determined from prior risk analyses will include age, marital status, risk behaviors (i.e, number and nature of partners, condom use and alcohol use), or imbalance of covariates between study arms will be used for adjustment in the primary models. Secondary analyses will include any additional potential confounders identified during exploratory data analysis and will include covariates associated with HIV incidence (p < 0.15), or covariates with an incidence RR > 2.0. Poisson multivariate models will be fit for the whole population and for strata of particular interest (e.g., by age, sexual risk behaviors etc.). Although we are doing an individually randomized trial, the structure and logistics of field work requires that men be randomized within communities. Thus, we will also fit Poisson models with random effect terms for each community, to insure adjustment for clustering in HIV incidence.

We will also assess the associations between selected STDs and STD symptoms and incident HIV infection to determine whether effects of circumcision on HIV incidence may be mediated by STD cofactors. If, as expected from prior analyses, selected STDs and STD symptoms such as GUD are associated with incident HIV, we will assess the circumcision effects on HIV incidence using stratified models or via interaction terms.

### Female HIV Acquisition

We propose an intent-to-treat analysis . The unit of analysis is women in relationships with HIV-positive male partners. Although the male’s circumcision status can be ascertained by interview with the female partner, the male’s HIV status can only be determined by linking couples from within the RCSS. This will be done at the conclusion of follow up. The linkage will allow identification of men enrolled in the circumcision trials, determine their randomization arm and their HIV status, and provide an objective measure of circumcision status as well as the timing of the circumcision.

We will determine male-to-female transmission (female HIV incidence per 100 py) in the circumcision arm relative to control using multivariate Poisson regression as described for male HIV acquisition in 8.9.a above.

Data are also collected on the frequency of intercourse, so in addition, we will estimate the probability of HIV infection per coital act among circumcised and non-circumcised men using published methods.^20,116^ The probability of transmission in the i^th^ couple (P_i_) can be written as 1- (1-) ^n^ _i_, where n_i_ is the number of acts during the follow up period, and the infectivity () is the probability of transmission per act.^116^ Allowing the infectivity to depend on covariates through a complementary log-log link, leads to the regression model [log -log (1-P)] = log n_i_ + b_0_ + b_1_X_1_ where b_1_ and X_1_ are vectors of regression coefficients and covariates, respectively. This model will be fit using statistical methods for generalized linear models, specifically binomial regression with a complementary log-log link and offset term log n_i_ (SAS Institute Inc., Cary, NC.). The comparisons will again be between circumcision arm and control. We have shown that the probability of male-to-female HIV transmission per coital act is 0.0004 if the male partner was circumcised and 0.0015 if the male was uncircumcised (RR = 0.27, unpublished).

We observed no seroconversions among women whose circumcised male partner’s viral load was below 50,000 copies per mL, whereas in uncircumcised men with viral loads <50,000 cps/ml, the female incidence rate was 9.6/100 py. In our couples study, 87.1 % of infected men in discordant relationships had viral loads < 50,000 copies per mL. Therefore, we examine the efficacy of male circumcision in prevention of female HIV acquisition, by strata of male HIV-positive partner viral loads. Since this is an *a priori* hypothesis, based on observational data, such subgroup analysis is justified.

### Secondary Endpoints:

***STD Prevalence and GUD.***

This will be assessed in both male trial participants and in female RCSS participants.

Prevalent syphilis and STD symptoms (GUD, discharge/dysuria) at baseline will be compared between study arms. At follow up visits 1 and 2, we will determine the period prevalence of STD symptoms and cumulative prevalence of syphilis (the latter based on prevalence because of persistence in seropositivity). In persons free of initial serologic infection at baseline, incident syphilis will be derived, and covariate adjusted rate ratios of incident STD symptom endpoints will be estimated by Poisson regression modeling as described above.

***Behavioral change (disinhibition).***

This will be assessed in both male trial participants and in female RCSS participants.

To assess possible behavioral disinhibition we will determine the frequency and changes in reported frequency of risk behaviors (e.g., number, type of partners, condom use, alcohol use) by study Arm, at enrollment and at each follow up. Data will be displayed graphically for each follow up visit. We will estimate the adjusted odds ratios of risk behaviors in the intervention versus the control arms using multivariate logistic regression for each survey visit. We will assess intra-individual changes in risk behaviors as follows. For continuous variables (e.g., number of partners), the intra‑individual change in risk behaviors from enrollment will be determined, and the mean within‑individual behavioral change between study arms will be assessed using repeated measures ANOVA or paired t tests. For dichotomous variables, we will assess the change in risk behavior over time, and estimate adjusted odds ratios of risk behavioral change between study arms by logistic multivariate regression. For categorical behavioral outcomes (e.g., non use of condoms, inconsistent and consistent use) we will use multivariate polychotomous logistic regression. In addition, we will have data from ongoing cohort surveillance to measure behavioral trends among non-participants, and we will determine behavioral change in the non-trial population in order to assess general trends, and to compare such non-trial associated behaviors with those reported by participants in both arms. A composite risk score will be developed on the basis of these analyses.

The relevance of changes in risk behaviors with respect to HIV incidence will be determined from prior Rakai studies of risk factors for HIV acquisition and from epidemiologic data generated by the circumcision trial. The behaviors specified above have been shown to be associated with a risk (rate ratio) ~ 2.0 or more in our observational analyses. We will estimate the effect on HIV incidence by study arm, of observed behavioral disinhibition (should it occur), using stratified analyses and multivariate analyses and stochastic modeling.

## Interim Data and Safety Monitoring

It was suggested that DSMB review accrual of data at ~ 20 months (accrual of 2870 subjects), 31 months at which time all subjects will have been enrolled, and two year data would be available for the first 200 participants, and at month 40 when 2-year follow up data would be available on 80% of volunteers.

The DSMB monitored the ethical conduct of the study and evaluated accumulating data for adverse and beneficial treatment effects. We requested Ugandan representation to address specific cultural, religious and other issues. Dr. B. Lo, Director of the Program on Bioethics at UCSF, and member of the National Bioethics Advisory Commission (NBAC), assists us with ethical issues. At the first meeting, the design and methods for conducting the pilot study was reviewed, and criteria developed for evaluating the pilot study to determine whether to initiate the clinical trial. We also proposed that the DSMB considers the following criteria for continuation of the randomized trial:

***Safety.*** The expected rate of total AEs following circumcision was expected to be ~ 2%. This was continuously monitored during the trial and specifically assessed by the DSMB after the first 100 surgeries, and at 20 and 31 and 40 months.

***Acceptability.*** Acceptability of circumcision should be about 50% among eligible men. If acceptance is less, the DSMB will evaluate the data from the pilot study to determine whether to proceed with the full-scale randomized clinical trial, to change accrual methods or to propose an alternative design.

**Stopping rules: The following stopping rules are suggested:**

***Efficacy Male HIV Acquisition.*** We proposed an interim analysis at 40 months. This was to include men enrolled in both the NIH and Gates trials. If HIV incidence in one arm significantly exceeds that of the other arm, it was proposed that the trial be discontinued following DSMB review and discussion with the Gates Foundation. We suggested that formal statistical monitoring methods such as the Lan-DeMets group sequential approach ^118^ with an O’Brien-Fleming type spending function. These methods minimize the chance of inappropriately terminating the trial in its early stages. If interim analyses suggest equivalence of HIV incidence in the two arms, conditional power methods will be used to estimate the chance that further data accumulation will result in significant differences. If the probability is low, consideration will be given to stopping the trial.

Please note, the female acquisition end point cannot be determined prior to the completion of the trial

***Behavioral changes, STDs, and disinhibition.*** It is possible that, despite intensive health education, circumcision may lead to false expectations of protection, engendering increased risk behaviors. If there is evidence of increased risk behaviors we would intensify health education efforts. However, if such improved education is unsuccessful, we suggest establishing stopping rules using the following possible measures of disinhibition in the intervention versus the control arm: a) a significant decline in the frequency or consistency of condom use, b) a significant increase in the number of extramarital partners, c) a significant increase in use of alcohol with sex, d) a significant increase in HIV incidence or selected STDs which are markers for risk behaviors (e.g., incident syphilis).

# RAKAI COMMUNITY COHORT STUDY (RCCS)

## Populations

The populations for which funding is provided for follow up via the Gates Foundation Grant included ~ 6000 women aged 15-49, and ~ 3000 men who decline enrollment in the circumcision trials sponsored by Gates and NIH. These individuals will be enrolled in the RCCS, and will be asked to provide informed consent using the already approved RCCS consent forms. They will be asked to provide interview information and samples conventionally obtained in the RCCS surveys, and there will be no additional burden or risk associated with the separate circumcision trials.

## Inclusion Criteria for the RCCS Populations

- Residents of cohort communities

- Aged 15-49 at screening

- are capable of and provide informed consent

- are not enrolled in either the Gates or NIH supported circumcision trials

## Brief description of the cohort surveillance

This is an open cohort that has conducted annual surveys of persons resident in the cohort communities who consent to cohort participation. The cohort consent forms are appended, and these have been approved by all IRBs.

Consenting individuals complete an interview at enrollment and at each follow up to ascertain sociodemographic characteristics, behaviors (e.g., numbers and types of sexual partners, sexual networks, condom use, alcohol use sex, coercive sex and domestic violence), genital hygiene, health status (symptomatology suggestive of STDs or AIDS, use of health care services), use of VCT and family planning. Partner information is obtained via questions on sexual networks (up to 4 partners within the past year) and by linkage information that allows identification of marital or consensual partners enrolled in RCCS.

Serum samples are obtained for serology (HIV and syphilis) and aliquots are stored for future testing. Women are asked to provide self-collected vaginal swabs for detection of trichomonas, BV, HPV, gonorrhea and chlamydia stored for future testing. Persons with symptomatic GUD are asked for an ulcer swab for Multiplex PCR detection of infections such as HSV-2, syphilis and chancroid, and stored for future testing.

Please note, with the exception of HIV and syphilis serology, none of these tests can be run in real time and used for therapeutic purposes.

## Enrollment of women who are not RCCS participants

Partners of “walk in” male trial participants who are not enrolled in RCCS, were invited to enroll. Men were given a “Spouse referral chit” to give to their wives/partners, and women were given an invitation to participate in RCCS. If they agreed to do so, they were consented using RCCS consent forms.

## Utilization of information from the RCCS

Data on female RCCS participants will be used to assess the efficacy of male circumcision for prevention female HIV/STD acquisition, and disinhibition, as described in 8.9.b and c. above.

Data from the ~3000 male RCCS participants who are not enrolled in the circumcision trial will contribute to assessment of behaviors and SRs/HIV among the general male population.

# HUMAN SUBJECTS

We will first describe services provided by the Rakai Health sciences Program and relevant actions as background to consideration of human subject issues**.**

## Services of relevance to Human Subject Considerations

**Community Advisory Board (CAB)**

The CAB reviewed the proposed trial and their letter of approval and list of members is attached. The CAB recommended that efforts be made to promote condom use among circumcised men, that well fitting garments and transport be provided to men immediately after circumcision if required, and that compensation be offered ($15.00) for lost time following surgery. These recommendations are incorporated into the proposal. The CAB recommended that men over age 49 be provided with circumcision if they request the procedure, and subject to DSMB approval, we will make this service available after completion of the trial. The CAB did not express reservations about the delayed circumcision provided in the control arm, nor about randomization. With assistance of the CAB and our community mobilization team , we will establish liaison with Christians and animist religious leaders, to address any concerns about circumcision.

**Condom Promotion:**

Condoms are provided free to all enrolled men who are advised to practice consistent use, and to obtain re-supplies from community depots maintained by the Project. Circumcised men are advised to only resume intercourse after certified wound healing and to practice consistent condom use. Current condom use has increased from 9.9% in 1995 to > 30% in 2001. Use with extramarital partners was 30.8% in 1995 and is currently over 60%. Thus, the Rakai Health sciences Program has markedly increased condom use by the community-based education and distribution program. However, despite repeated intensive Rakai Health sciences Program condom promotion via community meetings, counseling, and community distribution of supplies, consistent condom use remains low (~ 8 % in 2001), hence HIV incidence is stable at around 1.4/100py, and there is a need for innovative prevention interventions.

**Voluntary HIV Counseling and Testing (VCT):**

The Project strongly promotes and provides free VCT on request to individuals or couples, and VCT is encouraged at community meetings and community counseling sessions. Ready access to VCT is provided by trained full-time counselors resident in the communities and 2 counseling supervisors. VCT is provided in the home or at the counselors office, at the client’s discretion. Pre- and post-test counseling and long-term counseling support are available.^83^ The counseling messages include information on HIV/STD transmission and prevention including fidelity, abstinence, condom use and other safe sex behaviors, partner notification and couples counseling, and general health. Post-test counseling includes interpretation of HIV results, prevention of transmission/acquisition and support. We adhere to Ugandan Government policies on VCT^83b^ which explicitly require that VCT be voluntary and preclude disclosure of results to third parties, including sexual partners, without the written permission of the index individual.

In 1994 only 10% of Rakai community cohort participants accepted VCT. By 2002 over 80% of the Rakai cohort have received VCT, and 33% accept couples counseling. We believe these are the highest rate of VCT acceptance in any general African population (and among the highest documented rate achieved for any general population in the world).

**General medical Care and Health Education.**

All trial participants have access to free general health care provided by Rakai Health sciences Program at time of scheduled contact. Between surveys, care is available via the Project’s fixed clinic in Kalisizo and Government health services. The Project provides free health education, condom promotion and free condoms to all residents (participants and non-participants).

## Institutional Review Board (IRB) Reviews.

The Gates sponsored study was reviewed and approved by the Scientific and Ethical Committee (SEC) of the Ugandan Virus Research Institute (FWA 00001254, expiry 8/30/04, final approval was anticipated by 1/23/2003), and Johns Hopkins University Committee for Human Research (approved 1/08/2003, CHR# H.32.02.05.10.B). We are collaborating with Dr. Bernard Lo, Bioethicist, who will advise us regarding resolution of ethical questions throughout the trial. We get annual continuing review approvals from SEC and WIRB.

## Training in Research Ethics:

All Rakai Health sciences Program senior investigators in the US and Uganda, have taken and passed courses in Research Ethics/Good Clinical Practice. All other Ugandan project personnel having contact with participants, participant data or laboratory samples, have received research ethics training in Uganda, based on materials from Johns Hopkins and Columbia.

## Proposed involvement of Human Subjects:

The trial participants will be 1000 uncircumcised adolescent and adult men who decline VCT (of whom ~ 200 will be HIV+ positive and 800 HIV- negative), and ~ 800 HIV-positive men, aged 15-49, resident in Rakai communities in southwestern Uganda. We have excluded uncircumcised boys under age 15, because the low HIV incidence in such young males would not add to the study power, and we wish to establish safety and efficacy at older ages, before assessing circumcision in boys under age 15. The RCCS participants will be ~6000 women and ~ 3000 men enrolled in the cohort.

In the trial, men consented for screening and if found to be eligible, they consented for enrollment. Consenting men were randomly assigned to an immediate circumcision arm (intervention arm) in which they were offered the service within approximately 1 month of enrolment, and to a delayed circumcision (control arm), in which they were offered circumcision approximately 24 months after enrolment. Circumcision is carried out by trained and supervised physicians. Participants are monitored after surgery to maximize care. Men in both arms are interviewed and asked to provide biological samples at enrolment and at 6,12 and 24 month visits

Interview information is obtained at baseline, time of surgery and at each follow up visit and includes sociodemographic characteristics, risk behaviors (e.g., number and type of partners, condom use, sexual networks, alcohol use, coercive sex), health information including symptoms of STDs or penile pathology (e.g., discharge, dysuria, GUD, balanitis, post-operative infection or bleeding), and symptoms suggestive of OIs or AIDS (e.g., weight loss, chronic diarrhea, cough, KS etc). Most questions have already been tested in prior Rakai Health sciences Program surveys.

Blood samples are collected from men for detection of HIV, STDs and hemoglobin as per protocol . Male urine sample is collected for detection of STDs with other funds. Prior to surgery, subpreputial swabs are obtained for assessment of STDs as per protocol and men reporting GUD are asked for an ulcer swab for M-PCR with other funds. The foreskin of circumcised men are retained for histopathology.

Subjects are identified by a permanent study ID number on all forms and samples, and a polaroid photograph is used to insure identity during follow up.

## Recruitment and Consent

Participants were recruited through community mobilization meetings and home visits. With the assistance of our Community Advisory Board (CAB), we inform the population of the research. Following contact with village leaders, we conduct open community meetings during which the community is informed of the nature of the trial, including randomization into one of two arms, and the possible risks and benefits of male circumcision. Following the community informational meetings, all age eligible men aged 15-49 residing in the community (identified from Rakai Health sciences Program data files) or non-cohort participants (“walk ins”) were invited to go to the Kalisizo Clinic or central locations in the communities (“hubs”) for screening and enrollment. They were clearly informed that participation is entirely voluntary and that they would not forego any Rakai Program services (condoms, VCT, access to Rakai Health sciences Program clinics) should they decline enrollment into the trial. The Screening and Enrollment Consents are given in Appendix 1, Forms I and I.a.

All prospective volunteers were offered VCT. After completing a screening consent and the screening procedures, men who did not meet the NIH trial criteria,(HIV- accept VCT), but are otherwise eligible for the Gates trial, were asked to provide written consent for enrollment into the Gates trial. The consent process included oral group or individual presentations with a video (where possible) and an opportunity to ask questions during preliminary health education. Participants were given an opportunity to read the consent if they are literate, or to have the consents read to them if illiterate. Individual consent was then obtained. The consent included information regarding the design of the trial, requirements for participation including followup and data/sample collection, the risks and potential benefits of circumcision, and the freedom to refuse in part or in whole, or to withdraw from the trial, without loss of benefits and privileges. Contact names of physicians and phone numbers were provided for questions or complaints**.** After randomization, men completed a consent comprehension test to ensure that they understand the trial and to correct any misunderstandings.

Men who are unsure about participation, but who did not refuse outright, they were told that they should take time to consider their decision. They were be re-contacted after 2-3 days to determine whether they decide or decline to participate.

As is standard operating procedure, consent forms were translated into Luganda, and back translated into English. A certificate of translation was obtained. Subjects were asked to sign or thumb print the consent document, and consent was independently witnessed. Enrolment of unemancipated minors aged 15-17 required both the assent of the parent/guardian and independent individual consent by the minor. A copy of the signed form was retained by the subjects, and original forms maintained in safe, locked stores at Program offices.

Consent is obtained on screening, enrollment and prior to surgery**.**

## Ethnic Groups and Minors

Residents of Rakai District are all Black African, and belong to several tribal groups, of which the Baganda, Banyarwanda, Bakiga and Banyanchole are the largest. Men were enrolled regardless of tribal affiliation.

As indicated above, the study included respondents aged 15-17. This in keeping with recent NIH standards that minors be included in research related to conditions which affect their health. In Rakai, HIV, STDs and genital tract conditions represent health problems at young ages. Safety and healing following circumcision needs to be examined in adolescent males, since in countries such as Kenya, there is growing evidence that males in this age group are seeking out the procedure.

Emancipated adolescents, defined as persons aged 15-17 who were heads of households, or who lived independently of their parents or guardians and were not in school, were asked to provide individual informed consent. Unemancipated minors aged 15-17 were likewise asked to provide individual informed assent, but their enrolment was alsocontingent on written consents by the parent or guardian. (Please note: if the parent or guardian consented but the minor did not assent, that minor was not enrolled.)

## Ethical Justification for Inclusion of HIV-Positive Men and Men who Decline VCT

Only HIV-negative men will provide person years of observation to assess the efficacy of male circumcision for prevention of HIV acquisition. However, HIV-positive men accepting circumcision will be individually randomized to the early circumcision intervention arm or to the control (delayed circumcision) arm.

### Direct Benefit of Circumcision for HIV+positive Men:

There are substantial data that circumcision may reduce the rates of GUD, urethral discharge, balanitis, syphilis and HSV-2 in HIV positive men, and thus confer a direct benefit to them. Since such infections are more common in the HIV positive, and are exacerbated by HIV infection, we believe there is evidence of potential benefit to HIV+ men. This results in equipoise and justifies their inclusion to assess STD benefits in this subgroup.

### Ethical Dilemmas that would arise if HIV positive men were excluded:

The NIH sponsored trial only enrolls HIV-negative men. We believe that enrollment of HIV-positive men into the Gates sponsored trial is an ethical imperative, because these trials will be conducted within rural communities in which persons know one another and exchange information on their participation in Rakai Program studies. It is important that the community context of this trial be born in mind. These men are resident in the community, and both their circumcision status and study participation or non-participation based on HIV-related eligibility criteria cannot be concealed. Therefore, the following ethical dilemmas would arise if HIV positive men were excluded from trial eligibility:

**1) *Societal Stigmatization:*** Excluding HIV positive would be stigmatizing, since their rejection from trial eligibility would automatically imply that they were HIV-infected. Thus, if community residents interpret exclusion from the trial as a stigma of HIV-infection, it would essentially mean public and involuntary disclosure of the excluded individual’s HIV status, and is ethically unacceptable.

**2) *Informing participants of the reasons for their selection:*** The Belmont Report explicitly recommends that subjects be informed about how they are selected,^119^ and this is reiterated in OHRP regulations (CFR 46). Therefore, we could not conceal that HIV status is a criteria for eligibility.

**3) Masking HIV status on copies of consent forms:** The same consideration applies to copies of consent forms provided to participants. It is imperative that the consent copy should not directly or indirectly disclose an individual’s likely HIV status, because this would be a breach of confidentiality and a cause of social harm. We have, therefore, been careful to mask a participant’s HIV status and trial of enrollment by using a single, common Gates and NIH screening consent form, and coded enrollment forms that do not disclose trial of enrollment or HIV status (forms I and I.b.)

### Safety and Programmatic Considerations

**1). *Surgical Risk:*** If there are greater surgical risks of circumcision in HIV+ positive men (e.g., infection, delayed wound healing), it is important that these risks be identified to define necessary precautions and to guide surgeons on appropriate patient management in future programs.

**2) *Programmatic Considerations:*** It is important to determine the safety of circumcision in HIV positive men, and mechanisms to enhance safety, since such individuals would, in all likelihood, be included in many future large scale circumcision programs. If programs had to screen all candidates for surgery and exclude HIV positive men because the safety of circumcision in HIV-infected persons was unknown, the programs would confront the same ethical dilemmas as outlined above. Therefore, assessing safety in HIV positive men is a public health priority.

**3. *Assessment of Risks to HIV- Female Partners****:* If it is found that wound healing is delayed in HIV positive men it could increase risk of male-to-female transmission during the postoperative wound healing period. This information is critically needed to design strategies to maximize safety in future programs.

**4. *Seeking of unsafe circumcisions*:** If HIV positive men were excluded from the trial and only HIV negative men received circumcision from the Project, circumcision would rapidly be perceived in these communities as indicating that a man has a high probability of being HIV negative. We are very concerned that some HIV positive men would seek circumcision elsewhere, including unsafe sources, as a means of concealing their true HIV status. In addition, control arm men may be more likely to cross over and undergo surgery outside the project, in order to allay any suspicion that they were denied circumcision because of their serostatus.

**5. *Disinhibition:*** If circumcision was perceived as only being offered to HIV negative men, this could engender higher risk behavior and lower condom usage among circumcised men and their partners.

We firmly believe that exclusion of HIV positive men from these trials would create major ethical dilemmas and preclude the investigation of important safety issues critical to future programmatic and clinical practice. Thus, we believe that these trials can only be ethically conducted in a community setting if both HIV-negative and HIV-positive men are enrolled*.* We anticipate that the DSMB would carefully scrutinize data on circumcision of HIV positive men, and if the DSMB found evidence of risk to either to HIV positive men or their partners, the study would modified according to DSMB recommendations **The findings at the December 19, 2006 and March 1, 2007 reviews suggested non-significant higher rates of male-tofemale HIV transmission in the intervention arm, largely among couples who resumed sex prior to wound healing. The moderate and severe surgical complications among HIV+ men (3.2%) were comparable to those in HIV-negative men (3.6%), although wound healing may have been somewhat slower in the HIV+ men. Circumcision reduced the rates of GUD in HIV+ men.**

## Ethical Considerations for Inclusion of Women.

The Study Section recommended exclusion of women from the trial, whereas we believe it is imperative to include women for the following reasons:

### Potential Benefits to Women:

**1) *Female HIV acquisition as a study endpoints:*** The inclusion of female partners of circumcised men and controls allows assessment of the effects of circumcision on male-to-female HIV transmission. This is an important direct benefit (see Background and Significance ) and is a feasible end point for which there is equipoise (see Methods).

**2) *Female STD acquisition as a study end point:*** The female STD acquisition end point requires inclusion of all women, irrespective of their male partner’s HIV status. There is evidence that this is a direct benefit to women (Background and Significance), measurement of STD end points is feasible (see Methods), and there is equipoise.

### Ethical Dilemmas that Arise if Women were Excluded:

OHRP guidelines require the “inclusion of both women and men in research, both to insure that they receive appropriate share of benefits of research, and that they do not bear disproportionate burden”.^146^ Thus, inclusion of women in this study is consistent with Federal policies, since could benefit from reduced HIV and STD risks. The Rakai cohort has always included resident consenting adults of both sexes, and if women were excluded from the trial, the long established community relationships and bonds of trust could be compromised. We believe that exclusion of women is not a viable option in the context of Rakai population-based studies, and that the trial cannot be conducted if women are excluded. The ethical rationale for inclusion of women is as follows:

**1) *Potential Stigmatization:*** The male-to-female HIV transmission end point will be assessed in couples where the male is HIV+. However, we could not selectively identify and follow up such couples because it would highly stigmatizing. We will maintain surveillance of consenting female partners (wives and permanent consensual partners) of men enrolled in the trial, but we will not enroll discordant couples *per se*. Our rationale is that adult participants of both sexes provide individual informed consent on enrollment into the cohort, and they are free to accept VCT by individual choice. Free, confidential VCT is offered to individuals and couples, and all participants are strongly encouraged to share HIV results with their partners, and to receive couples counseling. However, specific enrollment of HIV-discordant couples, ethically requires that both parties accept VCT, and in our experience, such a requirement would be a disincentive both for acceptance of VCT and for enrollment. Our VCT program is successful because it emphasizes personal choice and is not coercive or restrictive. This is, in our view, the only ethically defensible approach to community-based research and service, and is concordant with Ugandan Ministry of Health policy.

**2)*Omission of derivative benefits to women and public health rationale for circumcision:*** Exclusion of female partners of enrolled men would preclude assessment of potential derivative benefits for women, and this would weaken the public health rationale for future introduction of circumcision programs.

**3) *Inability to identify and address potential harm to women.*** Potential harm might result if delayed wound healing in HIV+ men resulted in higher viral shedding for some period following surgery, or if circumcised men adopted increased risk behavior. Including women would enable the study to identify such harm, and in consultation with the DSMB, to devise corrective measures or if necessary, stop the trial.

Given these considerations we proposed to include consenting women*.*

## Potential Risks to Participants

**Risks of circumcision:**

**The rate of complications following circumcision reported in the clinical literature is less than 2**% and include transient hyperesthesia, hematoma, bleeding and infection. Damage to the glans or shaft is extremely rare. Physical activity or intercourse early after surgery could exacerbate bleeding, bruising or infection. Intercourse before wound healing could increase the risk of HIV acquisition. Allergy, including anaphylaxis can occur with local anesthesia and antiseptics. Drowsiness or unsteadiness can occur with pain medications.

**Confidentiality and social harm.**

There is a risk of breech of confidentiality if patient records, interviews or lab results are revealed to third parties. There is a risk that misidentification could cause an individual to be given an incorrect diagnosis or inappropriate treatment, or be inadvertently denied indicated treatment. There is a risk that knowledge of positive HIV results received via voluntary counseling and testing (VCT) could lead to distress, marital disruption or partner abuse.

**Disinhibition:**

If circumcised men believe themselves to be at reduced risk of HIV or STD infections, they may adopt higher risk behaviors. If the copy of the consent form retained by HIV-negative men enrolled in the NIH trial documented HIV status, this “proof” of negative status could be used to negotiate unsafe sexual behaviors. To avoid this dilemma, the copy of the consent provided to participants does not indicate HIV status directly, or indirectly (e.g., by specifying NIH or Gates trial enrollment).

**Risk related to biological specimen collection.**

Blood and urine collection is minimally invasive and entails minimal risk. Foreskins are saved for separate studies: the risk of collection is summarized under risks of circumcision.

## Procedures for Protecting Against Risk.

**a Risks of circumcision:**

The risk of complications during or following circumcision are minimized by careful training and certification of physicians, supervision by an experienced urologist (Dr. Watya), and referral/exclusion of men with anatomic abnormalities of the genitalia. A physician at the Kalisizo Hospital is available to assist with surgical complications. The hospital is only minutes away from the Rakai clinic facility. The risk of infection is minimized by: i) In men with evidence of current infection, surgery is deferred, and men are treated with antibiotics until infection has resolved. ii) Surgery is performed in a dedicated surgical facility, with strict adherence to aseptic procedures, autoclaving of all instruments, and careful dressing and monitoring of wound healing. iii) Any postoperative infection is treated with antibiotics and, if indicated, men are hospitalized at project expense for wound care. The risks of bleeding or bruising are minimized by: i) careful surgical technique and attention to bleeders, ii) use of an occlusive dressing, iii) postoperative bed rest and provision of loose garments and transport home, iii) encouraging participants to refrain from physical work and activity for approximately 3-5 days, and to refrain from intercourse until complete wound healing is certified. Men are provided with clear information on risks of premature resumption of physical work, and compensation is paid for work time lost (the equivalent of $15, to enable them to take the day of surgery, and the subsequent 3-5 days, off work). Potential risk of HIV are minimized by advising men to refrain from intercourse until the wound is healed, and promotion of condoms when intercourse is resumed. We provide detailed information and follow up for subjects to minimize risks. Postoperative pain is controlled by analgesia and limitation of activity. Men experiencing hyperesthesia are told this is usually transient, but if it does not resolve within a week, they are referred to the urologist for free care.

**b Confidentiality and social harm**

The Rakai Health Sciences Program has, for fourteen years, maintained full confidentiality of participant records. Informed consent documents are retained in locked filing cabinets and store rooms, accessible only to senior investigators or designated staff. . Case report forms retain participant’s names until completion of analysis All questionnaires are stored in secure, locked facilities in the field station in Kalisizo and permanent stores in Entebbe. Only designated staff have access to these records. Photos used to identify persons are kept in locked cabinets and are under the control of field supervisors during surveys. All computerized data bases only contain study ID numbers. . Electronic records containing confidential or sensitive information only contain the study ID # without the participant’s name. Files of lab results are maintained in a separate safe computer file, with study ID numbers, and contain no personal identifiers.

Misidentification is avoided by the photo ID and by use of unique, alphanumeric check digit ID numbers. The check digit number avoids data entry errors. All documents and samples are labeled with pre-printed study numbers to avoid transcription errors. Lab results are double entered to avoid data keying errors.

c. HIV Voluntary Counseling and Testing (VCT): protection of confidentiality

Individuals and couples in the Rakai cohort are offered VCT, and those who accept are flagged for expeditious assay. For the circumcision trial, rapid HIV tests were used to assess current HIV status for cohort participants and to provide a provisional diagnosis for non-cohort participants (walk ins). Two rapid tests were used. If both were negative, the participant would enrolled if he declined VCT, but confirmatory double EIAs and, where appropriate, western blot are run. The participants were told that the rapid tests must be regarded as provisional, until the EIA/Wb results are available. If either or both rapid tests was/were positive, the participant was told to wait until further confirmatory testing (EIA/WB) was done. The following procedures are followed to minimize risk of social or psychological harm that might result from breech of confidentiality. HIV result letter used by the counselor to inform the participant, contains the study ID# and the participant’s name to insure correct identification, but the HIV result is coded so as to be un-interpretable by a third party.

Participation in VCT (both individual and couples counseling), although strongly encouraged, is entirely voluntary. Counselors provide pre-results counseling for participants and inform them of the potential for social harm, and advise them on how to mitigate such consequences. Results are provided in private to individuals or couples (if both individuals have elected to participate in couples counseling). If recipients opt for individual counseling, they are encouraged to inform their partners of the test results but, in accord with Ugandan Government policy, no results are revealed to third parties, including spouses, without an individual’s express, written permission.

d. Disinhibition:

To avoid false expectations of protection by circumcision we carefully informed participants that the efficacy of the interventions was unknown at that time, that monogamy with an HIV- partner or consistent use of condoms are the only proven methods of preventing infection, and that avoidance of high risk behaviors is imperative to reduce HIV risk. (Condoms are provided free of charge by the Project and we recommend that condoms be used by all men). Because we have designed the study within Rakai communities, all members of the community - men and women - are informed via town meetings of the need to continue other safe sex practices. The message is reiterated during each community town meeting for the duration of the study. In addition, this information is given at each individual trial and cohort follow up.

To minimize disinhibition among men in the NIH trial, and to prevent the use of copies of the consent form as “proof” of HIV-negative status, the consent copy retained by participants does not indicate HIV status. Disinhibition is also minimized by intensive health education and monitoring of risk behaviors both via surveys and by qualitative research methods.

We monitor behaviors carefully during the trial and if an excess of risk behaviors is observed, the DSMB will determine whether this can be rectified, or whether the trial should be terminated.

**e Risks of biological sample collection.**

Venipunctures and genital ulcer swabs are collected by highly trained and experienced Rakai Health sciences Program survey and clinical personnel. Over 80,000 serological specimens and 80,000 urine specimens were collected during the Rakai STD Control Trial for HIV Prevention, with no significant adverse event.

## Study Benefits to Participants

**a Health Care:**

Residents of Rakai Health sciences Program study communities, whether or not they have agreed to participate in any Rakai study, are provided with health education via town meetings and are offered condoms.

All residents also have access to Rakai Health sciences Program mobile clinics for free general health care and symptom-based STD treatment at the time of our annual survey rounds.

All residents have access to the Rakai Health sciences Program fixed clinic in Kalisizo for STD treatment; study participants are offered STD treatment for free in the fixed clinic.

All HIV positive residents, whether or not they are study participants, are offered free health services and treatment for OIs through the Rakai Health sciences Program fortnight HIV clinic in our fixed facility in Kalisizo, and in two mobile hubs which service distant communities.

In addition to the benefits above, study participants are offered free, confidential individual and couples voluntary counseling and testing.

**b. STD testing and treatment provided to participants**

In addition to the symptom-based STD treatment offered to all study community residents through our mobile clinics at the time of each study round, circumcision trial participants in both arms are routinely tested for syphilis, and offered treatment on the basis of results. In addition, participants are examined and any pathology diagnosed clinically is treated. We use highly effective, single dose observed therapy, as we did in the previous Rakai Program STD control trial.^78^ The drugs include azithromycin, ciprofloxacin, cefixime, metronidazole and IM benzathine penicillin. Participants in both study arms are also provided with HSV-2 suppressive therapy, if there is evidence of active herpetic ulceration at the time of the survey or prior to circumcision.

**c. General health care:**

At the time of each survey round, Rakai Health sciences Program mobile clinics offer free general health care, including anti-malarial and antibiotics as required, to all study community residents. Symptom-based care includes treatment for respiratory conditions, thrush, fever, skin conditions, pain and diarrhea.

**d. Treatment for Opportunistic Infections**

As indicated above, all HIV positive residents of Rakai study villages are offered free care at bi-weekly clinics held in our Kalisizo facility and in two mobile hubs which service distant villages. We provide prophylaxis and treatment of OIs, and ARVs provision via the President’s Emergency Program for AIDS Response (PEPFAR). Care is provided when appropriate and feasible. Given that most HIV+ men enrolled into the Circumcision Trial will not become eligible for ARVs during the course of the 2 year study follow-up (ie., most are unlikely to meet CD4 or viral load criteria during this time period), we did not expect that the availability of ARVs would have an effect on study power to assess circumcision safety, STD acquisition or HIV transmission.

e. Provision of free circumcision:

Men in the intervention arm were offered free circumcision within one month of enrolment.

Men in the control arm are provided with free circumcision after 24 months follow up as a service, contingent on DSMB assessment of safety. They were told that information on efficacy was not available, and that they could defer circumcision to the end of the study (2007), when the last men enrolled into the two study arms complete 2 years of follow up. (Please note: the DSMB stopped the NIH funded trial after interim analysis showed more that 50% efficacy.)

Therefore, HIV-negative uncircumcised men in the control arm of the NIH and Gates sponsored trials will be offered surgery. HIV+ men randomized to the intervention arm, and HIV+ controls who have completed 24 months follow up will be offered surgery. Men initially randomized to the intervention arm who failed to return for surgery within six months of enrollment and were classified as crossovers, will also be offered surgery

**f. Condoms**

Condoms are provided free of charge to study participants via the survey teams, health education team, HIV counselors and our community mobilizers. The former reside permanently in the study communities.

**g. Considerations Regarding Provision of Antiretroviral Therapy**

We are enrolling HIV-positive men and it is inevitable that HIV seroconversions will occur, despite condom promotion and possible efficacy of the interventions. We have support from PEPFAR to provide ARVs to eligible participants.

## Compensation for Participants

We proposed to provide $30.00 (approximately 52,500/= Uganda shilling) as compensation for time lost due to surgery and the time required for screening/ enrollment and follow-up. All men received $3.00 compensation at time of enrollment, and for each scheduled visit at 3-6 weeks, 6, 12 and 24 months. Men in the immediate circumcision arm received $15.0 compensation distributed as $5.00 on day of surgery, and $5.00 at each of the 48 hour and 7 ± 2 days visits. Control arm men will receive $15.00 compensation for time lost due to surgery. Men selected for frequent follow up will be compensated with 3$ for every added visit. Based on discussions with our Community Advisory Board, the proposed amount was deemed to fairly recompense participants, without being a coercive inducement. Experience during stage one suggested that this distribution of compensation can maintain high compliance rates and avoid disinhibition that might occur with larger lump sum payments.

Men will be invited to attend focal points or “hubs” located in the communities and will be compensated for transport cost.

## Benefit-Risk Ratio

The risks of the proposed Circumcision Trial are reasonable in relation to the benefits. Adult circumcision entails relatively minor surgery under local anaesthesia, with low rates of complications. The training and supervision of physicians performing the procedure and the conditions under which it will be performed should minimize these risks. Stage 1 results showed that circumcision offered in the Rakai study clinic is safe . The individual benefits of the intervention are potential protection from STDs in HIV-positive men, and protection from HIV and STDs in HIV-negative individuals, and improved penile hygiene. There are substantial societal benefits. If circumcision is efficacious, it would provide critical information for policy and development of programs for HIV prevention. Because circumcision is a one time procedure, such benefits could be life long.

The risks of breech of confidentiality are minor, and the Rakai Program has put in place numerous safeguards to prevent such breeches and to avoid misidentification errors. The benefits also include access to HIV results and counseling through VCT, free condoms and health care.

The risk of disinhibition cannot be determined prior to study initiation, but will be offset by careful education and patient instruction. We have not observed disinhibition in our previous intervention trials.

# PUBLICATION OF RESEARCH FINDINGS

Results of the research are communicated to the Ugandan Ministry of Health and to study communities.

A Publication Committee consisting of senior investigators will be formed to decide on topics for papers, and allocation of responsibility for manuscripts. First authorship will be given to the investigator who takes primary responsibility for specific analyses and writing of papers. Papers or abstracts will be submitted only after review by the Publication Committee.

Note: More frequent post operative follow up will done form the HIV positive men and sub sample of HIV negative men to assess wound healing. (Weekly until complete wound healing)

**Table 1. Time Line**

| **Activities (Project months)** | **Year 1** | **Year 2** | **Year 3** | **Year 4** | **Year 5** |
| --- | --- | --- | --- | --- | --- |
| **Initial enrollment**  **(~ mths 1-6)** | **xxxxxx** |  |  |  |  |
| **Pause for Assessment of acceptability/safety (~ mth 6-7)** | **x** |  |  |  |  |
| **Continuing Enrollment/Randomization**  **(~ mths 8-27)** | **xxxx** | **xxxxxxxxxxxx** | **xx** |  |  |
| **Intervention Circumcisions**  **(~ mths 9-27)** | **xxxx** | **xxxxxxxxxxxx** | **xxx** |  |  |
| **6 month follow up**  **(~ mths 6-32)** |  | **xxxxxxxxxx** | **xxxxxx** |  |  |
| **12 month follow up**  **(~ mths 20-38)** |  | **xxxx** | **xxxxxxxxxxxx** | **xx** |  |
| **24 month follow up**  **(~ mths 32-50)** |  |  | **xxxx** | **xxxxxxxxxxxx** | **xx** |
| **DSMB safety monitoring**  **(~ mths 19,31)** |  | **x** | **x** |  |  |
| **DSMB Interim Analyses**  **(~ mths 40, 56)** |  |  |  | **X** | **X** |
| **Circumcise Controls**  **(~ mths 24-56)** |  |  | **xxxxxxxxx** | **xxxxxxxxxxxx** | **xxxxxxxxxxxx** |
| **Analysis and reports**  **(~ mths 51-60)** |  |  |  |  | **xxxxxxxxxxxx** |

**Table 2. Summary of Forms for stage 2 of the Trial**

| **Record Forms** | **Contacts** | | | | | | | |
| --- | --- | --- | --- | --- | --- | --- | --- | --- |
|  | **1** | **2** | **3** | **4** | **5** | **6** | **7** |  |
| **Pre-Enrollment interview for cohort participants interviewed within past 6 months (02a/48)** | **x** |  |  |  |  |  |  |  |
| **Screening Consent (I)** |  | **x** |  |  |  |  |  |  |
| **Enrolment Interview and Exam (02b)** |  | **x** |  |  |  |  |  |  |
| **Eligibility verification form (22)** |  | **x** |  |  |  |  |  |  |
| **Enrolment Consent (I. b.)** |  | **x** |  |  |  |  |  |  |
| **Interview for non-cohort participants^1^ and for cohort participants interviewed > 6 months previously (02a)** |  | **x** |  |  |  |  |  |  |
| **Randomization form (18)** |  | **x** |  |  |  |  |  |  |
| **Post-randomization Information sheet (II)** |  | **x** |  |  |  |  |  |  |
| **Surgical consent (III)** |  |  | **x** |  |  |  |  |  |
| **Pre-operative Interview/ Exam (03)** |  |  | **x** |  |  |  |  |  |
| **Surgical record (04)** |  |  | **x** |  |  |  |  |  |
| **Postoperative patient information sheet (46)** |  |  | **x** |  |  |  |  |  |
| **Post-operative visit forms** |  |  |  | **x...** |  |  |  |  |
| **Follow up Visits 6, 12, 24 months (12)** |  |  |  |  | **x** | **x** | **x** |  |
| **Adverse Events Reporting Form (11)** |  |  | **x** | **x** | **x** | **x** | **x** |  |
| **Protocol deviation form (13)** |  | **x** | **x** | **x** | **x** | **x** | **x** | **x** |
| **Withdrawal form (14)** |  | **x** | **x** | **x** | **x** | **x** | **x** | **x** |
| **Missed visit form (15)** |  |  | **x** | **x** | **x** | **x** | **x** | **x** |
| **Control subjects consent for surgery (IV)** |  |  |  |  |  |  |  | **x** |

**^1^Men who were not previously enrolled in the cohort or cohort participants last interviewed > 6 months before enrollment will complete the interview after enrollment into the trial**

Table 4. Number of AEs, exact confidence intervals and 1-sided p values for all AEs and for moderate/serious AEs for the first 100 circumcision surgeries.

| **Number of events /**  **Estimated rate (%)** | **Exact 95% Confidence intervals** | **Exact 1-sided p-value for testing vs. 2% rate** | **Exact 1-sided p-value for testing vs. 0.5% rate** |
| --- | --- | --- | --- |
| **0** | **0.00-3.52** | **.1326** | **.6058** |
| **1** | **0.05-4.92** | **.4033** | **.3942** |
| **2** | **0.36-6.60** | **.5967** | **.0898** |
| **3** | **0.82-7.99** | **.3233** | **.0141** |
| **4** | **1.38-9.43** | **.1410** | **.0017** |
| **5** | **1.99-11.03** | **.0508** | **.0002** |
| **6** | **2.65-12.16** | **.0155** | **<.0001** |
| **7** | **3.33-13.49** | **.0041** | **<.0001** |
| **8** | **3.52-14.84** | **.0009** | **<.0001** |

**Table 5. Male HIV Incidence by Age**

| **Age groups** | **15-19** | **20-29** | **30-39** | **40-49** | **All** |
| --- | --- | --- | --- | --- | --- |
| **HIV Incidence/100 py in uncircumcised men (observed)** | **1.1** | **2.1** | **1.9** | **1.8** | **1.8** |
| **Estimated number of men enrolled per arm** | **435** | **988** | **582** | **495** | **2,500** |
